# Developing an understanding of networks with a focus on LMIC health systems: How and why clinical and programmatic networks form and function to be able to change practices: A realist review

**DOI:** 10.1016/j.ssmhs.2023.100001

**Published:** 2023-10

**Authors:** Katherine Kalaris, Mike English, Geoff Wong

**Affiliations:** a Health Systems Collaborative, Kellogg College, University of Oxford, Peter Medawar Building for Pathogen Research, 3 South Parks Road, Oxford OX1 3SY, United Kingdom; b Health Systems Collaborative, Nuffield Department of Medicine, University of Oxford, Peter Medawar Building for Pathogen Research, 3 South Parks Road, Oxford OX1 3SY, United Kingdom; c Nuffield Department of Primary Care Health Sciences, University of Oxford, Radcliffe Primary Care Building, Radcliffe Observatory Quarter, Woodstock Road, Oxford OX2 6GG, United Kingdom

**Keywords:** Networks, Health systems, Realist review, Realist synthesis, Network formation, Network functioning, MeSH: delivery of health care, LMICs

## Abstract

Networks are an increasingly employed approach to improve quality of care, service delivery, and health systems performance, particularly in low-and-middle income country (LMIC) health systems. The literature shows that networks can improve the provision and quality of services and health system functioning but there is limited evidence explaining how and why networks are established and work to achieve their reported results. We undertook a realist review to explore this. The objective of this realist review was to develop a programme theory outlining the underlying mechanisms and interactions of contexts that explain how and why a network’s set-up and function enable high-quality care and services and improved clinical outcomes in LMIC health systems. We followed Pawson’s five steps for realist reviews. The search strategy was based on a previously published scoping review with additional searches. Literature was selected based on its relevance to the programme theory and rigour. Context-mechanism-outcome configurations were developed from the extracted data to refine the initial programme theory with causal explanations. Theories on social movements and organisations supported the identification of mechanism and brought additional explanatory power to the programme theory. The programme theory explains how networks are initiated, formed, and function in a way that sets them up for network leadership and committed, engaged, and motivated network members to emerge and to change practices, which may lead to improved quality of care, service delivery, and clinical outcomes through the following phases: identify a problem, developing a collective vision, taking action to solve the problem, forming purposeful relationships, linkages, and partnerships, building a network identity and culture, and the creation of a psychological safe space. This deeper understanding of networks formation and functioning can lead to a more considered planning and implementation of networks, thereby improving health system functioning and performance.

## 1 Introduction

Health systems are complex systems and various efforts have been made to better understand them through classifications and typologies (Bohm et al., 2013, Cockerham, 1992, Stevens, 2005, Roemer, 2001, Santerre and Neun, 2010, Rothgang et al., 2005, Freeman and Frisina, 2010). However, much of the work around health system classification has been focused on high-income (HIC) countries and member countries of the Organisation for Economic Co-operation and Development and therefore does not adequately take into account the specificities and complexities of health systems in other settings, particularly in low-and-middle income countries (LMIC) (dedeCarvalho et al., 2021). LMICs according to the World Bank include countries with a gross national income below 13,845 USD (World Bank Country and Lending Groups, 2023). Health systems, regardless of their classification, are plagued with problems of sub-optimal functioning (Pittalis et al., 2021, Rossiter et al., 2017).

Networks are an increasingly employed approach to tackling clinical and health systems challenges with interest and momentum from national ministries of health and global and local public health stakeholders. Examples of networks exist across both HIC and LMIC health systems. There are many different types and definitions of health systems networks; we define networks as “groups of facilities and/or healthcare affiliated stakeholders linked formally or informally, horizontally or vertically, through programs, interventions, activities, or initiatives.” (Kalaris and Wong, 2023) Stakeholders include but are not limited to all types of providers, technicians, government officials, professional associations, Non-Government Organisations, and donors. These networks form when “groups of health system actors from across level and sectors of care, entities, and geographies come together in a distinct way and work together with the aim to improve service delivery, quality of care, and/or health system functioning.” (Kalaris and Wong, 2023) For example, the Clinical Information Network in Kenya links county level hospitals to improve generation and use of health systems data to strengthen the provision of care (English et al., 2017). The formation of the network was a collaborative effort between hospitals, the government, a research institution, a university, and a professional association. In Metro Manila, Philippines, the Quirino Respected Partners network created trusted relationships among providers at different levels and sectors of care by linking a tertiary level public hospital and private and public midwifery clinics, which enabled the decongestion of the tertiary hospital and improved quality of care for maternal and newborn care (Vergara et al., 2020). This network was created from the bottom-up by a clinician at the tertiary level hospital. A provincial level network in Cotopaxi, Ecuador strengthened the relationships between levels of care, including formally integrating community health workers and traditional birth attendants to provide better access to and coordinated quality of care for mothers and newborns (Broughton et al., 2016). The network was initiated by a donor funded project. Networks in this realist review are focused on clinical practice and implementation of health programmes with coordinated actions around a shared agenda or goal. There may be overlap between these networks and other types of cross-organisational collaboration, which were not included in the review. For example, communities of practice, which are made up of people with a shared interest or problem who exchange knowledge and information to deepen their learning and understanding of an issue, may support members’ independent actions at their own facility or organisation and not necessarily implementation of coordinated actions around a shared agenda or goal (Wenger et al., 2002).

### 1.1 Rationale for the review

The existing literature on health system networks is diverse and mainly focused on clinical achievements of the network with limited documentation in the literature on how they form and function. There are few studies of how any type of network evolves (Provan et al., 2007) or robust descriptions of them, which enable the exploration of how and why networks are able to do (or not) what they set out to achieve. This realist review developed a programme theory drawing on the literature and sociological and psychological theory, that offers an explanation of how and why networks are initiated, form, and function in a way that sets them up to be able to change practices in order to improve and provide high-quality care in LMIC health systems. The programme theory could provide useful insights into the key phases a network may go through in its development for governments and organisations implementing or interested in implementing a network in their health system. This is important because “Only by developing an understanding of how networks can successfully be formed and evolve can network and public health planners, funders, and participants begin to make the decisions required to ensure that networks fulfil their promise.” (Provan et al., 2011) Furthermore, because networks often form from the bottom-up or organically, articulating some of the processes and drivers of how that occurs is important for understanding how they achieve their outcomes.

### 1.2 Objectives and focus of the review

This realist review focuses on clinical and programmatic networks in LMIC health systems. This review builds on a scoping review that developed a framework broadly describing the key components of a network and mapped network uses, purposes, and stakeholders with a focus on the LMIC literature. The scoping review’s mapping of network uses and purposes identified improving and providing high-quality care and services as the main use and purpose of networks, which helped to guide the objectives and questions for this realist review (Kalaris and Wong, 2023).

After initial immersion in the literature and developing a broad conceptualization of networks from inception to impact we felt there was an opportunity to focus on a network’s initiation and establishment. The process of focusing is described in the methods section. This also specified the review objectives and questions on the set-up and functioning of networks.

#### 1.2.1 Review objectives

Develop a programme theory outlining the underlying mechanisms and interactions of contexts that explain how and why a network’s set-up and function enable high-quality care and services and improved clinical outcomes in LMIC health systems.

#### 1.2.2 Review questions

• How do networks set-up and functioning enable them to improve and provide high-quality care and services in LMIC health systems?
• Why are networks set-up and function in these ways to improve and provide high-quality care and services in LMIC health systems?
• What are the mechanisms in a network’s set-up and functioning that provide a platform for the improvement and provision of high-quality care and services in LMIC health systems?
• What contexts trigger the mechanisms within a network’s set-up and functioning that lead to improvements and the provision of high-quality care and services in LMIC health systems?

## 2 Methods

### 2.1 Rationale for a realist review

While the literature on health system networks is diverse, it is mostly focused on reporting clinical outcomes achieved by the network and less on how the network develops and works to produce those outcomes. Generating an understanding of how networks develop and function can help to inform future set-up, functioning, or scale-up of networks. The realist approach is particularly appropriate to develop this type of understanding as its core aim is to generate an understanding about a complex intervention (Pawson et al., 2005).

Networks can be considered a complex intervention because they are made up of many interacting components (Greenhalgh and Papoutsi, 2018). They exist within complex systems, health systems, which are “composed of many interdependent, heterogeneous parts that self-organize and co-evolve.” (Lanham et al., 2013) Realist research works well with complex social programmes or interventions (Pawson et al., 2005, Pawson, 2013).

Few realist reviews have studied networks in health systems (Aunger et al., 2021a, Aunger et al., 2021b, Zamboni et al., 2020) or focused on LMIC health systems in general. Using the realist approach for this setting is an opportunity to generate new insights that could inform research and programme implementation beyond this scope of work. Networks are centred on interpersonal relationships (Mandell and Keast, 2008) and therefore need an approach – such as a realist approach which is focused on the reasoning, response, and reaction of those involved in the phenomena under study. Therefore, this realist review contributes to the knowledge base methodologically, by employing the realist approach to evidence synthesis to an understudied topic, and content-wise with a focus on networks in LMIC health systems.

**Fig. 1:**
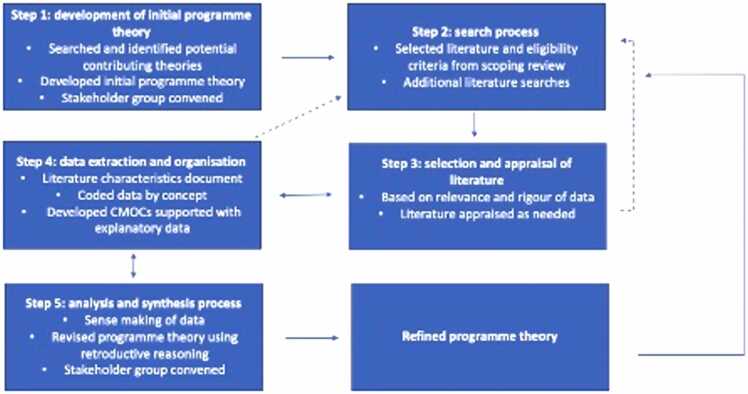
The realist review steps showing the iterative nature of the review process. Dotted lines represent the return to the search process from later steps.

**Fig. 2:**
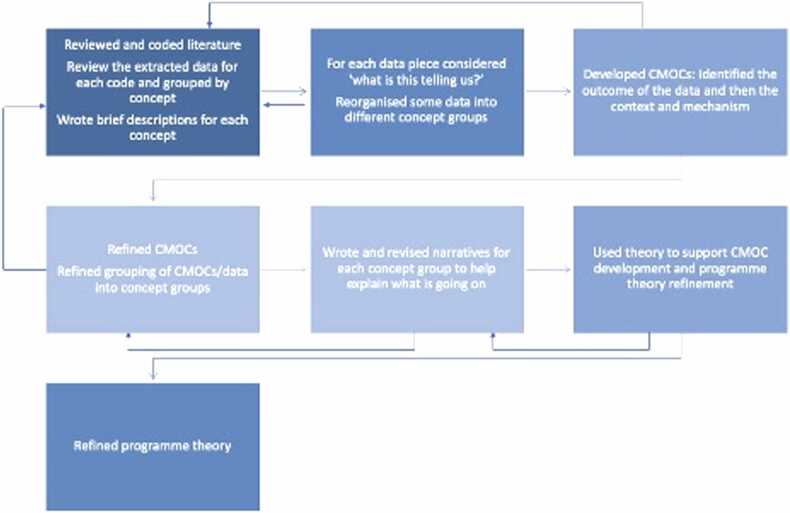
Overview of data extraction and organisation and analysis and synthesis processes.

This realist review, undertaken between October 2021 and October 2022, followed the five steps outlined by Pawson in The Science of Evaluation (2013), summarised in Table 1 and Fig. 1 (Pawson, 2013). It was an iterative process that moved forward and back between the different steps. Table 2 lists the eligibility criteria for literature selection and Fig. 2 provides an overview of the data extraction, organisation, analysis, and synthesis processes. A protocol was developed and registered with PROSPERO CRD42021286452. The results are reported according to the RAMESES publication standards for realist reviews (Wong et al., 2013).

**Table 1:** Detailed steps of the realist review.

Step 1: Development of the initial programme theory	• Elaborated our preliminary thinking about how and why networks develop and work to improve quality of care and service delivery • Consulted the scoping review LMIC literature to draw out relevant insights (Kalaris and Wong, 2023) • Considered substantive theories used in other studies on networks • Recruited stakeholders from global health, academic, and research institutions to provide insight and guidance into the realist review process and our evolving thinking • Stakeholder group met; the initial programme theory and the realist review process were presented and discussed
Step 2: Search Process	• Started with the 127 pieces of selected published and grey literature from our scoping review (Kalaris and Wong, 2023) which systematically searched six databases (Medline, Global Health, Embase, Web of Science, Cochrane Library, Global Index Medicus’ Africa Index Medicus) and purposively search grey literature between 2000 and 2021 on the 3rd and 4th February 2021 (search strategy in Supplementary File 1) • Modified the search strategy to excluded four search terms due to the narrower focus of the realist review (search strategy in Supplementary File 2) • Searched four databases (Medline, Global Health, Embase, Web of Science) using the modified search strategy to identify any more recent literature on the 27thApril 2022 • Searched for substantive theory to identify transferable concepts that might help unlock aspects of the programme theory by: o Reviewed included literature to identify relevant theories from other studies o Suggestions from the co-authors o Snowballing from other theories
Step 3: Selection and appraisal of the literature	• Literature selection was based on relevance to the programme theory and rigour • Narrowed the eligibility criteria from the scoping review (Kalaris and Wong, 2023), presented inTable 2, with additional exclusions as the review became more focused • Reviewed scoping review literature in waves based on its likelihood to contribute relevant information to the programme theory and building of causal explanations • Prioritised LMIC literature • Included limited HIC literature from the scoping review where it provided generic insights to support the causal explanations and where there were not LMIC supporting data but where we judged that we could make justified inferences about the transferability of the causal explanations • Selected substantive theories based on their relevance to the programme theory and where they could provide deeper explanations into the programme theory and help infer mechanisms • Appraised studies with when CMOC had data from only one supporting source: o 15 CMOCs had only one supporting source and 10 sources needed to be appraised o Literature appraisal is available in Supplementary File 3
Step 4: Data extraction and organisation	• Imported literature into NVivo QRS International, version 12, a data management software tool for qualitative analysis • Reviewed and coded literature in waves starting with literature that was most likely to hold the most relevant information to explain different aspects of the programme theory • Coded literature deductively and inductively with concepts that were judged to be relevant to the programme theory • Reviewed data for each code and recoded to more appropriate codes where relevant
Step 5: Analysis and synthesis processes	• Grouped data by concept with a brief description to explain what the group of data was about and how it fits into the programme theory • Considered for each piece of data ‘what is this piece of data telling us?,’ which required some reorganisation of data into different concept groups • Iteratively developed CMOCs for each concept group, starting by identifying from the data the outcome and context and then employing retroductive reasoning to help identify mechanisms (Greenhalgh et al., 2017a) • Continued this process until all the included literature was reviewed • Decided to focus on the first part of the programme theory – on the initiation of a network because: o This part of the programme theory had less data o We judged that the initiation of a network may be more transferable across diverse networks • Continued to refine the CMOCs specifically bringing them up to a higher level of abstraction • Used substantive theories to help infer mechanisms and further explain parts of the programme theory • Continued to iteratively revise the CMOCs, the narratives that provide additional details about each CMOC, and programme theory with substantive theories until theoretical saturation was reached and the initial programme theory was sufficiently refined explanation-wise • Convened a stakeholder group meeting to present and discuss the refined programme theory to support sense-making of the refined programme theory, provide insights into the direction and focus of the programme theory, and provide insights on the direction of a subsequent realist evaluation to test the programme theory

HIC: High-income country, LMIC: low-and-middle-income country, CMOC: Context-Mechanism-Outcome Configuration

**Table 2:** Eligibility criteria. Italics show additions to the exclusion criteria for the realist review.

Inclusion	Exclusion
• Related to health system networks • Networks: groups of facilities and/or healthcare stakeholders (including but not limited to all types of providers, technicians, government officials, professional associations, NGOs, and donors) linked formally or informally, horizontally or vertically, through programs, interventions, activities, or initiatives • Health systems: structures, processes, and people responsible for managing health programmes and services that provide care for a population • HICs and LMICs	• The programme, intervention, activity, or initiative occurs only in one facility or locality among only one group of actors • Research or purely academic networks • Networks focused on research capacity building • Communities of practice • Advocacy networks • Disaster management networks • Database/registry networks • Trial/study networks • Social networks • Family/home care networks • Palliative care networks • Laboratory networks • Diagnostics networks • Disease surveillance networks • Telemedicine/e-health/m-health interventions • Accreditation interventions • Integrated community case management interventions • Integrated management of childhood illnesses interventions • Mobile unit outreach • Faith based organisation sector • General studies on Universal Health Care • Primary Health Care networks that were limited to the peripheral level of the health system • *Quality improvement collaboratives* Quality improvement collaborations, quality improvement programmes, and collaborative improvement networks were added to the exclusion criteria for the same reasons they were excluded from the search strategy as described above. • *Quality improvement programmes* • *Collaborative improvement networks* • *Social franchise networks –* Social franchise networks were excluded because they often have a different focus (marketing health products). In the scoping review there were very few examples of social franchise networks identified and the examples contributed little relevant information • *Telehealth networks* Telehealth networks were excluded as the focus was often specific to only the virtual interaction.

HIC: High-income country, LMIC: low-and-middle-income country, NGO: Non-Governmental Organisation.

## 3 Results

### 3.1 Selected literature

**Fig. 3:**
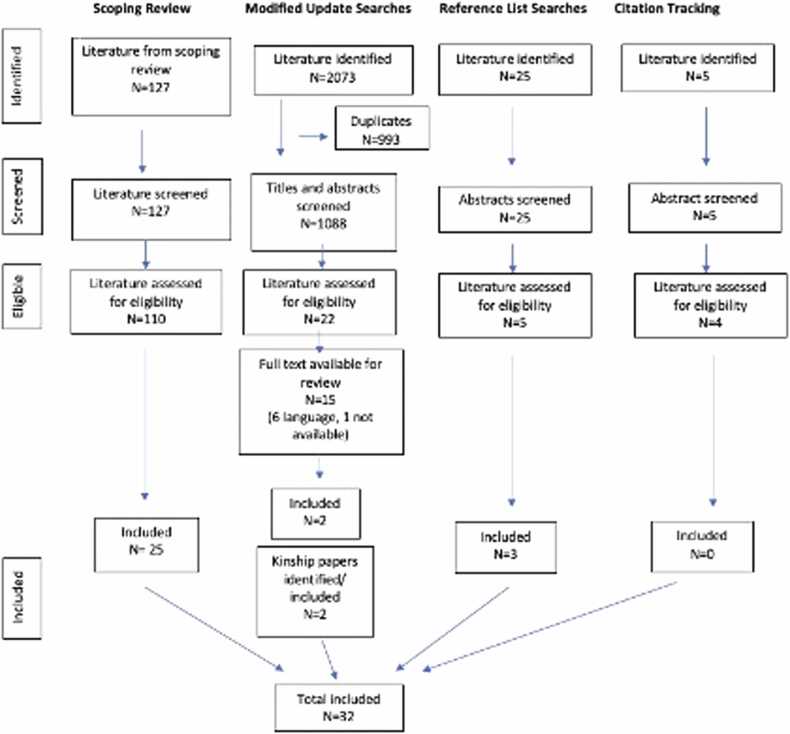
Realise review PRISMA. Evidence selection process from scoping review literature and additional searches.

The evidence selection process is outlined in Fig. 3. 32 pieces of literature were selected from 1245 unique pieces of literature that were identified through the search processes.

Through the iterative search process and the refining of the programme theory the theories and concepts in Table 3 were identified as relevant to support and further explain the programme theory.

**Table 3:** Substantive theories used to refine the programme theory.

Theory/Concept	Key Author	Overview
Theory of collective behaviour	Smesler 1962	• Explanation for the formation of social movements • Social movements start with the experience of a strain, a generalisable belief rises identifying the source of the strain, there needs to be a precipitating event, leadership gives the movement direction, social control mechanisms can affect the direction of the movement
Collective identity approach from New Social Movement Theory	Melucci 1995	• Shared identity is developed through common experiences and emotions and the development of a common perspective or understanding
Leadership in social movements	Ganz 2010	• “accepting responsibility to enable others to achieve shared purpose in the face of uncertainty” • Five leadership practices: relationship building, storytelling, devising strategy, structuring social movements, and catalysing action
Theory of planned behaviour	Ajzen 1985	• When the behaviour is not under self-control, if an individual thinks they have the resources and opportunities to perform the behaviour, they have greater perceived control over the behaviour, while if they lack resources and opportunities, they may have lower intentions to perform the behaviour, even if they have a favourable attitude toward performing the behaviour
Self-efficacy theory	Bandura 1977	• Individual’s belief in their ability to enact behaviours needed to arrive at a certain outcome
Small group development theory	Tuckman 1965	• Model that conceptualises changes in the behaviour of groups • The model has four stages: forming (coming together, developing relationships), storming (resistance to the newly formed group), norming (developing cohesiveness, new standards), and performing (the group is functional and executes tasks)
Organisational culture theory	Schein 1990 Schein, Bennis, Blake 1965	• “set of shared mental assumptions that guide interpretation and action in organisations by defining appropriate behavior for various situations”
Organisational commitment theory	Porter 1974 Meyer, Allen 1991	• “attachment to the organisation, characterised by an intention to remain in it; an identification with the values and goals of the organisation; and a willingness to exert extra effort on its behalf” • Types of commitment: affective, continuance, normative commitment
Collective intelligence theory	Malone, Bernstein 2015 Malone, Woolley 2020	• “groups of individuals acting collectively in ways that seem intelligent” • Explains extrinsic and intrinsic motivations
Psychological safe space	Edmondson 2004	• “individuals’ perceptions about the consequences of interpersonal risks in their work environment” • “a climate in which the focus can be on productive discussion that enables early prevention of problems and accomplishment of shared goals, because people are less likely to focus on self-protection”

### 3.2 Literature characteristics

Complete characteristics of the included literature are found in Supplementary File 4. Of the 32 included pieces of literature, 24 were focused on LMICs, two on middle-income and HICs, and six on HICs. They represented a mix of study designs: nine case studies, six qualitative studies, five programme overviews, four evaluations, two reports, and one observational study, mixed methods study, and realist evaluation. The three pieces of grey literature were all short programme overviews. There are a plethora of different types of networks and it is difficult to neatly categorise them all (Kalaris and Wong, 2023). Of the included studies, there were five on Networks of Care and the Clinical Information Network, three on referral networks and public healthcare networks, and two on primary care networks and clinical networks.

### 3.3 A network programme theory

The initial programme theory contains 58 Context-Mechanism-Outcome Configurations (CMOCs) supported by 185 sections of data from 32 pieces of literature. A complete list of CMOCs and all supporting data are available in Supplementary File 5. The programme theory explains how networks are initiated, formed, and function in a way that sets them up for network members to be able to change practices, which may lead to improved quality of care, service delivery, and clinical outcomes. There is not one clear measure of network success but much of the literature features networks reported to be successful in either its operation or outcomes. Therefore, this review helps to understand how networks could be set-up and function to have a chance at success.

**Fig. 4:**
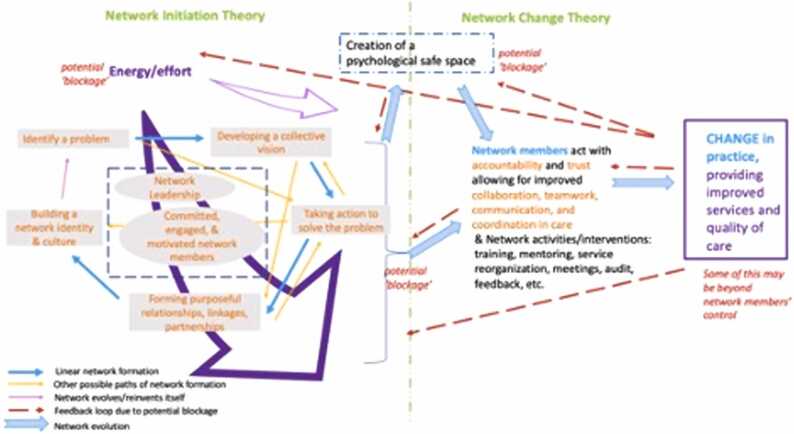
Programme Theory.

The programme theory has two sub-theories – a network initiation theory and a network change theory. This realist review focused on the network initiation theory because it was where we felt there was less existing understanding. If a network is not set-up taking into consideration certain elements it might be less likely to be able to perform and enact its mission. In the network change theory, the network is more susceptible to forces and circumstances outside of the network members and leaderships’ control, potentially hindering the ability to change practices, which was not well elaborated in the literature. Text Box 1 provides the narrative of the programme theory and Fig. 4 the visual representation.

Text Box 1 **Narrative of the programme theory.**

*The formation of a network starts with the identification of a problem by health system actors. This problem, in service delivery or health system functioning, causes a tension among health system actors that triggers an energy to work on improving the identified problem. Due to similar perspectives and experiences and others who feel this same tension, the potential network members coalesce around a collective vision. They realise that they can pool their energy to collectively take action toward solving the problem. Network members will form or strengthen existing purposeful relationships, linkages, and partnerships. These intentional relationships, linkages, and partnerships differentiates the network from the health system. Network leadership emerges early in the formation of the network but evolves as the network matures. Overtime, a network identity and culture begin to emerge. Network members become committed, engaged, and motivated to the network’s collective vision, identity, and culture and the network becomes a psychological safe space.*

*The combination of the network, as a psychological safe space, and the network members as committed, engaged, and motivated enables them to act in a way in which they improve teamwork, communication, coordination, and accountability. This facilitates changes in behaviour that can improve practices in quality of care and service delivery, and ultimately clinical outcomes. However, there are many outside factors that may prevent changes in practices, the provision of care, or outcomes that are beyond the network members’ control.*

Our findings below provide detail of what underpins our programme theory and are grouped into nine different sections. The programme theory is presented in a sequential or temporal way, however in reality, following *Identify a problem* these sections happen in parallel or varying permutations of order.

#### 3.3.1 Identify a problem

Networks often form around a problem in clinical care, service delivery organisation, or health system management. A mismatch between expectations of a norm and reality, either at the individual or organisational level, surfaces the problem. These problems are often identified by clinicians, health systems administrators, and other health system stakeholders, who may have an initial energy to get ‘doing something about it’ off the ground coming from frustration that the problem causes or excitement of potentially doing something about it. The dissatisfactions experienced by health system actors vary, for example, clinicians may feel frustrated due to a lack of resources, which limits their ability to provide high-quality care and undermines their motivation (CMOC 1 A).

Problems are not normally felt in isolation and other health system actors are likely to experience similar strains. As potential network members coalesce around the identified problem, others are drawn into their cause. This is because the idea comes from the potential network members themselves and so there is a willingness or realisation that they can do something about the problem (CMOC 1B). Potential network members have collective experiences and perspectives related to the identified problem and when they share these, drawing on their collective knowledge, they are better able to find a solution (CMOC 1 C). When problems are identified by individuals, networks often form from the bottom-up, while when the problem comes from health systems administrators or outside stakeholders, the network may be triggered through a top-down approach (and may be linked, for example, to Ministry of Health targets). Over time networks formed from the top-down may shift to being driven from the bottom-up and networks developed from the bottom-up may become formalised or integrated.

Once in existence, networks can be employed to identify additional problems and, in this way, may continually reinvent themselves to stay relevant. The identification of problems within a network may occur in clinical practice, through learning sessions and coaching visits, or through approaches that search out to identify barriers or challenges to uptake in care or service use (CMOC 1D). There are insufficient detailed data in the literature explaining how the identification of a problem leads to the initiation of a network and why this process needs to occur, however, looking to relevant substantive theory it is possible to identify concepts, that lend themselves to explaining why the identification of a problem is essential to initiate a network.

Smelser’s (1962) theory of collective behaviour is an underemployed explanation of the formation of social movements (Weeber and Rodeheaver, 2003, Ormrod, 2014). Smelser argues that social movements start when a group of people experience a ‘strain.’ This strain can be considered akin to a problem identified by groups of clinicians, health system administrators, patients/users, or other health system stakeholders. These individuals all feel similarly about the problem and coalesce around it to do something. Smelser argues that following the emergence of the strain, a generalisable belief rises, which identifies the source of the strain and suggests actions to rectify it. In the formation of a network, the generalisable belief comes from frustrations the problem causes or belief that the status quo is not good enough and emerges into a collective desire to solve the identified problem. This generalisable belief may also have roots in a vocational calling or professional identity. Smelser reasons there should be a precipitating event to help form the collective behaviour. In the formation of a network, there may not be one major event, but the repeated occurrence of the same strain or problem causes the clinicians, administrators, stakeholders, or patients/users to arrive at a tipping point. Furthermore, according to Smelser, some form of leadership is needed in all social movements, which is consistent across networks despite different leadership models. Lastly, Smelser’s theory explains that ‘social control mechanisms,’ resulting from the actions of those in power, could have an impact on the direction of the movement. Networks’ ability to change practices may be constrained by outside influences, such as political influences or the ability to get support and resources that could affect network impact and success. The role of leadership in networks and ‘social control mechanisms’ in the absence of non-network committed stakeholders are discussed later.

#### 3.3.2 Developing a collective vision

The development of a collective vision among network members is key to its success. There are three central parts to a collective vision: agreement around the identified problem, the intention to act, and initiating planning for actions to be taken to do something about the problem and further solidify the formation of the network. Network members’ willingness to work towards this collective vision may depend on extrinsic factors, such as policies, organisational practices, and working environment, or intrinsic factors, like personal values, job expectations, and self-esteem (Franco et al., 2002). Extrinsic factors may be more likely to drive network members willingness in networks initiated through top-down approaches. When network members agree to a vision, it can help to prioritise network actions and act as a supportive mechanism to reach the network goals.

Once a problem has been identified, potential network members begin the process of developing a collective vision. This occurs through a process of collective sense-making resulting in the identification of commonalities. It is a process of negotiation and clarification that may happen through interactions among network members, initial network meetings, and guidance from network leadership (CMOC 2 A). Commonalities can be developed through sharing lessons and best practices, open communication, and working together to solve problems, which leads to the development of a collective vision. However, differences in perspectives can be an inhibitor to developing a collective vision and working together (CMOC 2B). Another context that leads to the development of a collective vision is when network members have common professional or vocational identities or callings and so they likely have similar experiences, understanding, and perspectives which helps the development of a collective vision because they understand where other network members are coming from (CMOC 2 C).

Once potential network members have developed a collective vision, they need to put in effort to commit to it and operationalise it. This may happen when network members have a similar perceived reality because the collective vision is based on shared experiences, emotions, perspectives, and understanding (CMOC 2D). Network leadership often has an important role in getting network members to commit to the collective vision. Leadership may employ approaches such as using clear communication, advocacy, and promotion of the collective network vision as well as helping set collective goals and supporting network members (CMOC 2E). Furthermore, networks often use Memorandums of Understanding (MoUs) to solidify the network with purposeful agreements, objectives, and clear roles and responsibilities and as a mechanism to hold network members accountable (CMOC 2 F).

An established collective vision helps potential network members to form collaborative relationships and take coordinated action because they are aligned with a common understanding and perspective of the network (CMOC 2 G). As the network evolves, the initial collective vision may change over time, particularly as established networks identify new problems and reinvent themselves. If a network does not have a collective vision or if network members do not follow it, then the network may be less functional. A collective vision may not form if the network is dominated by individual interests or if there is a divergence of beliefs. If network members do not adhere to the collective vision, there may be resistance to network activities (CMOC 2 H).

Smelser’s theory of collective behaviour (see above) argues the need for a ‘generalisable belief’ to form a social movement, (Weeber and Rodeheaver, 2003) which can be considered analogous to the development of a collective vision focused around the problem the network aims to solve. The second theory that supports the development of a network’s collective vision is collective identity approach from New Social Movement Theory. This theory posits that a shared identity is developed through common experiences and emotions and the development of a common perspective or understanding (Engles and Muller, 2019, Fominaya, 2010, Polletta and Jasper, 2001). A collective identity is the basis from which a network’s collective vision is formed to target the problem the network aims to address (i.e. the generalisable belief). The collective vision emerges from the identified problem because potential network members have common experiences, perspectives, and understanding around the problem; this means that the vision will be collective and grounded in a common perspective or understanding.

Tuckman’s (1965) small group development theory is a model that conceptualises changes in the behaviour of groups. It has four stages: forming (coming together, developing relationships), storming (resistance to the newly formed group), norming (developing cohesiveness, new standards), and performing (the group is functional and executes tasks) (Tuckman, 1965, Tuckman and Jensen, 1977). The norming phase lends itself to explain how network members progress from developing a collective vision to the vision becoming a norm and network member internalisation and adherence to the collective vision.

Network members must put in ‘work’ to develop, internalise, and adhere to the collective vision. Tuckman’s theory explains some of this ‘work’ behind the creation of group norms, which includes acceptance of other group members, developing a sense of being as a group, identification with the group, agreed upon group action, cooperation, mutual support, having a collective mindset or feeling, and having a common goal and group spirit (Tuckman, 1965). This ‘work’ also resonates with aspects of the collective identity approach, such as identifying and articulating commonalities among each other, collective sense-making, and having common perspective and understanding (Engles and Muller, 2019, Fominaya, 2010, Polletta and Jasper, 2001). There is overlap with the collective identity approach in articulating some of this work that needs to be done. When the collective vision is a norm of the network, it can help lead to changes in practices.

#### 3.3.3 Taking action to solve a problem

Once potential network members have identified a problem and started to form a collective vision, they reach a tipping point in collective will to collectively take action to try to resolve the problem. This distinguishes doing something about the problem from not being able to get initiatives off the ground.

When potential network stakeholders realise that collective action is needed to solve the identified problem, they will seek out those with common experiences. Potential network stakeholders may come from different geographies and professions but the realised need for collective action towards the identified problem transcends these differences (CMOC 3 C). In a network formed from the bottom-up potential network members will recruit likeminded colleagues to take action to solve the problem because with collective action, they will be more likely to do something related to the identified problem. The recruitment might occur through communicating and collective sense-making around the problem or a realisation on the part of potential network members that working together positively supports the ability to achieve a shared goal. While, in a top-down network, a sense that collective action is still needed will come from a health system administrator or manager who may mandate colleagues to participate in the network (CMOCs 3 A, 3B). When network members have clearly identified what and how a problem needs to be solved, opportunities to share and work together as a network may facilitate their ability to take action. This helps with problem-solving and identifying new solutions (CMOC 3D).

While the literature is sparse in this area of the programme theory, substantive theories provide initial insight into understanding how potential network members take action. Drawing on the collective identity approach from New Social Movement Theory, described above, as with social movements, network members share common experiences and develop a common perspective or understanding through working in the same health system, geography, or technical area. When network members have a collective identity, it enables a sense of collective agency. The existence of collective agency may explain how groups of individuals as part of a network act, instead of continuing with the status quo.

Ajzen’s (1985) theory of planned behaviour extends the theory of reasoned action (Fishbein, Ajzen 1975 (Fishbein and Ajzen, 1975)) which argues that when a desired behaviour is under one’s own control an individual’s behavioural intentions are based on their belief in the likelihood that performing a specific behaviour will lead to a certain outcome. When the behaviour is not under self-control, the theory of planned behaviour posits that if an individual thinks they have the resources and opportunities to perform the behaviour, they have greater perceived control over the behaviour, while if they lack resources and opportunities, they may have lower intentions to perform the behaviour, even if they have a favourable attitude toward performing the behaviour (Madden et al., 1992). This links with Bandura’s (1977) self-efficacy theory, regarding individual’s belief in their ability to enact behaviours is needed to arrive at a certain outcome. While Bandura’s theory is focused on the individual, it can be postulated that when individuals are part of a collective, this helps the individuals believe they can be effective together and so group-efficacy could be achievable at a lower threshold than on an individual level. These theories help to explain that if network members believe that collectively they have the ability and resources, such as a collective identity, to solve the identified problem, they are more likely to do so. This moves potential network members from identifying a problem with the intention to do something about it to taking action towards solving the problem.

#### 3.3.4 Developing purposeful relationships, partnership, and linkages

Purposeful relationships are at the heart of a network; in health systems the relationships between people are not always purposeful or strong but in a well-functioning network, health system relationships and linkages are more likely to be. Networks thus potentially offer a solution to an important health systems problem: “Health systems are inherently relational and so many of the most critical challenges for health systems are relationship problems.” (Gilson, 2003) The relationships reinforced and built while forming a network, particularly one from the bottom-up, can support improvements in service delivery (Fasawe et al., 2020). Relationships, linkages, and partnerships in the network are grounded in the network’s collective vision.

This next phase of the programme theory begins with understanding how networks develop purposeful relationships, linkages, and partnerships. The purposefulness with which these relationships are formed, differentiates a network from standard health system or programme interaction. When relationships, linkages, and partnerships are formed with purpose around an aim, they can support the health system to provide care and services in a way that it might not be able to otherwise. For network members to ensure that they have necessary and functional relationships, they need to adhere to and believe in the network’s collective vision and be open to and have the time to invest in developing relationships (CMOCs 4 A, 4B). The existence of prior relationships may be useful to develop deeper network relationships because network members are familiar with each other, but it is not a pre-requisite (CMOC 4 C). However, there are examples of how a lack of pre-existing relationships was a hinderance to network functioning. The existence of artefacts, such as documented MoUs, roles and responsibilities, and procedures and processes, may help network members create and sustain the right kind of relationships (CMOC 4D). While some relationships may exist prior to the development of the network, the network creates opportunities to strengthen these existing relationships and establish new ones through, for example, trainings with network members from different cadres or locations (CMOC 4E).

Purposeful relationships, linkages, and partnerships developed through the network can help to improve communication between network members (CMOC 4 F). When relationships are created on mutual understanding, they can help to bridge gaps between network members as well as between network members and external stakeholders. Mutual understanding helps to bring them together and overcome differences. Bridging gaps between network members can help them become engaged and motivated and part of a collective identity. It is important for the network to have productive relationships with external stakeholders because they could act, as Smelser referenced as a ‘social control mechanism,’ and inhibit the development of the network or implementation of network activities (CMOC 4 G).

Tuckman’s small group development theory stage of forming helps explain the process of network members coming together and developing initial network relationships. As network members orient themselves with other members and the network, they establish new ties between each other and with the network leadership; this signifies a shift from potential network members to a network group with shared social capital. New Social Movement Theory’s collective identity approach (Polletta and Jasper, 2001) and organisational culture theory (Schein, 1990, Allaire and Firsirotu, 1984, Ravasi and Schultz, 2006) argue that pre-existing relationships are important to form a collective identity and culture. This lends itself to forming successful network relationships, however successful relationships can be formed without pre-existing relationships through the process of forming as described by Tuckman.

#### 3.3.5 Network leadership

Leadership is an essential part of the network and can “help overcome some of the ‘wicked problems’ inherent in achieving changes at scale, particularly if they can effectively engage sets of practice leaders who together embrace the change efforts.” (Akech et al., 2019) Network leaders are often described as needing to be “visionary, strategic, and trusted” (McInnes et al., 2012) and have soft skills (Irimu et al., 2018). According to Tuckman’s small group development theory, leadership emerges during the forming stage when a group may have an initial dependence on the leader (Tuckman, 1965).

While there is not one standard leadership model across networks, for the purposes of this programme theory, it is important to understand what leadership does within a network to set it up and keep it going, what they do to make sure that the network achieves what it was set-up to, how networks cultivate and grow leadership, and leadership challenges or blockages to network initiation and functioning. Furthermore, network leadership has an essential role to play in supporting the establishment of a psychological safe space, which is discussed in a later part of the programme theory.

Network leadership plays an important role in setting-up a network and supporting its ongoing functioning. They have an operational and organisational role to play, including project management, coordination, convening meetings, and implementing workplans. Network leadership builds linkages and relationships within networks and between the network and external stakeholders. They have a central role in both formal and informal communication internal and external to the network (CMOC 5 A). Network leaders are responsible for communicating the network vision and principles as well as act as knowledge brokers (CMOC 5B). Leadership also plays a supportive role towards network members, by for example providing feedback and supporting collaboration (CMOCs 5 C, 5D).

Network leaders play an important role in the implementation of network tasks and activities through network coordination, dedicating time to the network, and showing that they can get things done. This can engage and motivate network members because it meets a need to feel that they are involved in something positive (CMOC 5E). When network leaders dedicate time to support setting-up and the ongoing functioning of the network this may support network members to become more committed, engaged, and or motivated (CMOC 5 F). When network leadership actively engages network members in network activities this can support a network to become more successful (CMOC 5 G).

Network leadership facilitates members to achieve what the network was set-up to do by providing support, influencing, championing, promoting, and encouraging practices, mentoring and providing feedback, and creating a positive environment that makes members feel part of a network and allows them to fulfil a latent desire to practice better (CMOCs 5 H, 5I). The literature indicates that there are different activities and behaviors that leadership can do to create a welcoming and inclusive environment for network members, for example senior clinicians checking-in with providers about patient care and offering help, providing encouragement and support, and recognising network members (CMOC 5 J).

It is also important that as networks grow, they cultivate leadership. When networks initially form, they often have dynamic and engaging leadership. Overtime, the network will need to cultivate leadership throughout the network to support its ongoing functioning. In some networks, having distributed leadership may be important for optimal network functioning and successes. Networks can be a mechanism to develop leadership at different levels of the health system (CMOC 5 K).

Leadership can present challenges to network initiation and functioning. When a network relies too much on the presence or vision of one network leader, this can hinder a network’s development, functioning, and sustainability (CMOC 5 L). An important role of a network leader is to get network members to develop and agree upon a shared vision, if they are not able to do this, then the network might not function optimally, and network members might not be engaged (CMOC 5 M).

The literature on social movement leadership is an underdeveloped area of social movement theory. Historically, social movement leadership has been conceptualised as charismatic and bureaucratic authority and there is often a lack of differentiation between authority structures, leaders, and movement members interactions in the exercise of leadership (Ganz and McKenna, 2019). Smelser’s theory of collective behaviour states that social movements need engaged leaders to provide the movement with a sense of direction (Ormrod, 2014). Ganz (2010) suggests that social movement leadership can be understood as “accepting responsibility to enable others to achieve shared purpose in the face of uncertainty.” This is enacted through five leadership practices: relationship building, storytelling, devising strategy, structuring social movements, and catalysing action, (Ganz, 2010) which resonate with aspects of leadership in networks. For example, network leaders have an important role is facilitating relationship building in the network and communicating the network vision (storytelling). Network leaders are central in the organisation and operation of networks, which can be considered akin to structuring social movements, and motivating network members to take action (catalysing action). Ganz also highlights the importance of leadership development in social movements, (Ganz, 2010) the cultivation of leadership throughout a network is essential to support its ongoing functioning.

#### 3.3.6 Building a network identity and culture

A network develops an identity and culture throughout the process of network initiation, and it continues to evolve as the network shapes itself through working to solve the identified problem. The existence of a network identity and culture may facilitate commitment to the network.

This part of the programme theory looks at how network members develop a network identity and culture. As network members coalesce around a collective vision to solve the identified problem, a distinct network identity and culture emerges. Some of this is driven by their collective identity, which comes from common experiences and emotions and developed common perspective or understanding, but the network also needs to create opportunities to enable the identity and culture to grow. A network’s culture and identity are not static, and they will evolve over time as the network matures and attempts to tackle different problems (CMOC 6 A). Network members’ identification with a network can be a motivating factor for wanting to belong (CMOC 6B). A network identity can form when network members identify with each other and the network vision (CMOC 6 C). As a network establishes itself, it begins to create a network culture. This culture is based on the network identity developed among network members when they connect, share experiences, and learn from each other in an open and safe environment (CMOC 6D).

A network culture can replace existing ‘negative’ cultures. Pre-existing cultures in health facilities or organisations that network members are also part of may not align with the network’s collective vision to solve the identified problem. Existing cultures may in fact be a cause of the identified problem. When network members are motivated by the collective vision, the network might need to shift existing cultures in the network to create a network culture that is in line with the collective vision (CMOC 6E).

The development of a network identity and culture starts with a sense of collective identity. A shared identity emerges between network members from common experiences, perspectives, or understandings. As described above, a collective identity facilitates the development of a collective vision and may help to explain why people chose to participate in a network – particularly if there are no clear extrinsic motivations. According to New Social Movement Theory’s collective identity approach, people join social movements because they believe in the movement’s aims but also because they identify with the movement – it becomes part of their individual identity, and with time the movement develops its own collective identity (Engles and Muller, 2019, Fominaya, 2010, Polletta and Jasper, 2001). The same could be said for networks. A network forms a new identity that is specific to and distinguishes the network from other groups that network members may be affiliated with. The network identity does not replace existing identities of network members but adds a new layer of a collective professional identity. It is from this network identity that the culture of the network grows.

It takes time and deliberate effort on the part of network members and leadership to form an organisational culture because there needs to be stability and common history among members. An organisational culture can be described as a “set of shared mental assumptions that guide interpretation and action in organizations by defining appropriate behavior for various situations.” (Ravasi and Schultz, 2006) It helps members make sense of and give sense to what the organisation is about. A network culture develops over time as members solve both internal and external problems. Pre-existing relationships can help develop a network culture. If a network lacks a culture, members might not want to remain.

Tuckman’s small group development theory’s stages of forming (orientation and development of relationships) and norming (developing cohesiveness, new standards) also support explaining network identity and culture development (Tuckman, 1965). The process of network members coming together and developing relationships, i.e. forming, is necessary for them to develop a specific identity and culture. This leads into the norming phase and network members feel a sense of belonging to the network, facilitated by its collective identity and culture. The network identity and culture are tangible manifestations of the collective vision that can be vehicles for network recruitment and to spread the network’s norms.

#### 3.3.7 Commitment

Commitment to the network is necessary for the network to be able to do things to solve the identified problem and ensure the sustainability of network gains and impact. For a network to function and enact the collective vision, commitment is needed from network members and often from outside stakeholders as well. The network’s collective vision, identity, and culture may facilitate commitment to the network. A network’s leader may play a role in gaining commitment from network members.

This phase of the programme theory starts with understanding how to harness commitment from network members. When networks form from the bottom-up, the support from leadership and the co-creation of a network vision and goals exemplify how to get commitment from network members. The existence of shared experiences and perspectives among network members may also facilitate their commitment because the network’s collective vision, identity, and culture has grown out of these previous shared experiences and perspectives. When network members see the network’s value and use, especially if it aligns with their own concerns or views on change, they may increase their commitment to the network. Network members in top-down formed networks may also identify with the network’s collective vision, identity, and culture due to shared experiences and perspectives (CMOC 7 A). When networks are formed from the top-down, institutional support may help to ensure commitment of time and resources to implement network activities and the participation of network members (CMOC 7B).

Committed network members are more likely to take action on the identified problem and adhere to and enact the collective vision. Examples of the success that networks have with committed members include improved coordination, communication, and data use for clinical care (CMOC 7 C).

Health system stakeholders not part of the network may play an important role in the ability of a network to develop, function, and achieve successes. When these stakeholders are committed to the network it makes it easier for the network to form and function and they are less likely to exert the ‘social control mechanisms’ described by Smelser that constrain the direction or growth of a movement (Weeber and Rodeheaver, 2003, Ormrod, 2014).

Networks employ various techniques to gain commitment to the network from non-network stakeholders, for example, establishing MoUs, incorporating network activities in government policies and plans to mobilise local funding, advocacy by network leadership, meetings, and information sharing. Additionally, prior relationships that fostered trust between network members and outside stakeholders can generate network commitment from outside stakeholders. If outside stakeholders are not committed to the network, this can have a negative effective on network development and functioning (CMOC 7D).

When outside network stakeholders are committed to the network, they are more likely to support its functioning and the sustainability of network initiatives, sometimes extending them to additional geographies or leveraging additional resources. For example, local governments recruit and contract healthcare workers and invest in infrastructure, drugs, and materials for facilities in the network. They may also take ownership of network initiatives, for example a data collection system that can be expanded beyond the geographic boundaries of the network (CMOC 7E).

Organisational commitment is defined as the “strength of an individual’s identification with and involvement in a particular organisation” and is characterised by belief and acceptance of the values and goals of the organisation, willingness to expend effort on the organisation’s behalf, and a desire to stay with the organisation (Porter et al., 1974, Meyer and Allen, 1991). If a network has a collective vision, network members are more likely to be aligned and committed to the network. A network approach may be a way to improve larger organisational commitment because it brings value and purpose based on the collective vision to what might otherwise be a sub-optimally functioning system. However, there could be potential unintended consequences of less diversity of opinion if the network only brings in people with similar experiences, perspectives, and understandings, though those with similar experiences could still have different perspective and understandings.

Organisational commitment theory posits that various factors affect commitment to an organisation. Factors relevant to a network include the environment, positive and purposeful relationships and connections, and structure. Networks can be a means by which to recognise network members, which may increase their motivation and subsequently augment their commitment to the network. Previous engagement among stakeholders may support ownership of network activities (Irimu et al., 2018) as well as strong and engaged network leadership.

Organisational culture theory argues that an organisational culture creates a sense of belonging to the organisation, which may be a reason why people decide to commit to the network. When there is an alignment between the network culture and personal values and identities, this may also facilitate commitment and network functioning (Lundmark et al., 2021).

Collective commitment might also be formed during Tuckman’s stages of ‘forming’ and ‘storming.’ (Tuckman, 1965, Tuckman and Jensen, 1977) When network members come together to work out how they are going to work together, this can be a basis for garnering collective commitment. Network members that participate in these stages of network development might feel more or have easier network commitment than if they join at a later stage.

#### 3.3.8 Engaged and motivated network members

Engaged and motivated network members are key to ensure network functioning and that the network meets its goals. A network does not necessarily need financing to run but without active network members who dedicate time to the network, it will not get off the ground.

Networks create various opportunities for network members to become engaged and motivated. For example, network meetings provide opportunities to discuss audit reports, collectively identify problems and solutions, and build soft skills. Clinical improvement and learning opportunities, such as mentoring, professional development, clinical education, and learning from other network members, also help to develop motivated and engaged network members. Financial incentives as well as non-material incentives, like the recognition of good performance, can be motivational factors. When network leaders improve management practices, are in boundary spanning roles, or network leadership is rotational, this can bring legitimacy and value to the network, both encourage participation and engagement.

Network members may be engaged and motivated to be part of the network when they feel like they are getting something out of it, demonstrating continuance commitment. However, if the network does not provide extrinsic motivations, network members are less likely to be committed to the network and its activities. When network members feel an emotional connection to the network or their personal identity aligns with the network’s identity and culture, they are likely to be committed to the network, exhibiting affective commitment (CMOCs 8 A – 8 C).

If network member’s professional values or normative beliefs align with a network’s vision, identity, or culture, for example a vocational or professional calling or peer group norms, they will exhibit normative commitment to the network. The existence of a shared goal can engage and motivate network members, bringing out their normative commitment to the network. However, if there are not the resources available to enable network members to enact change practices, they cannot enact their vocational calling and this can demotivate them. Or, if network members feel they cannot live out their normative attachment because they do not feel they have the power to change practices, this can be demotivating and weaken commitment (CMOC 8D).

When networks can show its members that it can affect change, this will motivate members to be part of the network because they can see results and the value of what they are committing to (CMOC 8E). When network members are engaged and motivated participants in a network, this can support the implementation of activities to bring about change (CMOC 8 F).

Network members’ commitment to a network will be an indication of the extent of their motivation and engagement. Organisational culture plays a role in getting and keeping network members engaged and motivated. Organisational commitment theory explains three types of commitment: affective, continuance, and normative (Manetje, 2009). Network members who have affective commitment will have stronger engagement and motivation to participate in the network because there is an alignment of goals and values, which can be extended to the network vision, identity, and culture, and a desire on the part of the individual to be part of the network. A network culture can generate a sense of belonging to the network and be a reason why network members are committed to the network.

Some network members may take part because of a moral obligation, this may stem from a professional or vocational calling; having normative commitment, they will also be engaged and motivated. If network members feel normative commitment to the network, they might feel morally obliged to commit to the culture. However, if the source of normative commitment is a peer group norm, it may be hard for individual network members to reject the peer group norm and not commit to the network.

Affective and normative commitment will create engagement with the network and motivation to be actively part of it and work towards the collective vision. Since networks have a collective identity, network members may be motivated to participate even without material incentives or coercion – the identity itself is an appealing incentive, according to the collective identity approach from New Social Movement Theory (Fominaya, 2010, Polletta and Jasper, 2001). However, a network member who has a continuance type of commitment to the network may be engaged or motivated because they are getting something from the network but will not be committed in the face of what they feel is a better option.

Collective intelligence theory (Malone and Woolley, 2020) may explain the extrinsic and intrinsic motivations of network members. When network members are affectively or normatively committed to the network, they are more likely to have intrinsic motivations for participating in the network. If there is a network culture that network members feel like they belong to, either because it aligns with their beliefs or they feel a moral obligation to it, then they will be affectively and normatively committed to the network and therefore be intrinsically motivated. If network members are intrinsically motivated, they may be more likely to stay with the network and more positively contribute to its formation and functioning. While if network members have continuance commitment to the network, they are more likely to be extrinsically motivated and may leave, if they find something that better motivates them either intrinsically or extrinsically; they have the weakest commitment to the network.

#### 3.3.9 Creating a psychological safe space

Psychological safety describes “individuals’ perceptions about the consequences of interpersonal risks in their work environment.” (Edmondson, 2004) A psychological safe space has “a climate in which the focus can be on productive discussion that enables early prevention of problems and accomplishment of shared goals, because people are less likely to focus on self-protection.” (Edmondson, 2004) It promotes team learning, improves team performance, dictates how valued and comfortable team members feel, and facilitates collaborative work, particularly navigating uncertainty and change (Edmondson, 2004). When a member of a group feels psychologically safe they feel they can take action without fear of negative consequences. It can also lessen hierarchy.

Through the process of developing the network, a psychological safe space can be created. Network leadership plays an important role in enabling the network to create a psychological safe space. For example, network leaders can act in a way that sets a positive example and encourages network members to feel psychologically safe and can help to cultivate an equal and non-hierarchical network environment (CMOC 9 A). Purposeful non-hierarchical relationships developed by network members are another foundation for network members to feel psychologically safe (CMOC 9B).

When network members have worked to develop a collective vision, identity, and culture, this creates a launching ground for the emergence of a psychological safe space. A network as a psychological safe space facilitates its capability to change practices. Network members may be better able to improve the organisation of service delivery, ensure the presence of necessary infrastructure, provide effective mentorship and supervision, or feel empowered to provide high-quality care (CMOC 9 C). When a network has created a psychological safe space, network members will be able to raise concerns or problems without fear of negative repercussion, will feel more comfortable being innovative, and will be better able to collaborate across the network. This is important because it can help network members change practices (CMOCs 9D-9 F).

Edmondson (2004) describes several antecedents that can promote and influence the development of psychological safety: leadership, trusting and respectful horizontal relationships, opportunities to practice as a team, supportive organisational environment, and informal group dynamics. Psychological safety is more likely to be created in a network that has formed from the bottom-up because there is more natural commitment to the collective vision and likely less institutionalised hierarchy or imposed leadership. When a group has psychological safety it impacts learning, improvement, and feedback-seeking behaviours, enables members to raise concerns or problems without fear of reprisal or embarrassment, encourages innovative behaviour and innovation, and fosters boundary spanning (Edmondson, 2004).

Psychological safety is important in the work environment in order for employees to feel secure and be able to change their behaviour (Schein et al., 1965). A network aims to create a psychological safe space with its engaged and motivated network members who are committed to a collective vision. The identity and culture developed by network members can facilitate the development of a psychological safe space because network members already have common ground and understanding. When network members feel psychologically safe, they are more equipped to work towards changing practices. If a network is not a psychological safe space, then it will be less likely to solve the problem it set out to achieve.

## 4 Discussion

### 4.1 Summary of findings

This realist review developed and refined a programme theory, presented in Text Box 1 and Fig. 4, that provides an explanation of how and why networks are initiated, form, and function in a way that sets them up to be able to change practices. The programme theory is supported by 58 CMOCs and 10 substantive theories to further understanding of the underlying mechanisms and interactions of context that produce the observed outcomes – steps to initiate a network and set it up in a way that network members can change practices. While the findings are presented in a linear way, in practice the phases of the programme theory may happen in parallel, overlapping, or in different orders.

### 4.2 Strengths, limitations, and future research directions

The amount of rich data that has been identified to support the CMOCs and refine the programme theory is a strength of this review, suggesting that the explanatory insights are supported by relatively strong evidence. Additionally, the use of several substantive theories helped to support retroductive reasoning to infer mechanisms where needed. The formation of a stakeholder group to provide insights at different phases of this review also increased its robustness.

This review had several limitations. Compared to the breath of literature on health system networks identified in the scoping review (Kalaris and Wong, 2023), we included a relatively small proportion of that literature. This is because much of the literature reports on the clinical outcomes the network produced and not the details of how and why network members did things. Therefore, there may have been useful examples of networks that were excluded because of the dearth of information that was reported on their formation and functioning.

There are 16 CMOCs for which we have less confidence in the strength of the knowledge claim. For CMOC 2D supporting data was not identified during the search processes, however, the CMOC makes logical sense in the programme theory and the progression of the explanations. There are 15 CMOCs that have only one supporting source of evidence. For these CMOCs an appraisal (see Supplementary File 3) was undertaken to assess the credibility of the methods used to generate the data, thus helping to inform the confidence that we might have in these CMOCs (Wong, 2010). The outcome of the appraisals did not necessitate any qualifications on the relevance of these CMOCs. However, there are limitations with appraisal: the subjective nature of quality, the existence of multiple tools for the same type of study, and the dependence on the detail of the reported information for appraisal. Furthermore, there was one source – a reflective piece for which an appraisal tool does not exist (Irimu et al., 2018).

Relevant HIC literature was included to support LMIC literature and to fill in gaps where there was a dearth of evidence from LMICs. There are six CMOCs supported by only HIC literature. There are a number of limitations related to the selected HIC literature – first, HIC literature from the scoping review was included because it had been inadvertently returned during that search process or we were already aware of the literature and believed it to be relevant to LMICs. As the HIC literature was not systematically searched, there may be relevant HIC literature to the programme theory that has been missed. Secondly, realists argue that mechanisms may be transferable because ‘people are people’ and while the relevant mechanism for some CMOCs were inferred from the HIC literature, the contexts that trigger the mechanisms may be different in LMICs (Greenhalgh et al., 2017b). The strength of the knowledge claim for these CMOCs is therefore less certain because of the lack of LMIC supporting data needed to better understand these mechanisms. Furthermore, the substantive theories are drawn mostly from empirical research and sociological thinking in HICs. As these theories are used to further explain CMOCs developed mainly from LMIC literature, there is a possibility that these theories may have identified mechanisms that are not relevant to people in LMIC settings, this is a persistent problem with theories and why a realist evaluation was planned to collect primary data in a LMIC to test and refine our programme theory (Gilmore, 2019). The fact that there are a subset of CMOCs with limited evidence, suggests that there are areas where further primary research on networks is needed, employing approaches that bring explanations of how and why networks form and work in the ways that they do.

Despite the inclusion of literature from HICs, three-quarters of the literature was from LMICs and therefore, it is reasonable to assume that our findings are relevant to LMICs. Mechanisms, as conceptualised in realist research are assumed to be widely occurring causal forces and hence may be found to operate in HIC as well as LMIC settings and visa-versa (Duddy and Wong, 2023). A planned realist evaluation will test the programme theory with primary data collection, and we will be able to better understand, particularly for CMOCs without LMIC evidence, if these mechanisms are as assumed operating more widely.

The second part of the programme theory, the network change theory, was not investigated or refined to the same extent as the network initiation theory. This was due to the size and complexity of the programme theory, the need to focus to achieve a greater depth of analysis, and its focus on topics where there is more existing research, for example teamwork and communication. This part of the programme theory is more subject to the ‘social control mechanism’ explained in Smelser’s theory of collective behaviour, described above, and therefore may be less under the influence of the network members. However, there is still work to be done on this section of the programme theory within the contexts of health system networks in LMICs. For example, trying to better understand the ‘social control mechanisms’ that may influence the network and the role that power plays in networks. The role of power may be particularly relevant for networks with regards to network leadership, the forming of relationships, and the creation of a psychological safe space.

Examples of networks in the literature are mostly successful examples and therefore a limitation of this review is that there is insufficient information on when things do not work. Only four parts of the programme theory had negative CMOCs (developing a collective vision, leadership, commitment, and engaged and motivated network members). So, while networks may be a useful approach, there may be challenges and unintended consequences. As networks with challenges may fail and are likely not reported, we do not know what caused them to fail and therefore cannot take those things into consideration when forming networks. One potential unintended consequence of networks is that they may encourage groupthink (Blacklock et al., 2022; Mascia and Cicchetti, 2011, Janis, 1972). Networks need buy-in and commitment to the collective vision, identity, and culture and this often comes from having similar experiences and perspectives. Those with different perspectives may be deterred from participating in the network or within a network may be hesitant to provide opposing ideas. This could lead to network members being reluctant to accept new ideas and change and subsequently potentially limit a network’s ability to evolve, be resilient, and achieve its goals.

Additionally, because we were drawing from network examples in different countries to develop the programme theory, we were not able to take into consideration specific socioeconomic, cultural, and political factors for the different settings. As the review was focused on the relationships and interactions between network members that lead to the formation and functioning of a network, we only considered meso and macro level factors when they were relevant to the causal explanations within our programme theory, as other reviews have (Borghi et al., 2018, Topp et al., 2018). However, socioeconomic, cultural, and political factors, among others, may play an important role in network formation and functioning and the planned realist evaluation in one country, may provide an opportunity to consider these factors in the programme theory.

### 4.3 Comparison with the existing literature

There are few studies employing a realist approach to understand a network. Aunger et al. (2022) undertook a realist review and evaluation of inter-organisational collaborations, which included networks between different organisations as well as mergers, strategic alliances, joint ventures, and buddying collaborations. While this review included but was not specific to healthcare and focused mainly on UK examples, the authors touch on some similar themes, such as trust, leadership, culture, stakeholder involvement, information exchange, and communication. Their programme theory’s main focus was on linkages of trust to risk tolerance and faith as drivers of collaborative behaviour for the functioning of organisational collaborations (Aunger et al., 2021a, Aunger et al., 2021b, Aunger et al., 2022). In contrast our programme theory considers a detailed progression of what is needed for network initiation and functioning.

Our findings resonate with the literature on network effectiveness which also points to the need for a network to have a shared identity that starts with the identification of a common problem. Network effectiveness literature posits that those involved in a network feel part of a community, which is similar to the network relationship and psychological safe space phases of our programme theory. Thirdly, they point to the emergence of a solution and mission, akin to the development of a collective vision related to the identified problem part of our programme theory (Provan et al., 2011).

The programme theory also resonates with findings from networks beyond the healthcare literature, particularly education and public administration. Russell et al. (2017) studied the evolution of network improvement communities in education. These networks were also focused on a common goal or aim linked to an identified problem, how it was produced, and how it should be improved and were intentionally designed to bring stakeholders together. Russell et al. developed a framework to guide the development of these networks which included “leading, organising, and operating the network” and “fostering the emergence of culture, norms, and identity consistent with network aims.” (Russell et al., 2017) The authors found that “leadership in networks is rooted in the ability to foster commitment to a common vision and motivate others to engage with it.” (Russell et al., 2017) Leadership, commitment, a common vision, motivation, culture, and identity are all key parts of our programme theory.

Findings from public administration networks touch on similar concepts as our programme theory. These networks also form because there is a problem with the status quo. Building relationships, trust, norms, and commitment are found to be central to the development of these networks (Mandell and Keast, 2008). The idea that network members realise they need to take collective action to solve the problem is also present (Keast et al., 2004). It is possible that our programme theory can provide relevant insights for networks in other fields.

The theories we drew from were mainly from the fields of sociology and psychology. This is because the CMOCs focus on the relationships and interactions of network members at the microlevel and how this supports network formation and functioning. We felt that the theories we selected provided relevant explanatory value for understanding network formation and functioning. However, we could have also drawn from existing network theories, such as social network theory. Concepts from social network theory could be relevant for our programme theory, particularly homophily when looking at group formation (Gamper, 2022). Social network theory may be more pertinent for comparing networks or a deep-dive into a network functioning and performance, which could be an area for future work.

### 4.4 Implications for policy and practice

The findings from this realist review provide a greater understanding of the processes involved in network formation and functioning that enable changes in practices in networks in LMIC health systems. This can lead to a more considered planning and implementation of networks, thereby improving health system functioning and performance. While, the programme theory will be confirmed, refined, or refuted in a future realist evaluation, some provisional recommendations are provided in Text Box 2.

Text Box 2 **Provisional recommendations.**

1. *Forming a network takes time and effort*
2. *The development and buy-in to a collective vision is important and plays a key role as the network evolves*
3. *Purposeful and intentional relationships are key in network initiation and differentiate a network from the health system*
4. *A psychological safe space may be a key facilitator to network members being able to change practices*
5. *External forces can limit the effectiveness of networks*

## 5 Conclusion

The programme theory offers an explanation for how networks form and develop in a way that sets them up to be able to change practices. Each phase of the programme theory indicates something that the network must go through to create committed, motivated, and engaged network members and leadership working in a psychological safe space. Network members are then able to change practices to improve quality of care and services. This programme theory provides an explanation for how many clinical and programmatic networks in LMICs form and work and why they work in these ways. The programme theory draws on the substantive theories from social movement theory and organisational studies to provide greater depth to the causal explanations. While there is limited realist research on networks, one identified realist review on inter-organisational collaboration had resonating themes. Beyond the healthcare literature, several phases of the programme theory resonated with educational and public administration networks, implying that our findings may be relevant to other disciplines.

## Funding

ME is funded by a Senior Research Fellowship from the 10.13039/100004440Wellcome Trust (#207522).

ME & GW also receive salary support from the 10.13039/501100000272National Institute for Health Research (NIHR)(NIHR130812): Learning to Harness Innovation in Global Health for Quality Care (HIGH-Q) grant using UK aid from the UK government to support global health research.

## Declaration of Competing Interest

The authors declare the following financial interests/personal relationships which may be considered as potential competing interests: KK is a consultant for the World Health Organization on Networks of Care for maternal and newborn health. ME established the Clinical Information Network in Kenya and receives funding for this research platform.
