# Supplementary material for: Developing an understanding of networks with a focus on LMIC health systems: How and why clinical and programmatic networks form and function to be able to change practices: A realist review
Source: SSM Health Syst. 2023 Oct;1:100001. doi: 10.1016/j.ssmhs.2023.100001 (PMC10740353; doi:10.1016/j.ssmhs.2023.100001)
Supplement: Supplementary file 2 — Supplementary material [file mmc2.docx]

### Supplementary File 2. Update Database Search Strategies

Search Strategy for Medline (Ovid)

(health system network* OR clinical network OR networks of care OR inter-organization network* OR managed network* OR care network* OR hybrid network* OR integrated service delivery network* OR managed clinical network* OR program network* OR quality of care collaborative OR healthcare network* OR network based organization* OR network model OR network* relationship* OR networked governance OR integrated healthcare OR chain of care OR clinical community OR practitioner network* OR networks of health services OR delivery of healthcare OR health system planning OR organization of healthcare OR networks of clinical experts OR referral network* OR network initiative OR local network* OR district governance OR healthcare delivery OR (healthcare organization and administration) OR integrated delivery of healthcare OR network of safety OR provincial network of health services OR health network structures OR quality of care network OR regionalized healthcare OR network of healthcare interventions OR collaborative network OR network for healthcare practice improvement OR integrated system of care OR strategic clinical network* OR regional health network* OR integrated care network* OR network for quality improvement).mp.

AND

(health system* OR LMIC* OR developing countr* OR developing nation* OR developing population* OR developing world OR less developed countr* OR less developed nation* OR less developed population* OR less developed world OR lesser developed countr* OR lesser developed nation* OR lesser developed population* OR lesser developed world OR under developed countr* OR under developed nation* OR under developed population* OR under developed world OR underdeveloped countr* OR underdeveloped nation* OR underdeveloped population* OR underdeveloped world OR middle income countr* OR middle income nation* OR middle income population* OR low income countr* OR low income nation* OR low income population* OR lower income countr* OR lower income nation* OR lower income population* OR underserved countr* OR underserved nation* OR underserved population* OR underserved world OR under served countr* OR under served nation* OR under served population* OR under served world OR deprived countr* OR deprived nation* OR deprived population* OR deprived world OR poor countr* OR poor nation* OR poor population* OR poor world OR poorer countr* OR poorer nation* OR poorer population* OR poorer world OR developing econom* OR less developed econom* OR lesser developed econom* OR under developed econom* OR underdeveloped econom* OR middle income econom* OR low income econom* OR lower income econom* OR low gdp OR low gnp OR low gross domestic OR low gross national OR lower gdp OR lower gnp OR lower gross domestic OR lower gross national OR lmic OR lmics OR third world OR lami countr* OR transitional countr* OR Africa OR Asia OR Caribbean OR West Indies OR South America OR Latin America OR Central America OR Afghanistan OR Albania OR Algeria OR Angola OR Antigua OR Barbuda OR Argentina OR Armenia OR Armenian OR Aruba OR Azerbaijan OR Bahrain OR Bangladesh OR Barbados OR Benin OR Byelarus OR Byelorussian OR Belarus OR Belorussian OR Belorussia OR Belize OR Bhutan OR Bolivia OR Bosnia OR Herzegovina OR Hercegovina OR Botswana OR Brasil OR Brazil OR Bulgaria OR Burkina Faso OR Burkina Fasso OR Upper Volta OR Burundi OR Urundi OR Cambodia OR Khmer Republic OR Kampuchea OR Cameroon OR Cameroons OR Cameron OR Camerons OR Cape Verde OR Central African Republic OR Chad OR Chile OR China OR Colombia OR Comoros OR Comoro Islands OR Comores OR Mayotte OR Congo OR Zaire OR Costa Rica OR Cote d'Ivoire OR Ivory Coast OR Croatia OR Cuba OR Cyprus OR Czechoslovakia OR Czech Republic OR Slovakia OR Slovak Republic OR Djibouti OR French Somaliland OR Dominica OR Dominican Republic OR East Timor OR East Timur OR Timor Leste OR Ecuador OR Egypt OR United Arab Republic OR El Salvador OR Eritrea OR Estonia OR Ethiopia OR Fiji OR Gabon OR Gabonese Republic OR Gambia OR Gaza OR Georgia Republic OR Georgian Republic OR Ghana OR Gold Coast OR Greece OR Grenada OR Guatemala OR Guinea OR Guam OR Guiana OR Guyana OR Haiti OR Honduras OR Hungary OR India OR Maldives OR Indonesia OR Iran OR Iraq OR Isle of Man OR Jamaica OR Jordan OR Kazakhstan OR Kazakh OR Kenya OR Kiribati OR Korea OR Kosovo OR Kyrgyzstan OR Kirghizia OR Kyrgyz Republic OR Kirghiz OR Kirgizstan OR Lao PDR OR Laos OR Latvia OR Lebanon OR Lesotho OR Basutoland OR Liberia OR Libya OR Lithuania OR Macedonia OR Madagascar OR Malagasy Republic OR Malaysia OR Malaya OR Malay OR Sabah OR Sarawak OR Malawi OR Nyasaland OR Mali OR Malta OR Marshall Islands OR Mauritania OR Mauritius OR Agalega Islands OR Mexico OR Micronesia OR Middle East OR Moldova OR Moldovia OR Moldovian OR Mongolia OR Montenegro OR Morocco OR Ifni OR Mozambique OR Myanmar OR Myanma OR Burma OR Namibia OR Nepal OR Netherlands Antilles OR New Caledonia OR Nicaragua OR Niger OR Nigeria OR Northern Mariana Islands OR Oman OR Muscat OR Pakistan OR Palau OR Palestine OR Panama OR Paraguay OR Peru OR Philippines OR Philipines OR Phillipines OR Phillippines OR Poland OR Portugal OR Puerto Rico OR Romania OR Rumania OR Roumania OR Russia OR Russian OR Rwanda OR Ruanda OR Saint Kitts OR St Kitts OR Nevis OR Saint Lucia OR St Lucia OR Saint Vincent OR St Vincent OR Grenadines OR Samoa OR Samoan Islands OR Navigator Island OR Navigator Islands OR Sao Tome OR Saudi Arabia OR Senegal OR Serbia OR Montenegro OR Seychelles OR Sierra Leone OR Slovenia OR Sri Lanka OR Ceylon OR Solomon Islands OR Somalia OR Sudan OR Suriname OR Surinam OR Swaziland OR Syria OR Tajikistan OR Tadzhikistan OR Tadjikistan OR Tadzhik OR Tanzania OR Thailand OR Togo OR Togolese Republic OR Tonga OR Trinidad OR Tobago OR Tunisia OR Turkey OR Turkmenistan OR Turkmen OR Uganda OR Ukraine OR Uruguay OR USSR OR Soviet Union OR Union of Soviet Socialist Republics OR Uzbekistan OR Uzbek OR Vanuatu OR New Hebrides OR Venezuela OR Vietnam OR Viet Nam OR West Bank OR Yemen OR Yugoslavia OR Zambia OR Zimbabwe OR Rhodesia)

Limit to (english language and humans and yr=“2021 – current”)

Result: 556

Search Strategy for Embase (Ovid)

(health system network* OR clinical network OR networks of care OR inter-organization network* OR managed network* OR care network* OR hybrid network* OR integrated service delivery network* OR managed clinical network* OR program network* OR quality of care collaborative OR healthcare network* OR network based organization* OR network model OR network* relationship* OR networked governance OR integrated healthcare OR chain of care OR clinical community OR practitioner network* OR networks of health services OR delivery of healthcare OR health system planning OR organization of healthcare OR networks of clinical experts OR referral network* OR network initiative OR local network* OR district governance OR healthcare delivery OR (healthcare organization and administration) OR integrated delivery of healthcare OR network of safety OR provincial network of health services OR health network structures OR quality of care network OR regionalized healthcare OR network of healthcare interventions OR collaborative network OR network for healthcare practice improvement OR integrated system of care OR strategic clinical network* OR regional health network* OR integrated care network* OR network for quality improvement).mp.

AND

(health system* OR LMIC* OR developing countr* OR developing nation* OR developing population* OR developing world OR less developed countr* OR less developed nation* OR less developed population* OR less developed world OR lesser developed countr* OR lesser developed nation* OR lesser developed population* OR lesser developed world OR under developed countr* OR under developed nation* OR under developed population* OR under developed world OR underdeveloped countr* OR underdeveloped nation* OR underdeveloped population* OR underdeveloped world OR middle income countr* OR middle income nation* OR middle income population* OR low income countr* OR low income nation* OR low income population* OR lower income countr* OR lower income nation* OR lower income population* OR underserved countr* OR underserved nation* OR underserved population* OR underserved world OR under served countr* OR under served nation* OR under served population* OR under served world OR deprived countr* OR deprived nation* OR deprived population* OR deprived world OR poor countr* OR poor nation* OR poor population* OR poor world OR poorer countr* OR poorer nation* OR poorer population* OR poorer world OR developing econom* OR less developed econom* OR lesser developed econom* OR under developed econom* OR underdeveloped econom* OR middle income econom* OR low income econom* OR lower income econom* OR low gdp OR low gnp OR low gross domestic OR low gross national OR lower gdp OR lower gnp OR lower gross domestic OR lower gross national OR lmic OR lmics OR third world OR lami countr* OR transitional countr* OR Africa OR Asia OR Caribbean OR West Indies OR South America OR Latin America OR Central America OR Afghanistan OR Albania OR Algeria OR Angola OR Antigua OR Barbuda OR Argentina OR Armenia OR Armenian OR Aruba OR Azerbaijan OR Bahrain OR Bangladesh OR Barbados OR Benin OR Byelarus OR Byelorussian OR Belarus OR Belorussian OR Belorussia OR Belize OR Bhutan OR Bolivia OR Bosnia OR Herzegovina OR Hercegovina OR Botswana OR Brasil OR Brazil OR Bulgaria OR Burkina Faso OR Burkina Fasso OR Upper Volta OR Burundi OR Urundi OR Cambodia OR Khmer Republic OR Kampuchea OR Cameroon OR Cameroons OR Cameron OR Camerons OR Cape Verde OR Central African Republic OR Chad OR Chile OR China OR Colombia OR Comoros OR Comoro Islands OR Comores OR Mayotte OR Congo OR Zaire OR Costa Rica OR Cote d'Ivoire OR Ivory Coast OR Croatia OR Cuba OR Cyprus OR Czechoslovakia OR Czech Republic OR Slovakia OR Slovak Republic OR Djibouti OR French Somaliland OR Dominica OR Dominican Republic OR East Timor OR East Timur OR Timor Leste OR Ecuador OR Egypt OR United Arab Republic OR El Salvador OR Eritrea OR Estonia OR Ethiopia OR Fiji OR Gabon OR Gabonese Republic OR Gambia OR Gaza OR Georgia Republic OR Georgian Republic OR Ghana OR Gold Coast OR Greece OR Grenada OR Guatemala OR Guinea OR Guam OR Guiana OR Guyana OR Haiti OR Honduras OR Hungary OR India OR Maldives OR Indonesia OR Iran OR Iraq OR Isle of Man OR Jamaica OR Jordan OR Kazakhstan OR Kazakh OR Kenya OR Kiribati OR Korea OR Kosovo OR Kyrgyzstan OR Kirghizia OR Kyrgyz Republic OR Kirghiz OR Kirgizstan OR Lao PDR OR Laos OR Latvia OR Lebanon OR Lesotho OR Basutoland OR Liberia OR Libya OR Lithuania OR Macedonia OR Madagascar OR Malagasy Republic OR Malaysia OR Malaya OR Malay OR Sabah OR Sarawak OR Malawi OR Nyasaland OR Mali OR Malta OR Marshall Islands OR Mauritania OR Mauritius OR Agalega Islands OR Mexico OR Micronesia OR Middle East OR Moldova OR Moldovia OR Moldovian OR Mongolia OR Montenegro OR Morocco OR Ifni OR Mozambique OR Myanmar OR Myanma OR Burma OR Namibia OR Nepal OR Netherlands Antilles OR New Caledonia OR Nicaragua OR Niger OR Nigeria OR Northern Mariana Islands OR Oman OR Muscat OR Pakistan OR Palau OR Palestine OR Panama OR Paraguay OR Peru OR Philippines OR Philipines OR Phillipines OR Phillippines OR Poland OR Portugal OR Puerto Rico OR Romania OR Rumania OR Roumania OR Russia OR Russian OR Rwanda OR Ruanda OR Saint Kitts OR St Kitts OR Nevis OR Saint Lucia OR St Lucia OR Saint Vincent OR St Vincent OR Grenadines OR Samoa OR Samoan Islands OR Navigator Island OR Navigator Islands OR Sao Tome OR Saudi Arabia OR Senegal OR Serbia OR Montenegro OR Seychelles OR Sierra Leone OR Slovenia OR Sri Lanka OR Ceylon OR Solomon Islands OR Somalia OR Sudan OR Suriname OR Surinam OR Swaziland OR Syria OR Tajikistan OR Tadzhikistan OR Tadjikistan OR Tadzhik OR Tanzania OR Thailand OR Togo OR Togolese Republic OR Tonga OR Trinidad OR Tobago OR Tunisia OR Turkey OR Turkmenistan OR Turkmen OR Uganda OR Ukraine OR Uruguay OR USSR OR Soviet Union OR Union of Soviet Socialist Republics OR Uzbekistan OR Uzbek OR Vanuatu OR New Hebrides OR Venezuela OR Vietnam OR Viet Nam OR West Bank OR Yemen OR Yugoslavia OR Zambia OR Zimbabwe OR Rhodesia)

Limit to (english language and humans and yr=“2021 – current”)

Result: 872

Search Strategy for Global Health (Ovid)

(health system network* OR clinical network OR networks of care OR inter-organization network* OR managed network* OR care network* OR hybrid network* OR integrated service delivery network* OR managed clinical network* OR program network* OR quality of care collaborative OR healthcare network* OR network based organization* OR network model OR network* relationship* OR networked governance OR integrated healthcare OR chain of care OR clinical community OR practitioner network* OR networks of health services OR delivery of healthcare OR health system planning OR organization of healthcare OR networks of clinical experts OR referral network* OR network initiative OR local network* OR district governance OR healthcare delivery OR (healthcare organization and administration) OR integrated delivery of healthcare OR network of safety OR provincial network of health services OR health network structures OR quality of care network OR regionalized healthcare OR network of healthcare interventions OR collaborative network OR network for healthcare practice improvement OR integrated system of care OR strategic clinical network* OR regional health network* OR integrated care network* OR network for quality improvement).mp.

AND

(health system* OR LMIC* OR developing countr* OR developing nation* OR developing population* OR developing world OR less developed countr* OR less developed nation* OR less developed population* OR less developed world OR lesser developed countr* OR lesser developed nation* OR lesser developed population* OR lesser developed world OR under developed countr* OR under developed nation* OR under developed population* OR under developed world OR underdeveloped countr* OR underdeveloped nation* OR underdeveloped population* OR underdeveloped world OR middle income countr* OR middle income nation* OR middle income population* OR low income countr* OR low income nation* OR low income population* OR lower income countr* OR lower income nation* OR lower income population* OR underserved countr* OR underserved nation* OR underserved population* OR underserved world OR under served countr* OR under served nation* OR under served population* OR under served world OR deprived countr* OR deprived nation* OR deprived population* OR deprived world OR poor countr* OR poor nation* OR poor population* OR poor world OR poorer countr* OR poorer nation* OR poorer population* OR poorer world OR developing econom* OR less developed econom* OR lesser developed econom* OR under developed econom* OR underdeveloped econom* OR middle income econom* OR low income econom* OR lower income econom* OR low gdp OR low gnp OR low gross domestic OR low gross national OR lower gdp OR lower gnp OR lower gross domestic OR lower gross national OR lmic OR lmics OR third world OR lami countr* OR transitional countr* OR Africa OR Asia OR Caribbean OR West Indies OR South America OR Latin America OR Central America OR Afghanistan OR Albania OR Algeria OR Angola OR Antigua OR Barbuda OR Argentina OR Armenia OR Armenian OR Aruba OR Azerbaijan OR Bahrain OR Bangladesh OR Barbados OR Benin OR Byelarus OR Byelorussian OR Belarus OR Belorussian OR Belorussia OR Belize OR Bhutan OR Bolivia OR Bosnia OR Herzegovina OR Hercegovina OR Botswana OR Brasil OR Brazil OR Bulgaria OR Burkina Faso OR Burkina Fasso OR Upper Volta OR Burundi OR Urundi OR Cambodia OR Khmer Republic OR Kampuchea OR Cameroon OR Cameroons OR Cameron OR Camerons OR Cape Verde OR Central African Republic OR Chad OR Chile OR China OR Colombia OR Comoros OR Comoro Islands OR Comores OR Mayotte OR Congo OR Zaire OR Costa Rica OR Cote d'Ivoire OR Ivory Coast OR Croatia OR Cuba OR Cyprus OR Czechoslovakia OR Czech Republic OR Slovakia OR Slovak Republic OR Djibouti OR French Somaliland OR Dominica OR Dominican Republic OR East Timor OR East Timur OR Timor Leste OR Ecuador OR Egypt OR United Arab Republic OR El Salvador OR Eritrea OR Estonia OR Ethiopia OR Fiji OR Gabon OR Gabonese Republic OR Gambia OR Gaza OR Georgia Republic OR Georgian Republic OR Ghana OR Gold Coast OR Greece OR Grenada OR Guatemala OR Guinea OR Guam OR Guiana OR Guyana OR Haiti OR Honduras OR Hungary OR India OR Maldives OR Indonesia OR Iran OR Iraq OR Isle of Man OR Jamaica OR Jordan OR Kazakhstan OR Kazakh OR Kenya OR Kiribati OR Korea OR Kosovo OR Kyrgyzstan OR Kirghizia OR Kyrgyz Republic OR Kirghiz OR Kirgizstan OR Lao PDR OR Laos OR Latvia OR Lebanon OR Lesotho OR Basutoland OR Liberia OR Libya OR Lithuania OR Macedonia OR Madagascar OR Malagasy Republic OR Malaysia OR Malaya OR Malay OR Sabah OR Sarawak OR Malawi OR Nyasaland OR Mali OR Malta OR Marshall Islands OR Mauritania OR Mauritius OR Agalega Islands OR Mexico OR Micronesia OR Middle East OR Moldova OR Moldovia OR Moldovian OR Mongolia OR Montenegro OR Morocco OR Ifni OR Mozambique OR Myanmar OR Myanma OR Burma OR Namibia OR Nepal OR Netherlands Antilles OR New Caledonia OR Nicaragua OR Niger OR Nigeria OR Northern Mariana Islands OR Oman OR Muscat OR Pakistan OR Palau OR Palestine OR Panama OR Paraguay OR Peru OR Philippines OR Philipines OR Phillipines OR Phillippines OR Poland OR Portugal OR Puerto Rico OR Romania OR Rumania OR Roumania OR Russia OR Russian OR Rwanda OR Ruanda OR Saint Kitts OR St Kitts OR Nevis OR Saint Lucia OR St Lucia OR Saint Vincent OR St Vincent OR Grenadines OR Samoa OR Samoan Islands OR Navigator Island OR Navigator Islands OR Sao Tome OR Saudi Arabia OR Senegal OR Serbia OR Montenegro OR Seychelles OR Sierra Leone OR Slovenia OR Sri Lanka OR Ceylon OR Solomon Islands OR Somalia OR Sudan OR Suriname OR Surinam OR Swaziland OR Syria OR Tajikistan OR Tadzhikistan OR Tadjikistan OR Tadzhik OR Tanzania OR Thailand OR Togo OR Togolese Republic OR Tonga OR Trinidad OR Tobago OR Tunisia OR Turkey OR Turkmenistan OR Turkmen OR Uganda OR Ukraine OR Uruguay OR USSR OR Soviet Union OR Union of Soviet Socialist Republics OR Uzbekistan OR Uzbek OR Vanuatu OR New Hebrides OR Venezuela OR Vietnam OR Viet Nam OR West Bank OR Yemen OR Yugoslavia OR Zambia OR Zimbabwe OR Rhodesia)

Limit 1 to (english language and yr=“2021 – current”

Result: 275

Search for Web of Science

TS=(“health system network”* OR “clinical network” OR “networks of care” OR “inter-organization network”* OR “managed network”* OR “care network”* OR “hybrid network”* OR “integrated service delivery network”* OR “managed clinical network”* OR “program network”* OR “quality of care collaborative” OR “healthcare network”* OR “network based organization”* OR “network model” OR “network* relationship*” OR “networked governance” OR “integrated healthcare” OR “chain of care” OR “clinical community” OR “practitioner network*” OR “networks of health services” OR “delivery of healthcare” OR “health system planning” OR “organization of healthcare” OR “networks of clinical experts” OR “referral network*” OR “network initiative” OR “local network*” OR “district governance” OR “healthcare delivery” OR “healthcare organization and administration” OR “integrated delivery of healthcare” OR “network of safety” OR “provincial network of health services” OR “health network structures” OR “quality of care network” OR “regionalized healthcare” OR “network of healthcare interventions” OR “collaborative network” OR “network for healthcare practice improvement” OR “integrated system of care” OR “strategic clinical network*” OR “regional health network*” OR “integrated care network*” OR “network for quality improvement”)

AND

TS=(LMIC OR "developing countr*" OR "health system*" “developing nation*” OR “developing population*” OR “developing world” OR “less developed countr*” OR “less developed nation*” OR “less developed population*” OR “less developed world” OR “lesser developed countr*” OR “lesser developed nation*” OR “lesser developed population*” OR “lesser developed world” OR “under developed countr*” OR “under developed nation*” OR “under developed population*” OR “under developed world” OR “underdeveloped countr*” OR “underdeveloped nation*” OR “underdeveloped population*” OR “underdeveloped world” OR “middle income countr*” OR “middle income nation*” OR “middle income population*” OR “low income countr*” OR “low income nation*” OR “low income population*” OR “lower income countr*” OR “lower income nation*” OR “lower income population*” OR “underserved countr*” OR “underserved nation*” OR “underserved population*” OR “underserved world” OR “under served countr*” OR “under served nation*” OR “under served population*” OR “under served world” OR “deprived countr*” OR “deprived nation*” OR “deprived population*” OR “deprived world” OR “poor countr*” OR “poor nation*” OR “poor population*” OR “poor world” OR “poorer countr*” OR “poorer nation*” OR “poorer population*” OR “poorer world” OR “developing econom*” OR “less developed econom*” OR “lesser developed econom*” OR “under developed econom*” OR “underdeveloped econom*” OR “middle income econom*” OR “low income econom*” OR “lower income econom*” OR “low gdp” OR “low gnp” OR “low gross domestic” OR “low gross national” OR “lower gdp” OR “lower gnp” OR “lower gross domestic” OR “lower gross national” OR lmic OR lmics OR “third world” OR “lami countr*” OR “transitional countr*” OR Africa OR Asia OR Caribbean OR “West Indies” OR “South America” OR “Latin America” OR “Central America” OR Afghanistan OR Albania OR Algeria OR Angola OR Antigua OR Barbuda OR Argentina OR Armenia OR Armenian OR Aruba OR Azerbaijan OR Bahrain OR Bangladesh OR Barbados OR Benin OR Byelarus OR Byelorussian OR Belarus OR Belorussian OR Belorussia OR Belize OR Bhutan OR Bolivia OR Bosnia OR Herzegovina OR Hercegovina OR Botswana OR Brasil OR Brazil OR Bulgaria OR “Burkina Faso” OR “Burkina Fasso” OR “Upper Volta” OR Burundi OR Urundi OR Cambodia OR “Khmer Republic” OR Kampuchea OR Cameroon OR Cameroons OR Cameron OR Camerons OR “Cape Verde” OR “Central African Republic” OR Chad OR Chile OR China OR Colombia OR Comoros OR “Comoro Islands” OR Comores OR Mayotte OR Congo OR Zaire OR “Costa Rica” OR “Cote d'Ivoire” OR “Ivory Coast” OR Croatia OR Cuba OR Cyprus OR Czechoslovakia OR “Czech Republic” OR Slovakia OR “Slovak Republic” OR Djibouti OR “French Somaliland” OR Dominica OR “Dominican Republic” OR “East Timor” OR “East Timur” OR “Timor Leste” OR Ecuador OR Egypt OR “United Arab Republic” OR “El Salvador” OR Eritrea OR Estonia OR Ethiopia OR Fiji OR Gabon OR “Gabonese Republic” OR Gambia OR Gaza OR “Georgia Republic” OR “Georgian Republic” OR Ghana OR “Gold Coast” OR Greece OR Grenada OR Guatemala OR Guinea OR Guam OR Guiana OR Guyana OR Haiti OR Honduras OR Hungary OR India OR Maldives OR Indonesia OR Iran OR Iraq OR “Isle of Man” OR Jamaica OR Jordan OR Kazakhstan OR Kazakh OR Kenya OR Kiribati OR Korea OR Kosovo OR Kyrgyzstan OR Kirghizia OR “Kyrgyz Republic” OR Kirghiz OR Kirgizstan OR “Lao PDR” OR Laos OR Latvia OR Lebanon OR Lesotho OR Basutoland OR Liberia OR Libya OR Lithuania OR Macedonia OR Madagascar OR “Malagasy Republic” OR Malaysia OR Malaya OR Malay OR Sabah OR Sarawak OR Malawi OR Nyasaland OR Mali OR Malta OR “Marshall Islands” OR Mauritania OR Mauritius OR “Agalega Islands” OR Mexico OR Micronesia OR “Middle East” OR Moldova OR Moldovia OR Moldovian OR Mongolia OR Montenegro OR Morocco OR Ifni OR Mozambique OR Myanmar OR Myanma OR Burma OR Namibia OR Nepal OR “Netherlands Antilles” OR “New Caledonia” OR Nicaragua OR Niger OR Nigeria OR “Northern Mariana Islands” OR Oman OR Muscat OR Pakistan OR Palau OR Palestine OR Panama OR Paraguay OR Peru OR Philippines OR Philipines OR Phillipines OR Phillippines OR Poland OR Portugal OR “Puerto Rico” OR Romania OR Rumania OR Roumania OR Russia OR Russian OR Rwanda OR Ruanda OR “Saint Kitts” OR “St Kitts” OR Nevis OR “Saint Lucia” OR “St Lucia” OR “Saint Vincent” OR “St Vincent” OR Grenadines OR Samoa OR “Samoan Islands” OR “Navigator Island” OR “Navigator Islands” OR “Sao Tome” OR “Saudi Arabia” OR Senegal OR Serbia OR Montenegro OR Seychelles OR “Sierra Leone” OR Slovenia OR “Sri Lanka” OR Ceylon OR Solomon Islands OR Somalia OR Sudan OR Suriname OR Surinam OR Swaziland OR Syria OR Tajikistan OR Tadzhikistan OR Tadjikistan OR Tadzhik OR Tanzania OR Thailand OR Togo OR “Togolese Republic” OR Tonga OR Trinidad OR Tobago OR Tunisia OR Turkey OR Turkmenistan OR Turkmen OR Uganda OR Ukraine OR Uruguay OR USSR OR “Soviet Union” OR “Union of Soviet Socialist Republics” OR Uzbekistan OR Uzbek OR Vanuatu OR “New Hebrides” OR Venezuela OR Vietnam OR “Viet Nam” OR “West Bank” OR Yemen OR Yugoslavia OR Zambia OR Zimbabwe OR Rhodesia)

IC Timespan=2021-2022

Refined by: WEB OF SCIENCE CATEGORIES: (PUBLIC ENVIRONMENTAL OCCUPATIONAL HEALTH OR SOCIAL SCIENCES INTERDISCIPLINARY OR HEALTH CARE SCIENCES SERVICES OR MEDICINE GENERAL INTERNAL OR HEALTH POLICY SERVICES OR TROPICAL MEDICINE OR DEVELOPMENT STUDIES OR PEDIATRICS OR INTERNATIONAL RELATIONS OR SOCIAL SCIENCES BIOMEDICAL OR OPERATIONS RESEARCH MANAGEMENT SCIENCE OR NURSING OR PRIMARY HEALTH CARE OR OBSTETRICS GYNECOLOGY)

Not Document types: meeting abstracts or letters or proceedings papers

Results: 377
