# Supplementary material for: Developing an understanding of networks with a focus on LMIC health systems: How and why clinical and programmatic networks form and function to be able to change practices: A realist review
Source: SSM Health Syst. 2023 Oct;1:100001. doi: 10.1016/j.ssmhs.2023.100001 (PMC10740353; doi:10.1016/j.ssmhs.2023.100001)
Supplement: Supplementary file 4 — Supplementary material [file mmc4.docx]

| **#** | **Citation** | **Country** | **Study Methods** | **Type of Network** | **Contributes to CMOCs** |
| --- | --- | --- | --- | --- | --- |
| **Peer-reviewed literature** | | | | | |
| 1 | Adams V, Craig S, Samen A, Bhatta S. It Takes More than a Village: Building a Network of Safety in Nepal's Mountain Communities. Maternal and Child Health Journal. 2016; 20:19, 2424-2430. | Nepal | Report | Network of safety | 3C |
| 2 | Bhatta S, Rajbhandari S, Kalaris K, Carmone AE. The Logarithmic Spiral of Networks of Care for Expectant Families in Rural Nepal: A Descriptive Case Study. Health Systems and Reform. 2020; 6:2. doi:10.1080/23288604.2020.1824520. | Nepal | Descriptive case study | Network of Care/network of safety | 1D, 7D, 7E |
| 3 | Broughton E, Hermida J, Hill K, Sloan N, Chavez M, Gonzalez D, Freire JM, Ximena Gudino. Evaluation of an intervention to improve essential obstetric and newborn care access and quality in Cotopaxi, Ecuador. Frontiers in Public Health. 2016; 4:247. doi: 10.3389/fpubh.2016.00247. | Ecuador | Programme overview and evaluation | Provincial to community level MNH QI network | 4G, 7D |
| 4 | Burns E, Collington M, Eden T, Freccero P, Renner L; Paintsil V, Dolendo M, Islam A, Khaing AA, Rosser J. Development of paediatric oncology shared-care networks in low-middle income countries. Journal of Cancer Policy. 2018; 16:26-32. doi: 10.1016/j.jcpo.2018.03.003. | Ghana, Bangladesh | Report | Shared-care network | 2F, 4D |
| 5 | Chan BTB, Rauscher C, Issina AM, Kozhageldiyeva LH, Kuzembaeva DD, Davis CL, Kravchenko H, Hindmarsh M, McGowan J, Kulkaeva G. A programme to improve quality of care for patients with chronic diseases, Kazakhstan. Bulletin of the World Health Organization. 2020; 98:3, 161-169. doi:10.2471/BLT.18.227447. | Kazakhstan | Quasi-experimental evaluation | Disease management network | 1D |
| 6 | Cordier LF, Kalaris K, Rakotonanahary RJL, Rakotonirina L, Haruna J, Mayfield A, Marovavy L, McCarty MG, Aina AT, Ratsimbazafy B, Razafinjato B, Loyd T, Ihantamalala F, Garchitorena A, Bonds MH, Finnegan KE. Networks of Care in Rural Madagascar for Achieving Universal Health Coverage in Ifanadiana District. Health Systems & Reform. 2020; 6:2, e1841437. doi: 10.1080/23288604.2020.1841437. | Madagascar | Descriptive case study | Network of Care | 2F, 4D |
| 7 | D'Mello BS, Bwile P, Carmone AE, Kalaris K, Magembe G, Masweko M, Mtumbuka E, Mushi T, Sellah Z, Gichanga B. Averting Maternal Death and Disability in an Urban Network of Care in Dar es Salaam, Tanzania: A Descriptive Case Study. Health Systems & Reform. 2020; 6:2. e1834303. doi: 10.1080/23288604.2020.1834303. | Tanzania | Descriptive case study | Network of Care | 4G, 5J, 7C, 8A |
| 8 | English M, Ayieko P, Nyamai R, Were F, Githanga D, Irimu G. What do we think we are doing? How might a clinical information network be promoting implementation of recommended paediatric care practices in Kenyan hospitals? Health Research Policy and Systems. 2017; 15:4. doi: 10.1186/s12961-017-0172-1. | Kenya | Realist informed programme overview | Clinical Information Network | 2E, 5B, 5C, 5D, 6E, 8D |
| 9 | Fasawe O, Adekeye O, Carmone AE, Dahunsi O, Kalaris K, Storey A, Osy U, Wiwa O. Applying a Clientcentered Approach to Maternal and Neonatal Networks of Care: Case Studies from Urban and Rural Nigeria. Health Systems & Reform. 2020; 6:2, e1841450. doi: 10.1080/23288604.2020.1841450. | Nigeria | Descriptive case study | Network of Care | 7E |
| 10 | Gachau S, Ayieko P, Gathara D, Mwaniki P, Ogero M, Akech S, Maina M, Agweyu A, Oliwa J, Julius T, Malla L, Wafula J, Mbevi G, Irimu G, English M. Does audit and feedback improve the adoption of recommended practices? Evidence from a longitudinal observational study of an emerging clinical network in Kenya. BMJ Global Health. 2017; 2:e000468. doi:10.1136/bmjgh-2017-000468. | Kenya | Longitudinal observational study | Clinical Information Network | 5H, 5J |
| 11 | Hyre A, Caiola N, Amelia D, Gandawidjaja T, Markus S, Baharuddin M. Expanding Maternal and Neonatal Survival in Indonesia: A program overview. International Journal of Gynecology & Obstetrics. 2019; 144. doi: 10.1002/ijgo.12730. | Indonesia | Programme overview | Referral networks | 2F, 7D, 8A |
| 12 | Iedema R, Verma R, Wutzke S, Lyons N, McCaughan B. A network of networks. Journal of Health Organization & Management. 2017; 31:2. doi:10.1108/JHOM-07-2016-0146. | Australia | Ethnographic descriptive case study | Network of networks made up of taskforces, institutes, and clinical networks | 2B, 3B, 5C |
| 13 | Irimu G, Ogero M, Mbevi G, Agweyu A, Akech S, Julius T, Nyamai R, Githang’a D, Ayieko P, English M. Approaching quality improvement at scale: a learning health system approach in Kenya. Arch Dis Child. 2018. doi: 10.1136/archdischild-2017-314348. | Kenya | Programme overview | Clinical Information Network | 5K, 6B, 6C, 6D, 6E, 7D, 8A, 8D, 8F |
| 14 | Leslie M, Khayatzadeh-Mahani A, Birdsell J, Forest P, G, Henderson R, Gray RP, Schraeder K, Seidel J, Zwicker J, Green LA. An implementation history of primary health care transformation: Alberta's primary care networks and the people, time and culture of change. BMC Family Practice. 2020; 21:258. doi: 10.1186/s12875-020-01330-7. | Canada | Interpretative qualitative study | Primary Care Networks | 1C, 2B, 2C, 6B, 6E |
| 15 | Lopez-Vazquez J, Perez-Martinez DE, Vargas I, Vazquez ML. Interventions to Improve Clinical Coordination between Levels: Participatory Experience in a Public Healthcare Network in Xalapa, Mexico. International Journal of Integrated Care. 2021; 21(4):12, 1-17. doi:  10.5334/ijic.5892. | Mexico | Qualitative, descriptive-interpretative study | Public Healthcare Network | 4E, 7B, 7D, 9B |
| 16 | McGivern G, Nzinga J, English M. 'Pastoral practices' for quality improvement in a Kenyan clinical network. Social Science & Medicine. 2017; 195:115-122. doi:/10.1016/j.socscimed.2017.11.031. | Kenya | Qualitative case study | Clinical Information Network | 1A, 2H, 3D, 5D, 5G, 5H, 5I, 5J, 6A, 6D, 7C, 8D, 9B, 9D |
| 17 | McInnes E, Middleton S, Gardner G, Haines M, Maertsch M, Paul CL, Castaldi P. A qualitative study of stakeholder views of the conditions for and outcomes of successful clinical networks. BMC Health Services Research. 2012; 12:49. doi:10.1186/1472-6963-12-49. | Australia | Qualitative study | Clinical networks | 2H, 4D, 4G, 5C, 5E, 5G, 5I, 5K, 7B, 8A, 8D, 9B |
| 18 | McInnes E, Haines M, Dominello A, Kalucy D, Jammali-Blasi A, Middleton S, Klienberg E. What are the reasons for clinical network success? A qualitative study. BMC Health Services Research. 2015; 15: 479. doi: 10.1186/s12913-015-1096-5. | Australia | Qualitative study | Clinical networks | 2C, 2G, 4G, 5A, 5B, 5E, 5G, 5L, 5M, 8D, 8F |
| 19 | Mullany LC, Lee CI, Paw P, Od EKS, Maung C, Kuiper H, Mansenior N, Beyrer C, Lee TJ. The MOM Project: Delivering maternal health services among internally displaced populations in eastern Burma. Reproductive Health Matters. 2008;16:31, 44-56. doi: 10.1016/S0968-8080(08)31341-X. | Burma | Programme overview | Three-tiered collaborative network of community-based maternal health workers | 7D |
| 20 | Nahimana E, McBain R, Manzi A, Iyer H, Uwingabiye A, Gupta N, Muzungu G, Drobac P, Hirschhorn LR. Race to the Top: evaluation of a novel performance-based financing initiative to promote healthcare delivery in rural Rwanda. Global Health Action. 2016; 9:1, 32943, doi: 10.3402/gha.v9.32943. | Rwanda | Programme overview and evaluation | PBF linked quality improvement collaborative program | 7D |
| 21 | Pittalis C, Brugha R, Bijlmakers L, Cunningham F, Mwapasa G, Clarke M, Broekhuizen H, Ifeanyichi M, Borgstein E, Gajewski J. Using Network and Complexity Theories to Understand the Functionality of Referral Systems for Surgical Patients in Resource-Limited Settings, the Case of Malawi. International Journal of Health Policy and Management. 2021; 1-12. doi:  10.34172/ijhpm.2021.175. | Malawi | Mixed methods study | Referral network | 2B, 2G, 2H, 3C, 4B, 8C, 9D, 9F |
| 22 | Rycroft-Malone J, Burton CR, Wilkinson J, Harvey G, McCormack B, Baker R, Dopson S, Graham ID, Staniszewska S, Thompson C. Collective action for implementation: a realist evaluation of organisational collaboration in healthcare. Implement Sci. 2016;11:17. | UK | Longitudinal realist evaluation | Collaborations for Leadership in Applied Health Research in Care | 2B, 4C, 8A |
| 23 | Schneider H, George A, Mukinda F, Tabana H. District Governance and Improved Maternal, Neonatal and Child Health in South Africa: Pathways of Change. 2020; 6:1, e1669943, doi: 10.1080/23288604.2019.1669943. | South Africa | Qualitative case study | Monitoring and Response Unit | 2A, 2G, 3B, 4A, 4F, 5A, 5K, 6E, 8D, 9D |
| 24 | Sibbald S, Schouten K, Sedig K, Maskell R, Licskai C. Key characteristics and critical junctures for successful Interprofessional networks in healthcare - a case study. BMC Health Services Research. 2020; 20:700. doi: 10.1186/s12913-020-05565-z. | Canada | Exploratory case study | Primary Care Innovation Collective | 2D, 2B, 2C, 2E, 3A, 5B, 7A, 9A, 9B, 9C, 9E |
| 25 | Srivastava S, Datta V, Garde R, Singh M, Sooden A, Pemde H, Jain M, Shivkumar P, Bang A, Kumari P, Makhija S, Ravi T, Mehta S, Garg BS, Mehta R. Development of a hub and spoke model for quality improvement in rural and urban healthcare settings in India: a pilot study. BMJ Open Quality. 2020; 9:e000908. doi: 10.1136/bmjoq-2019-000908. | India | Programme overview and evaluation | Quality improvement network | 6D, 7D, 8A, 9A |
| 26 | Tuti T, Bitok M, Malla L, Paton C, Muinga N, Gathara D, Gachau S, Mbevi G, Nyachiro W, Ogero M, et al. Improving documentation of clinical care within a clinical information network: an essential initial step in efforts to understand and improve care in Kenyan hospitals. BMJ Global Health. 2016; 1(1):e000028. doi:10.1136/bmjgh-2016-000028. | Kenya | Programme overview and assessment | Clinical Information Network | 5H, 6D |
| 27 | Vargas I, Eguiguren P, Mogollón-Pérez AS, Samico I, Bertolotto F, López-Vázquez J, Vázquez ML. Can care coordination across levels be improved through the implementation of participatory action research interventions? Outcomes and conditions for sustaining changes in five Latin American countries. BMC Health Services Research. 2020; 20:941. doi: 10.1186/s12913-020-05781-7. | Brazil  Chile  Colombia  Mexico  Uruguay | Qualitative descriptive-interpretative study | Public Healthcare Networks | 1D, 2B, 4D, 4F, 6A, 6D |
| 28 | Vargas I, Eguiguren P, Mogollon-Pérez AS, Bertolotto F, Samico I, Lopez J, De Paepe P, Vazquez ML. Understanding the factors influencing the implementation of participatory interventions to improve care coordination. An analytical framework based on an evaluation in Latin America. Health Policy and Planning. 2020;35: 962-972. doi: doi: 10.1093/heapol/czaa066. | Brazil  Chile  Colombia  Mexico  Uruguay | Qualitative descriptive-interpretative study | Public Healthcare Networks | 1B, 4C, 5F, 7A, 7B, 8A, 8B, |
| 29 | Vergara MTM, de Vera EA, Carmone AE. Building Trust to Save Lives in a Metro Manila Public-Private Network of Care: A Descriptive Case Study of Quirino Recognized Partners in Quezon City, Philippines. Health Systems & Reform. 2020; 6:2, e1815473, doi: 10.1080/23288604.2020.1815473. | Philippines | Descriptive case study | Network of Care | 2E, 3C, 5L, 6E, 7C, 7D, 8E, 9A |
|  | **Grey literature** |  |  |  |  |
| 30 | Kirunda R, Mubiru F, Akumu E, Wynne L, Rakhmanova N, Kim C. Applying a Quality Improvement Model to Strengthen Community-based Family Planning Services in Busia District, Uganda. Issue 1. 2016. | Uganda | Brief | Service delivery network | 1D |
| 31 | USAID/Engender Health. A collaborative network to improve access to fistula treatment in Nigeria. (Fistula Care). 2010. | Nigeria | Brief | Clinical peer-support network | 4D, 8A |
| 32 | USAID. Establishing Model Referral Networks in Haiti: MCSP Case Study. 2018. | Haiti | Brief | Referral network | 4G |
