# Supplementary material for: Developing an understanding of networks with a focus on LMIC health systems: How and why clinical and programmatic networks form and function to be able to change practices: A realist review
Source: SSM Health Syst. 2023 Oct;1:100001. doi: 10.1016/j.ssmhs.2023.100001 (PMC10740353; doi:10.1016/j.ssmhs.2023.100001)
Supplement: Supplementary file 5 — Supplementary material [file mmc5.docx]

## Supplementary File 5. Context-Mechanism-Outcome Configurations and supporting data

### Identify a problem

| 1A. When potential network members feel a sense of dissatisfaction (misaligned with expectations or values) with an issue (e.g. in clinical care, service delivery organisation, or health system management) (context), they will have the energy, excitement, and motivation to do something about it (outcome) because of their frustration (mechanism) | *“Yet good intentions were frustrated by the Kenyan health system. Paediatrician 3 noted: “Frustration is actually top on the list… I wish I had this [equipment/drug], I would be able to save this baby … your potential is being utilized 20–30%, you get bored … your hands are tied.” This undermined motivation to improve care. Paediatrician 21 noted: “Motivation … is gone. You go to work at eight and you leave at four. It’s just work now, it’s not how can I make [health care] better.”” (McGivern et al 2017)* |
| --- | --- |
| 1B. When a network is in the initial stages and potential network members reflect together on problems (context) they will recruit others to the cause (outcome) because there is a realisation that they can be part of the solution to some degree (mechanism) | *“So when it’s an idea, a strategy that comes from the group, it’s already born with everybody willing and able, committed to the implementation process, which we knew was going to involve a set of challenges (LSC, Chile)” (Vargas et al 2 2020)* |
| 1C. When potential network members share and discuss their collective experiences, emotions, understanding, or perspectives for a commonly felt problem (context) they are better able to understand what needs to be done and find solutions (outcome) because they have more knowledge to drawn on (mechanism) | *“At a time of significant upheaval for the RHAs – they were in the throes of amalgamating into AHS – a small group of independent, lone, long-term generalists working in a field technically and culturally peripheral to what was seen as core healthcare operations, began talking to one another and directly to politicians. Out of these LPCI conversations came a vision, and the technical details of the PCNs. As one policy-maker noted, the vision coalesced around local solutions for local problems.” (Leslie et al 2020)* |
| 1D. When members in an established network feel it is safe to critically examine and reflect on existing practices (context), they are able to identify new problems and potential solutions (outcome) because they feel enabled to challenge the status quo / feel in a psychological safe space to be able to challenge the status quo (mechanism) | *“During learning sessions and coaching visits, implementation problems were observed, such as the incomplete use of flowsheets, data entry errors, incorrect techniques in patient self-management discussions and confusion about interpreting guidelines, algorithms or indicators. The traditional learning model of attending a single lecture would probably not have resulted in similar improvements” (Chan et al 2020)*  *“In February 2015 APC, implemented by FHI 360 in Uganda, conducted a collaborative site assessment for CBFP programs with the MOH in the three pilot sites, Bulumbi, Buhehe and Buteba. The assessment identified areas for service delivery improvement, which were quantified by the QI monitoring that started in June 2015: • Inadequate counseling of clients by VHTs, especially on the possible side effects of long acting and reversible contraceptives (LARCs). On average, only 30% of clients were receiving counseling that included all the steps listed in the MOH FP counseling flipchart. • Low FP continuation: Only about 25% of clients on average return to the VHTs on time for resupply or reinjection of a method. Through a root cause analysis, stakeholders identified common reasons why women are not using family planning methods that are available and accessible through the VHTs. The reasons cited include opposition from male partners, side effects experienced by clients, and myths and misconceptions about family planning among men and women; these reasons are also supported by the literature.6,7 VHTs identified addressing side effects and misconceptions, as well as male involvement, as key to uptake and continued use of FP methods.” (Kirunda et al 2016)*  *"OHW conducted a landscape analysis that resulted in a long list of improvements required and then carried out across 35 facilities in order to facilitate treatment. Subsequent monitoring, however, found no impact; although equipment to treat jaundice was now available, it was not being used. Realizing that there was a piece missing, and because the Network of Safety model is committed to the process of expanding access to good quality care, additional attention was dedicated to evaluating why the expected outcomes were not achieved. Upon reexamination of the cascade of care, consequential details became clear. Most babies were being discharged home with their mothers within 24 hours after birth, which is too early to see signs of jaundice on physical examination. Providers had no way to identify babies with hyperbilirubinemia at the earlier stage, before days 3–5 when jaundice typically becomes physically apparent. Because the neonates with jaundice were not being identified, treatment was not being given. Initial indications suggest that the addition of one simple diagnostic tool, a transcutaneous bilirubin meter, may make a difference in identifying neonates with jaundice early so that treatment can be initiated through the referral hospitals newly capacitated with Special Newborn Care Units (SNCU) equipped with phototherapy devices." (Bhatta et al 2020)*  *“Helped to get to know professionals of the other level and their work context and fostered personal connections “I’ve got the impression, as I also participate in the consultations and videoconferences, that ties have been made between primary care and the specialists (…). We often have that frank conversation of: look, this is what’s going on, or this is the real case scenario, as seen from primary care and also as seen from the hospital perspective (…) so that leads to this kind of ‘Ohhh, right! Well let’s see how we can put our heads together and solve this then’” (Healthcare Manager, Chile). (Vargas et al 2020)*  *“According to the majority of informants in Chile, the cross-level bidirectional visits between PC and SC improved administrative coordination between levels because they permited direct communication between professionals to facilitate access to specialist care and monitor the patient’s transition between levels, joint problem solving in administrative matters, and a better knowledge of the administrative pathways and access requirements of the other care level (Table 2.4): “(…) we started to get to know the secondary level problems over there and they got to know the problems we face here. We started to realise that we had nearly the same problems and that there were lots of things we could solve. That also facilitated almost direct communication, one on one, the person in charge of one thing here liaising with the person in charge of the same thing there (...) so it flowed better, shall we say, the care of the patient” (Healthcare Manager, Chile). Informants reported that the improved administrative coordination helped to reduce waiting times and administratively deficient referrals (incomplete information, etc.) and to provide the patient with more adequate information on the procedure.” (Vargas et al 2020)* |

### Developing a collective vision

| 2A. When potential network members engage in an open process of collective sense-making around a problem (context), they can identify what they share in common (outcome) because they learn about each other’s experiences, emotions, understandings, or perspectives (mechanism) | *“An initial formative event was a consensus meeting to establish the CIN’s vision and mission. It was attended by partner representatives and a focal team from each hospital, comprising a paediatrician, a nurse heading the paediatric ward and the officer in charge of health records and information. This meeting helped align participants’ goals drawing on shared values and resources as part of collective meaning-making and resulted in vision and mission statements.” (Irimu et al 2018)*  *“During the course of the visits to the districts, sub- districts and facilities, the facilitator was also actively engaged in a process of communication and collective sense-making, building consensus on firstly, the problem to be prioritized (high MNC mortality) and secondly, a set of appropriate responses to the identified problem. The latter entailed presenting the evidence base on interventions for MNCH, drawing attention to existing guidelines, and packaging formal information in ways that promoted system thinking and enabled individual players to locate their place in the whole.” (Schneider et al 2020)* |
| --- | --- |
| 2B. If potential network members identify and articulate commonalities among each other (context) then this enables the development of a collective network vision (outcome) because they understand each other’s perspectives (mechanism) | *“The PAR process improved interactional factors between managers of the network “(…) Hospital: “There’s nothing more I can do because primary care is doing such a terrible job”. Primary care centre: “There’s nothing more I can do because the hospital is doing such a bad job” (…) And with that we don’t get anywhere constructive (…) I would say that this in some way....means we basically see ourselves as one and the same. So we’ve really managed to lower our defensive barriers (…) and start to understand that what we really need to do is help each other out” (LSC, Chile). “it’s like a sign that a shared vision of the network is appearing, which was the problem we had in the first place (…) Of course, and people perceive it and everyone can feel it now. If I’ve got a problem I know who to speak to and I know they’re going to help, you know? And the certainty that they’ll help me, that they’ll collaborate with me for the sake of the patient”” (LSC, Chile) (Vargas et al 2020)*  *“Our findings demonstrate that when beliefs, values and priorities among members of the referral network converge, this fosters cooperative behaviours which are beneficial for individuals and the network as a whole. When values, beliefs and priorities diverge, self-interest prevails to the benefit of individual clinicians but impacting negatively on other clinicians and potentially on patients.” (Pittalis et al 2021)*  *“The paper concluded by labelling ACI as a “network of networks”. This phrase served to describe ACI as leveraging not merely the collaborative enthusiasm of stakeholders with specific clinical or scientific agendas, but also the energy that emerges from the co-location of and dynamic interaction among healthcare networks, enabling them to explore and capitalise on commonalities and shared concerns. Here, the uniqueness of ACI’s arrangement became fully apparent: through centralising the processes involved with decentralising control over the healthcare reform agenda, the agency articulates individual stakeholders’ and networks’ goals with those of other stakeholders and networks, as well as with State-wide system-level and science-driven reform agendas in the interest of nurturing alternative perspectives on care innovation and better outcomes and experiences for patients.” (Iedema et al 2017)*  **“***sharing lessons was a key feature of all team meetings. Learning from one another was a priority, alongside taking time to work together to solve problems. Program improvements were determined collectively by the network through regular reporting on network-wide program use and continuous quality improvement efforts” (Sibbald et al 2020)*  **“***At a time of significant upheaval for the RHAs – they were in the throes of amalgamating into AHS – a small group of independent, lone, long-term generalists working in a field technically and culturally peripheral to what was seen as core healthcare operations, began talking to one another and directly to politicians. Out of these LPCI conversations came a vision, and the technical details of the PCNs. As one policy-maker noted, the vision coalesced around local solutions for local problems. The local PCN basically got the dollars … in order to meet the needs of the local community.” (Leslie et al 2020)*  **“***PCIC was developed on the acknowledged need to share resources and garner support for quality improvement in primary care. PCIC participants all agreed that the need to work together was very strong and was the driving force behind their performance and success: “we figured out we could do more than what we were just doing. You know, we were brought around for something and it was just so exciting that it was like everybody came, the conversation never stopped, it just kind of, and it was like wow! We should make this bigger” (Provider FG#1). There was also an internal drive to share best practices coupled with an external pressure to provide better care in different ways. The creation of the PCIC Network played a critical role solidifying a shared vision for the respiratory health and gave the Lung Health Program overall legitimacy.” (Sibbald et al 2020)*  *“The different perspectives individuals and groups brought to the issue perpetuated professional and epistemic boundaries and these resulted in semantic boundaries. The geographic delineation of the CLAHRC and network of CLAHRCs resulted in physical and spatial boundaries…These conditions reduced the opportunity for interaction, communication and working together in general, and specifically, in implementation-related activity. Despite the call for collaboration embodied in the CLAHRC concept, in practice, participants’ reflections often represented different points along a co-operation to collaboration continuum.” (Rycroft-Malone et al 2017)* |
| 2C. When network members have common professional or vocational identities or calling (context) this facilitates the development of a collective network vision (outcome) because they are more likely to have common professional ideals/identities (mechanism) | **“***PCIC was developed on the acknowledged need to share resources and garner support for quality improvement in primary care. PCIC participants all agreed that the need to work together was very strong and was the driving force behind their performance and success: “we figured out we could do more than what we were just doing. You know, we were brought around for something and it was just so exciting that it was like everybody came, the conversation never stopped, it just kind of, and it was like wow! We should make this bigger” (Provider FG#1). There was also an internal drive to share best practices coupled with an external pressure to provide better care in different ways. The creation of the PCIC Network played a critical role solidifying a shared vision for the respiratory health and gave the Lung Health Program overall legitimacy.” (Sibbald et al 2020)*  *“The formation of clinician-led networks in NSW in 2002, was predicated on the principles of multidisciplinary membership, collaboration, and engagement in health system innovation [13]. Participants remarked that these principles had underpinned the development of strategic network project objectives focused on addressing local and statewide health system needs that, prior to the establishment of clinical networks, had not been satisfactorily addressed because of lack of multidisciplinary input: ‘What gets things up is the whole network package’. What this ‘package’ added to the pre-existing status quo, was a mechanism by which multi-disciplinary groups of clinicians could initiate and drive collaborative projects focused on quality improvement and improving patient outcomes related to their clinical specialty, using a ‘bottom-up’ push.” (McInnes et al 2015)*  *“Another participant from AHS repeated the mantra and emphasized, there has to be flexibility, because PCNs were created [to deliver] local solutions for local problems. (P7) In this way the independent professional and vocational identities of family physicians guided the initial vision for the PCNs. The technical details were also worked out in a culturally concordant manner.” (Leslie et al 2020)* |
| 2D. If the collective vision of a network is based on shared experiences, emotions, perspective, and understanding among the network members (a similar specific way in which reality is perceived) (context) then this will lead to commitment to the collective vision (outcome) because network members feel represented (mechanism) |  |
| 2E. If the collective vision of a network is communicated and explained by network leadership in a way that appeals to professional ideals and values shared by network members (context) then this will lead to commitment to the collective vision (outcome) because network members feel engaged (mechanism) | *"Getting hospital management to buy-in to the NOC structure was difficult at first because they feared losing income through outward referrals to lying-in clinics and had doubts about the quality of care possible at those sites. Persistent advocacy by QRP leadership eventually assured hospital administration buy-in by contending that rational distribution of cases would improve quality of services delivered and not result in income losses, demonstrating early signs of this success through presentation of QRP data and less formal anecdotal evidence." (Vergara et al 2020)*  *"The network was initiated with a meeting of a paediatrician, a senior nurse and the health records information officer from each participating hospital and the research institute and university partners. At this meeting and at subsequent hospital-specific introductory visits the network was explained and its purpose to promote better generation and use of health information to support better care.” (English et al 2017)*  “*The network’s vision provided a common foundation and a shared approach, allowing each member to work towards a collective goal. PCIC and ARGI participants described how the network leaders communicated the vision clearly and helped set goals collectively while continuously supporting individual network members and RTs: “… it’s a very goal-oriented group that looks for solutions and that I find, quite uplifting. Wednesday I’m feeling kind of low and scattered, but Friday (after our team meeting) a little more feeling good, I’ll feel energized, like we’ve got a direction.” (Provider FG#1). (Sibbald et al 2020)* |
| 2F. When formal agreements have been negotiated and agreed between network members, (context) then the network members will be more likely to follow a collective vision (outcome) because this helps network members to understand their roles and responsibilities (mechanism) | *“There should be an MOU between hub and shared-care centres, to strengthen the understanding of the teams and of hospital management. It should deﬁne responsibilities and timeframes. It is also much easier to achieve conformity and guidelines if hospital management are involved from the start of the programme.” (Burns et al 2018)*  *"As a central part of the collaboration, PIVOT developed strong relationships with the Government of Madagascar and with local communities in Ifanadiana. The foundation of the MoPH/PIVOT partnership is based on purposeful agreements that clarify common objectives and specify each party’s responsibilities; this clarity fosters an enabling environment for the NOC." (Cordier et al 2020)*  *"Representatives of all public and private health facilities worked collaboratively with district health officials and civil society representatives to map the most efficient referral pathways between puskesmas and hospitals. Roles and responsibilities of each entity in the referral system were outlined. Memoranda of understanding (MOU) formalized the referral networks by defining the governance of the network, ensuring that all entities within a referral network, including private sector providers, had clearly defined and agreed- upon roles and responsibilities. The MOU also served as a mechanism for holding all entities within the network accountable for upholding their roles and responsibilities. The MOUs were modified periodically as roles and responsibilities evolved within the network, such as facilities developing more capacity over time to manage complications." (Hyre et al 2019)* |
| 2G. When potential network members establish and agree to a collective vision (context) this purposefully links them into a network (outcome) because they have common understanding and perspectives on what the goals of the network are (mechanism) | *“The formation of clinician-led networks in NSW in 2002, was predicated on the principles of multidisciplinary membership, collaboration, and engagement in health system innovation [13]. Participants remarked that these principles had underpinned the development of strategic network project objectives focused on addressing local and statewide health system needs that, prior to the establishment of clinical networks, had not been satisfactorily addressed because of lack of multidisciplinary input: ‘What gets things up is the whole network package’. What this ‘package’ added to the pre-existing status quo, was a mechanism by which multi-disciplinary groups of clinicians could initiate and drive collaborative projects focused on quality improvement and improving patient outcomes related to their clinical specialty, using a ‘bottom-up’ push.” (McInnes et al 2015)*  *“Collaborative relationships between DLHs and RHs around a shared objective of providing optimal patient care are of great importance in determining the referral network’s overall performance, as the components of the system are mutually reliant on each other and share reciprocal relationships.” (Pittalis et al 2021)*  *"Coordinated action was driven by shared goals, recognition of inter-dependence, and greater shared responsibility: We all share the same goal and it’s kind of motivating. (Dietitian) It is like a link, everyone is linking with the other so everyone is playing his or her role, that’s what I can say … We are working like this because the one can’t survive without the other. (Hospital manager)" (Schneider et al 2020)*  *"The structures and processes of the MRU were specifically designed to enable coordinated action across key interfaces. These interfaces were between the PHC system players and district hospitals at the sub-district level, between district and sub-district managers, between units within hospitals, and between clinicians (experts) and managers (drivers). The new relationships and common collective visions for MNCH forged across these interfaces were generally regarded as the most important organizational impacts of the MRU.*  *…what I’ve found the system able to do was to break down those silos and to cross-pollinate across the whole district, and I find that extremely helpful. (DCST member)*  *Before, it was like two entities, the hospital and the clinics. But I don’t know what forces or whatever it has combined us now, we are not two entities anymore. (PHC manager)" (Schneider et al 2020)* |
| 2H. If there is no collective vision or if network members do not follow the network’s collective vision (context) then they will be less successful in implementing network activities (outcome) because network members do not feel committed to the network (mechanism) | *“Less successful and effective networks were seen by all stakeholders as being less well organised and structured and as being run by ‘turfdom and fiefdoms’ focused on objectives of individual interest rather than of broader relevance to the health system: “Trying to protect their particular practice and fear that they’re going to lose something. Rather than seeing the gains for everybody else in the state.” (McInnes et al 2012)*  *“Our findings demonstrate that when beliefs, values and priorities among members of the referral network converge, this fosters cooperative behaviours which are beneficial for individuals and the network as a whole. When values, beliefs and priorities diverge, self-interest prevails to the benefit of individual clinicians but impacting negatively on other clinicians and potentially on patients.” (Pittalis et al 2021)*  *"However, we did note resistance among nurses to collecting data for CIN. Paediatrician 12 noted: “Some [nurses] … tick information and … I feel like probably they didn't do it.” Nurses explained that they were often too busy focusing on clinical care to document data: “The workload is just too much … [Nurses] don't have time … observations are not well documented … [nurses] concentrate on giving treatment or attending emergencies” (Nurse-in-Charge 14). Some CIN members believed such resistance required further pastoral intervention. For example, HRIO 26 noted that CIN's “biggest challenge” was clinical staﬀ who “don't understand the purpose or the beneﬁts of data” and so provided “data which is inaccurate” or “incomplete”. The HRIO added that these clinical staﬀ needed to understand that this was not “good for decision-making and that's why the country is not moving ahead.” (McGivern et al 2017)* |

### Taking action to solve a problem

| 3A. In a bottom-up network when potential network members realise they alone are unlikely to be able to solve a problem (context), they will recruit likeminded colleagues for their cause (outcome) because they believe collective action is needed (mechanism) | *“PCIC was developed on the acknowledged need to share resources and garner support for quality improvement in primary care. PCIC participants all agreed that the need to work together was very strong and was the driving force behind their performance and success: “we figured out we could do more than what we were just doing. You know, we were brought around for something and it was just so exciting that it was like everybody came, the conversation never stopped, it just kind of, and it was like wow! We should make this bigger” (Provider FG#1). There was also an internal drive to share best practices coupled with an external pressure to provide better care in different ways. The creation of the PCIC Network played a critical role solidifying a shared vision for the respiratory health and gave the Lung Health Program overall legitimacy.” (Sibbald et al 2020)* |
| --- | --- |
| 3B. While in a top-down/more directed network when health systems administrators or managers realise that they need diverse health system actors to be able to solve the problem/meet the target (context), they will recruit/mandate/encourage colleagues they perceive to be capable to their cause (outcome) because they believe that collective effort/action is needed (mechanism) | *“During the course of the visits to the districts, sub- districts and facilities, the facilitator was also actively engaged in a process of communication and collective sense-making, building consensus on firstly, the problem to be prioritized (high MNC mortality) and secondly, a set of appropriate responses to the identified problem. The latter entailed presenting the evidence base on interventions for MNCH, drawing attention to existing guidelines, and packaging formal information in ways that promoted system thinking and enabled individual players to locate their place in the whole.” (Schneider et al 2020)*  *“The general point that can be made about this is that the HIPS initiative acted as a forum for the articulation of top-down direction and local opinion. On the one hand, the taskforce was charged with a policy reform agenda that aimed to reduce clinical practice variation. On the other hand, its decision to target hip fracture care arose through open deliberation among taskforce members. Seen through the lens of network governance, the taskforce facilitated deliberations among clinicians, academics, bureaucrats and service users about where to direct its energies, and how to tackle the issue identified as needing attention, resulting in a reform agreement (the minimum standards) as the basis for a state-wide ortho-geriatric improvement initiative.” (Iedema et al 2017)* |
| 3C. When potential network stakeholders think collective action is needed to solve the identified problem (context), they will seek out other individuals with common experience or perspectives (outcome), because they believe such people may be willing to help them (mechanism) | *"In 2013, in the face of the ongoing challenge to reduce maternal death, the head of the Obstetrics and Gynecology (Ob/Gyn) Department at QMMC decided to take a proactive approach by designing a program to decongest the maternity ward with the aim of improving quality of care and outcomes. The Quirino Recognized Partners (QRP), as a Network of Care, identified public and private lying-in clinics within first a seven, then a ten-kilometer, radius around the hospital. These were identified as potential “spokes” in a hub-and-spoke model. Identifying clinical sites to partner with was the first step in the process of creating a group of intentionally interconnected maternity care touch points within a defined catchment area. This group, the nascent Network of Care, defined common goals while distributing maternity cases rationally among the facilities and to achieve better outcomes for the women and newborns in their care. QRP is a public–private partnership, uniting the public hospital, some public health centers, and a host of private birth centers run by entrepreneurial midwives. Together, the partners distribute maternity cases by risk profile and location, communicate fluidly, and share monitoring information to track performance of the Network of Care.”* *(Vergara et al 2020)*  *“Individual DLHs also use the referral network to exchange resources and cope with challenging situations. For example, respondents reported that Mulanje DLH refers patients to the nearby Mulanje mission hospital in case of (frequent) shortage of essential resources, but occasionally also “borrows” the mission hospital’s more experienced clinicians to manage particularly difficult cases locally and avoid referral. A number of other DLHs reported having informal local arrangements with each other allowing them to move their whole surgical team to perform urgent operations at nearby facilities when there are major failures in equipment or interruptions to electricity or water supply. Others share supplies and other surgical essentials, such as instrument sets or gowns.” (Pittalis et al 2021)*  *‘‘On our last visit to Baglung District, we had to drive 6 h from Kathmandu over a bumpy, winding dirt road, across rivers … you know, remote. When we finally got up to one particular village in the mountains, the senior auxiliary health worker came out to meet us. He began to tell us about how our program was working. He knew all the statistics about who delivered and how many delivered, and all the progress they had made. He said that as a leader in the community he established a no-home births policy. ‘No women should deliver at home, and no one should deliver alone,’ he said. He went around to every household in the community and collected money and with that money he built a birthing center. This was probably also related to the fact that our organization had promised to supply the equipment for the center. Another man in the village donated his land for the building site. He was not wealthy. Together the community built this space. It was one of the best I have ever seen: well constructed, clean, organized, attached bathroom, a separate room for intake and prenatal care, a room for labor and delivery, and a room for postpartum care. All of the decisions and effort that created this facility were initiated by the men in this community. Not just the raising of the money and setting the policy, but the community outreach as well. The district health officer was a man. The head of the hospital was a man. Most health post supervisors were men. The person who donated the land was a man. In many ways it is always men who are decision-makers about health care for women. So, of course, men will be the driving force for change.’’ (Adams et al 2016)* |
| 3D. When members in an established network have access to resources that will help them solve the problem they have identified (context), they may be more prepared/more likely to intend to take action (outcome), because believe they have a more realistic chance of success (mechanism) | *"In Kenya, health professionals often work in remote district hospitals, overseeing clinical departments with little support or training. Many find this difficult, particularly without resources to provide high standards of care. Consequently, loss of motivation and burnout are common (Brown, 2016; Mbindyo et al., 2009). CIN leaders attempted to create a supportive network community to addressed this problem, by providing physical and online spaces in which to meet and share as a professional community. CIN encourages network participants to think about new approaches to problem-solving, and then implement, test and measure solutions, and facilitates sharing of learning across participating organizations, thus functioning as an improvement collaborative (English, 2013)." (McGivern 2017)*  *"To help develop ‘engaged and motivated’ members of CIN at local levels and build a collective identity and community of practice, we hold twice yearly face-to-face meetings with paediatricians inviting the other CIN focal persons to one annual meeting.9 These meetings allow participants to discuss their audit reports (including offering suggestions for improving data collection) and to identify underlying problems and potential solutions with peers. They also provide a forum for short, specific skills building sessions focused on the ‘soft skills’ needed as a manager (eg, how to run a team) and on building their understanding of research. Crosstalk among the scientific, clinical and policy communities in an arena promoting collegial relationships helps interpret results based on an understanding of practice in the real world. This enables all sides to understand how contexts interact with improvement approaches to bring about observed outcomes26" (Irimu et al 2018)*  “*The network’s vision provided a common foundation and a shared approach, allowing each member to work towards a collective goal. PCIC and ARGI participants described how the network leaders communicated the vision clearly and helped set goals collectively while continuously supporting individual network members and RTs: “… it’s a very goal-oriented group that looks for solutions and that I find, quite uplifting. Wednesday I’m feeling kind of low and scattered, but Friday (after our team meeting) a little more feeling good, I’ll feel energized, like we’ve got a direction.” (Provider FG#1). (Sibbald et al 2020)* |

### Forming purposeful relationships, linkages, and partnerships

| 4A. When network members believe in the network’s collective vision (context) then this can help ensure that network members have purposeful and co-operative working relationships (outcome) because they are more willing to work and value working with people whom they identify as being likeminded (mechanism) | *"The structures and processes of the MRU were specifically designed to enable coordinated action across key interfaces. These interfaces were between the PHC system players and district hospitals at the sub-district level, between district and sub-district managers, between units within hospitals, and between clinicians (experts) and managers (drivers). The new relationships and common collective visions for MNCH forged across these interfaces were generally regarded as the most important organizational impacts of the MRU.*  *…what I’ve found the system able to do was to break down those silos and to cross-pollinate across the whole district, and I find that extremely helpful. (DCST member)*  *Before, it was like two entities, the hospital and the clinics. But I don’t know what forces or whatever it has combined us now, we are not two entities anymore. (PHC manager)" (Schneider et al 2020)* |
| --- | --- |
| 4B. When network members are open to and able to invest time in developing relationships and linkages through the network (context) then this can help ensure that network members have purposeful and co-operative working relationships (outcome) because they have a better understanding of each other (mechanism) | “*Interview respondents highlighted that particular DLHs have established positive working relationships with RHs over the years to the benefit of both parties. This is often driven by the spirit of initiative of individual district clinicians, who have invested time and efforts in cultivating collaborative relationships with RH specialists, and persistence to maintain them. As explained by a RH respondent when describing one such clinician: “he is very collegial. All the time, if [name of district clinician] sends something [a case] you know for sure that’s something which he really needed help with.[...] If [he] was behaving like all the other ones he would have been a tough guy, giving us all the problems, but he is very professional and he communicates very well” (27RMW).” (Pittalis et al 2021)* |
| 4C. If network members have strong pre-existing relationships (context) then this helps ensure that network members have purposeful and co-operative working relationships (outcome) because they are already familiar with each other (mechanism) | *“However, a lack of interest in participating also emerged in all five countries, with greater intensity in Uruguay and Brazil in general; and with regard to offline virtual consultations in Mexico, and replica meetings (meetings with other network doctors not in the PP) in Colombia. Contributing factors, according to interviewees, were: the ‘limited adherence to the primary care-based model’ of some SC doctors in Brazil, Chile and Uruguay and ‘not knowing the doctors of the other care level’, ‘mutual mistrust’ and PC doctors’ ‘shyness and fear to express their doubts’ in Chile, Colombia and Mexico. However, these obstacles diminished as they participated: Yes, yes, at the start I found it really difficult to participate (.. .) at first it was like: ‘um...what am I going to do there? They’re all going to be looking at me, I’ve got to give a reply, it’s scary, I don’t want to do this’ (.. .). But it’s all got a lot better, (...) they want to participate, because they’re enjoying it. (Administrative personnel, Chile)” (Vargas et al 2 2020)*  *“In contexts where there was an absence of a history of established relationships and collaboration, where there has been less activity around the joint setting of priorities either at bid development stage, the need to sell the benefits of CLAHRC to encourage or incentivise engagement of mutual benefit was evident: I think that getting people engaged in it is about them seeing there’s some mutual benefit and where there wasn’t seen as any mutual benefit it doesn’t happen…I know other CLAHRC Directors feel a bit the same, that you feel like you’re a salesperson going round trying to sell things. (Leader, Hazeldean)” (Rycroft-Malone et al 2017)* |
| 4D. If there are artefacts in place that outline the roles and responsibilities of network members (context) then this can help ensure that network members have purposeful and co-operative working relationships (outcome) because there is a common understanding among network members about what they are expected to do (mechanism) | *“Ensuring that a true partnership exists among project staff, service providers, the government, donors, and other key state stakeholders is crucial to a successful pooled effort. Clearly outlining what each organization is expected to provide or do and ensuring that contributions come from the state and local governments and other local stakeholders has resulted in continued successes.” (Fistula Care 2010)*  *"As a central part of the collaboration, PIVOT developed strong relationships with the Government of Madagascar and with local communities in Ifanadiana. The foundation of the MoPH/PIVOT partnership is based on purposeful agreements that clarify common objectives and specify each party’s responsibilities; this clarity fosters an enabling environment for the NOC." (Cordier et al 2020)*  *“There should be an MOU between hub and shared-care centres, to strengthen the understanding of the teams and of hospital management. It should deﬁne responsibilities and timeframes. It is also much easier to achieve conformity and guidelines if hospital management are involved from the start of the programme.” (Burns et al 2018)*  *“Some from the senior health service manager and policy-maker groups, expressed that there were some tensions in the evolution of this relationship and this was said to be a barrier to network development. It was felt that not all network personnel effectively liaised with the state health department. This was largely attributed to a lack of understanding of the correct procedures and processes for consulting and communicating with government. Developing strong positive relationships between networks and senior administrators and executives from local health services were regarded as essential by all stakeholder groups. However, the development of this relationship was seen as a ‘work in progress’. From the perspective of those in the network group, this was partially attributable to local health services reluctance for network initiatives to extend across health service boundaries: “This is part of the local health service culture - they see all health needs being able to be met within their boundaries and by the health service employee.”” (McInnes et al 2012)*  *“Fostered clinical agreement on treatments, diagnostic tests and referral criteria “on going over the meeting topics and all that, well let’s just say that we get a space in which to air our differences or opinions, which us general doctors also have on the management of cases (…). So there are discussions, there’s a space for discussion and so somehow more agreements are reached” (PP, Colombia). “(…) I think the most positive aspect [of the videoconferences] is about coming to an agreement on the type of pathology we’re working on, you know? Through a protocol, so that each one of us understands what they have to do and what their responsibilities are” (Healthcare professional, Chile)” (Vargas et al 2020)* |
| 4E. When a network creates opportunities for relationship building between network members (context) this may help in the creation of purposeful and co-operative working relationships and improve existing working relationships (outcome) because they get to know each other’s personality, skills, ways of working, and motivations better (mechanism) | *“The informants perceived that joint training meetings had an impact on interactional factors, since personal knowledge between physicians was allowed; thus, collaboration was favoured: “This close relationship among people working here is fostered, because we no longer perceive them so distant…we perceive them as members of the same team…attitude towards the patient changes when you know that he is received by someone you know” (Professional Platform/Primary Care).” (Lopez-Vazquez et al 2021)*  *“Regarding the training method, informants considered that the open attitude and absence of hierarchies shown by facilitators of the joint training meetings on maternal health, as well as including practical activities and resolution of clinical cases, enhanced dialogue, interest and involvement in the sessions. Thus, the initial defensiveness of primary care physicians was eliminated, a climate of equality (Table 4d) was fostered, and the resolution of doubts via direct communication between peers was promoted: “Now, I was able to stay in touch with a gynaecologist, and any particular doubt I could not solve by reading (regarding CPG), so I could trust them, I was able to detect a greater close relationship” (Professional Platform/Primary Care).” (Lopez-Vazquez et al 2021)* |
| 4F. When network members have established purposeful relationships (context) this helps to improve communication between network members (outcome) because they are familiar with each other (mechanism) | *“These new relationships facilitated more open day-today communication: There is a lot more open communication. If there is an ANC that you must refer now it is high risk, you can phone up to the maternity ward. (PHC manager). It is easy to pick up a phone and phone them and ask the sister to tell me about this patient. Most likely they will be able to tell you about the patient because they draw files. So we have a very good working relationship with the guys outside especially the PHCs. (Pediatric ward manager)." (Schneider et al 2020)*  *“Allowed clinical cases on the waiting list or complex cases to be resolved in primary care “(..) it was shown to be useful [joint virtual clinical conferences],it’s very useful, for example when the general doctor who has a grandma with dementia that wakes everyone up at 3 in the morning, so he doesn’t just have one patient now but 5, which is the whole family that hasn’t slept… and we [SC doctors] can say to him: ‘well let’s have a look at this, or that, we’ll treat it with this medicine, we’ll do these tests, and it’s not necessary for her to come over here’” (Healthcare professional, Chile) (Vargas et al 2020)*  *“Most informants in Chile pointed out that as a consequence of the virtual joint meetings, agreement was reached on how to manage or resolve clinical cases that were complex or on the waiting list, thus avoiding referrals to specialist care. In Colombia and Mexico, they also highlighted solving queries and reaching general clinical agreements on disease management, not only for specific cases: “(...) so the fact that, for example, I can now talk to the dermatologist and say “Look, I’ve got this case...” Before the videoconference that just didn’t exist and was unthinkable. So, now I’ve resolved a couple of cases that were really complicated, and there was a waiting list of I don’t know how many years (…) so in that sense it’s been really favourable (…)” (PC doctor, Chile).” (Vargas et al 2020)*  *“Impact limited to the specialities that participated, or little impact on coordination “Well yes I think so, in the areas we had contact with. Of course the hospital is huge, there’s a lot of different specialities that also need the chance to get to know each other and that’s the main reservation I have, that one person can only do so much (…) So that’s why I think it may be important to repeat it [cross-level visits] to cover more specialities” (Administrative professional, Chile) “The relationship between general doctor and the specialist that was directing, leading the progress of the meeting (…) was great because they talked to each other, communicated, they got to ask each other questions and they used tools like WhatsApp text messages.” (Vargas et al 2020)* |
| 4G. When purposeful linkages and strong working relationships are established between network members or between network members and external stakeholders (context) this bridges gaps between network members or network members and stakeholders (outcome) because of mutual understanding (mechanism) | *"To help develop ‘engaged and motivated’ members of CIN at local levels and build a collective identity and community of practice, we hold twice yearly face-to-face meetings with paediatricians inviting the other CIN focal persons to one annual meeting.9 These meetings allow participants to discuss their audit reports (including offering suggestions for improving data collection) and to identify underlying problems and potential solutions with peers. They also provide a forum for short, specific skills building sessions focused on the ‘soft skills’ needed as a manager (eg, how to run a team) and on building their understanding of research. Crosstalk among the scientific, clinical and policy communities in an arena promoting collegial relationships helps interpret results based on an understanding of practice in the real world. This enables all sides to understand how contexts interact with improvement approaches to bring about observed outcomes26" (Irimu et al 2018)*  *"Our data support the value of formally recognizing and integrating TBAs into EONC services, consistent with observations that when integrated into a healthcare system, TBAs can play an integral role in improving EONC services and maternal and newborn health (13–15). Moreover, through the creation of an EONC network, the project bridged profound gaps among fractured and isolated public and non-governmental institutions aligning formal and informal health systems and linking science-based health care with traditional and culturally responsive procedures. EONC was tailored to be implemented in a three-level network of services – parish health centers, district, and provincial hospitals – each one with its specific responsibilities and an effective referral system. QI methods were implemented at each level in the EONC network. Application of these principles resulted in an increase of access to and quality of EONC services. It greatly contributed to improving postpartum and neonatal care within the first 2 days of delivery, an outcome that likely contributed to neonatal survival improvement" (Broughton et al 2016)*  *“Networks that had not formed strategic external relationships were thought by some participants to have limited reach and to experience difficulties in facilitating projects that could deliver significant impacts: They [the network] do have a strong voice with local hospitals, but not really beyond. It is the relationships with external bodies (professional, academic and health departments) that helps to facilitate and implement projects that will make the bigger impact. Ability to effectively engage with external bodies relevant to the network such as government; professional organisations and colleges, was therefore seen as important for maximising network potential to achieve significant long-term impacts.” (McInnes et al 2015)*  *“Partnerships with external stakeholders, such as the state government health department and the local health services, were regarded as an important and desirable outcome by all stakeholder groups, not least because it was seen as giving networks credibility and legitimacy in the broader health arena. Participants stated that since the formation of clinical networks, overall the relationship between clinicians and government agencies had improved and had led to greater mutual understanding of perspectives: “I think the successful outcomes have been the partnerships and how those partnerships have worked in an open and transparent way and that’s how we need to do it. At a hospital level, at a local health service level, at a state level and at a national level.”” (McInnes et al 2012)*  *"Generating buy-in from, and establishing trusting relationships with, these public sector partners was the first step in developing the network. The process began with the initial needs assessment and continued throughout proposal writing, facility selection and implementation." (D’mello et al 2020)*  *“Referral protocols should include alternatives outside the referral network sites. Of all referrals, 65% were sent to the referral hospital inside the MRN, while 30% were sent to an out-of-MRN hospital. For the out-of-MRN sites, often private or NGO hospitals, providers reported that they believed patients with particularly complicated cases would receive better care. The study found that establishing relationships with hospitals that are commonly used but are not formally part of the referral system is important to strengthening the network plan. These relationships are especially important during hospital strikes, when services may be lacking or nonexistent at certain public hospitals.” (MCSP Haiti 2018)* |

### Network leadership

| 5A. When networks have a member(s) that clearly take on a leadership role and focus on building linkages between potential network members and stakeholders and create communication channels (context) this helps the network to form (outcome) because it brings people together (mechanism) | *“Participants were unanimous about the critical importance of the role of the network manager in contributing to network success. Participants from the high- and moderate- impact networks frequently attributed the success of their network to the work and skills of the network manager. As well as project management, communication, organisational, coordination and leadership skills network managers also needed high credibility, be able ‘to talk the language of networks’, that is to communicate the vision and principles of networks to a range of clinical and policy stakeholders, and to have good interpersonal skills. To lay the groundwork for later success the network manager had to ‘hit the ground running’ in the early stages of a network’s development. One participant attributed the success of their network to the activities of their network manager in the early stages of the network’s history: They built links; met with all key players and created formal and informal communication channels between diverse bodies. Effective network managers also: Provided a hub around the network’s clinical specialty and engaged with key stakeholders and the broader clinical base formally and informally.” (McInnes et al 2015)*  *“For network chairs (and network managers) skills in negotiation and relationship building were regarded as essential by all participants. These skills were thought to bring the ‘outliers into the fold’ (‘outliers’ referring to those not involved in networks) and to enable connection with key partners in academic, professional and policy spheres, thus ‘building a broad base of support from which to facilitate achievements and significant impacts’.” (McInnes et al 2015)*  *“The essence of effective network leadership appeared to hinge on the ability of the network chairs and network managers to span both discipline and sector boundaries and to champion network agendas and associated projects, linking networks to key stakeholder organisations such as professional colleges and policy agencies to ensure buy-in and the spread of network innovations.” (McInnes et al 2015)*  *“In our study, charismatic and visionary network chairs who could negotiate with the Ministry of Health, particularly in the early stages of a network’s history, were perceived as kick-starting the network on the road to success. These individuals had attributes indicative of a transformational style of leadership [17]. These attributes included possessing knowledge of complex systems, ability to influence peers and to bring together diverse groups of people across disciplinary and organisational boundaries to form positive and productive alliances. These types of clinical leaders have been termed ‘strategic clinicians’ [15] and represent clinicians who ‘think more managerially and strategically’ [15]. However it was also noted that those networks who mainly depended on a network chair to be the primary driving force, risk having limited impacts.” (McInnes et al 2015)*  *"In the sub-units where the MRU did not convene (except when visited by the external facilitator), this was usually due to a gap in leadership and support from either the hospital or PHC services, preventing the establishment of the informal coalition of actors" (Schneider et al 2020)* |
| --- | --- |
| 5B. When networks have a member(s) that clearly take on a leadership role and communicates the network’s vision and helps to set collective goals (context) this helps to bring network members around the collective vision and to move forward in the same direction (outcome) because they have the same understanding (mechanism) | “*The network’s vision provided a common foundation and a shared approach, allowing each member to work towards a collective goal. PCIC and ARGI participants described how the network leaders communicated the vision clearly and helped set goals collectively while continuously supporting individual network members and RTs: “… it’s a very goal-oriented group that looks for solutions and that I find, quite uplifting. Wednesday I’m feeling kind of low and scattered, but Friday (after our team meeting) a little more feeling good, I’ll feel energized, like we’ve got a direction.” (Provider FG#1). For network members, this translated back to their primary goal of delivering high quality patient care.” (Sibbald et al 2020)*  *“There were two unique aspects of this role that were believed by participants to lay the groundwork for success. One was the ability to ‘clear a path through the maze of issues’. This referred to the ability to anticipate and manage operational and project complexities and to negotiate with and involve the right stakeholders at the right time in relation to critical project stages. The other unique aspect was the ability to effectively translate information and ideas to different audiences, namely to clinicians, funders and policy makers. In effect, acting as a knowledge-broker able to ‘deliver high content knowledge’ to a wide audience and to: Sell the project – make internal and external stakeholders feel as though the network initiative is an important, achievable target.” (McInnes et al 2015)*  *“Participants were unanimous about the critical importance of the role of the network manager in contributing to network success. Participants from the high- and moderate- impact networks frequently attributed the success of their network to the work and skills of the network manager. As well as project management, communication, organisational, coordination and leadership skills network managers also needed high credibility, be able ‘to talk the language of networks’, that is to communicate the vision and principles of networks to a range of clinical and policy stakeholders, and to have good interpersonal skills. To lay the groundwork for later success the network manager had to ‘hit the ground running’ in the early stages of a network’s development. One participant attributed the success of their network to the activities of their network manager in the early stages of the network’s history: They built links; met with all key players and created formal and informal communication channels between diverse bodies. Effective network managers also: Provided a hub around the network’s clinical specialty and engaged with key stakeholders and the broader clinical base formally and informally.” (McInnes et al 2015)*  *"The network was initiated with a meeting of a paediatrician, a senior nurse and the health records information officer from each participating hospital and the research institute and university partners. At this meeting and at subsequent hospital-specific introductory visits the network was explained and its purpose to promote better generation and use of health information to support better care. Collaboration is supported from the network centre by a clinical coordinator who coordinates network meetings and offers the feedback, discusses it and provides advice as required while also promoting peer-to-peer support. During the 4–6 monthly meetings predominantly with paediatricians specific short sessions (< half a day) were provided that explained leadership of teams, how to give group feedback, on understanding complex systems and on the principles of quality indicators and their use. The training typically used discussion, reflection and individuals’ experiences as well as presentations. Relationships and the educational approach were complemented by visits to hospitals by the clinical coordinator in the first year to explain and discuss the hospital-specific indicators provided in an overall report National guidelines for care were distributed to network and non-network hospitals as part of a national distribution and some network paediatricians took part in updating these guidelines in 2015" (English et al 2017)* |
| 5C. When networks have a member(s) that clearly take on a leadership role and actively coordinates network members (context) this helps the network to form (outcome) because there is a central figure bringing people together (mechanism) | *“Collaboration is supported from the network centre by a clinical coordinator who coordinates network meetings and offers the feedback, discusses it and provides advice as required while also promoting peer-to-peer support. During the 4–6 monthly meetings predominantly with paediatricians specific short sessions (< half a day) were provided that explained leadership of teams, how to give group feedback, on understanding complex systems and on the principles of quality indicators and their use. The training typically used discussion, reflection and individuals’ experiences as well as presentations. Relationships and the educational approach were complemented by visits to hospitals by the clinical coordinator in the first year to explain and discuss the hospital-specific indicators provided in an overall report National guidelines for care were distributed to network and non-network hospitals as part of a national distribution and some network paediatricians took part in updating these guidelines in 2015.” (English et al 2017)*  *“Another diversifying factor was that network members were drawn from the various clinical, patient, academic and non-government organisation communities across metropolitan, regional and rural areas. Members were regularly invited to attend network meetings and events in person or through video-conferencing. ACI-based network managers were central to building and maintaining their network’s dynamics and activities, and to achieving and coordinating network output and impact. At the same time, ACI had not made it a priority to standardise networks’ memberships, their deliberative dynamics, or their output. This attests of ACI’s tolerance towards variability and acknowledgement of the influence exerted by individual members’ personalities, contextual pressures and constraints, and unexpected opportunities and unpredictable challenges.” (Iedema et al 2017)*  *“All stakeholder groups saw leadership across three different levels as an essential building block for effective structure, organisation and governance, and as a major facilitator of network effectiveness and success. Having ‘a strong network manager who can direct a lot of the stuff’ with ability to effectively liaise with clinicians, consumers, and external stakeholders; implement workplans; and effectively run network operations was one level of leadership which was seen as important, particularly for setting up organisational and governance processes. A participant from the network driver group summed up the importance of the network manager’s role as follows: “It’s a big jigsaw puzzle and you have to have one person, I think, who knows all the pieces of the puzzle.”” (McInnes et al 2012)* |
| 5D. When networks have a member(s) that clearly take on a leadership role and provide support and feedback to network members (context) this can help the network function and take more appropriate action towards the identified problem (outcome) because network members are engaged and see what they need to do to help achieve the vision (mechanism) | *"Normalizing data collection and clinical audit involved pastoral inscription practices too. CIN provided on-going training for data clerks, HRIOs and clinical teams about how to collect data and understand CIN quality care reports (English et al., 2017). CIN leaders provided performance feedback to participating hospitals in person for the ﬁrst 18 months, to ensure that participants could interpret data and use it to address problems, improve practices and clinical outcomes (English, 2013; English et al., 2011, 2017). Nurse-in-charge 30 commented that CIN's leaders: “keep on giving feedback … they come in [to district hospital] physically … to instill more conﬁdence and more value to the program. I think it is very useful.”" (McGivern et al 2017)*  *“Collaboration is supported from the network centre by a clinical coordinator who coordinates network meetings and offers the feedback, discusses it and provides advice as required while also promoting peer-to-peer support. During the 4–6 monthly meetings predominantly with paediatricians specific short sessions (< half a day) were provided that explained leadership of teams, how to give group feedback, on understanding complex systems and on the principles of quality indicators and their use. The training typically used discussion, reflection and individuals’ experiences as well as presentations. Relationships and the educational approach were complemented by visits to hospitals by the clinical coordinator in the first year to explain and discuss the hospital-specific indicators provided in an overall report National guidelines for care were distributed to network and non-network hospitals as part of a national distribution and some network paediatricians took part in updating these guidelines in 2015."” (English et al 2017)* |
| 5E. When networks have a member(s) that take on a leadership role and gets things done (context) network members develop greater commitment, engagement, and/or motivation (outcome) because they believe belonging to the network is worthwhile (mechanism) | *“There were two unique aspects of this role that were believed by participants to lay the groundwork for success. One was the ability to ‘clear a path through the maze of issues’. This referred to the ability to anticipate and manage operational and project complexities and to negotiate with and involve the right stakeholders at the right time in relation to critical project stages. The other unique aspect was the ability to effectively translate information and ideas to different audiences, namely to clinicians, funders and policy makers. In effect, acting as a knowledge-broker able to ‘deliver high content knowledge’ to a wide audience and to: Sell the project – make internal and external stakeholders feel as though the network initiative is an important, achievable target.” (McInnes et al 2015)*  *“All stakeholder groups saw leadership across three different levels as an essential building block for effective structure, organisation and governance, and as a major facilitator of network effectiveness and success. Having ‘a strong network manager who can direct a lot of the stuff’ with ability to effectively liaise with clinicians, consumers, and external stakeholders; implement workplans; and effectively run network operations was one level of leadership which was seen as important, particularly for setting up organisational and governance processes. A participant from the network driver group summed up the importance of the network manager’s role as follows: “It’s a big jigsaw puzzle and you have to have one person, I think, who knows all the pieces of the puzzle.”” (McInnes et al 2012)*  *“the Clinical Network Executive. This body was seen by all groups as having the authority and credibility to ‘take management issues up the chain’ to state government and as having a direct line to both the Minister of Health and the Director-General of NSW Health. From the perspective of those in the network driver group, maintenance of that direct line was seen as critical to the ongoing sustainability of networks. Some from this group commented that having a co-ordinating body such as the Clinical Network Executive helped to ‘keep [networks] going and on track’: “The support from the Executive is very, very important and executive understanding of the issues is even more important. I don’t think that we could undertake our projects without [the Executive Officer] who really is on top of all the issues and knows the ways to address them.”” (McInnes et al 2012)*  *“The ability of the network chair to engage the multidisciplinary clinical workforce in network initiatives, collaborate with external stakeholders, and to drive the implementation of network objectives was seen as critical for providing the foundation for the delivery of high-impact projects and creating a momentum towards successful network outcomes.” (McInnes et al 2015)* |
| 5F. When networks have a member(s) that take on a leadership role and dedicate time to setting up and the functioning of the network (context) network members develop greater commitment, engagement, and/or motivation (outcome) because they believe belonging to the network is worthwhile (mechanism) | *“The ‘institutional support’ of network managers emerged in all five countries as the main organizational factor that influenced implementation of the interventions (Supplementary Box S2). In Chile, and in Brazil with the occasional interruption, it was identified as a facilitator that remained stable throughout the process. In Mexico, it was strengthened following political change; in Colombia, it was weakened; and in Uruguay, it remained frail. It was considered key in terms of whether the resources needed (professionals’ time and materials) were available to implement the interventions (and in Brazil and Mexico also to solve operational problems—see Supplementary Box S2): ‘...there was support from the managers to protect these (allocated project) schedules, that normally doesn’t happen with this kind of more low-scale project’ (Professionals’ inter-level working group, Chile)” (Vargas et al 2 2020)* |
| 5G. When networks have a member(s) that clearly take on a leadership role and actively engage network members (context) network members develop greater commitment, engagement, and/or motivation (outcome) because network members feel part of the network (mechanism) | *"Developing Kenyan national guidelines involved extensive engagement with key stakeholders, including the Kenyan Ministry of Health, Kenyan Medical Schools and the Kenyan Paediatric Association. CIN Director 25 noted: “That process of developing the national guide … introduced me to … stakeholders … that needed to be engaged … [who] began to know me and what I was up to.” Thus, involvement in constructive practices indirectly established CIN leaders' expert and legitimate pastoral status. Yet this pastoral status was also grounded in experience of the challenges Kenyan clinicians faced. Both CIN leaders had practised in the Kenyan health systems for over 20 years and taught Paediatrics and Child Health at a leading Kenyan university, so knew many Kenyan paediatricians personally. Thus, CIN leaders had expert, professional and personal credibility." (McGivern et al 2017)*  *“The ability of the network chair to engage the multidisciplinary clinical workforce in network initiatives, collaborate with external stakeholders, and to drive the implementation of network objectives was seen as critical for providing the foundation for the delivery of high-impact projects and creating a momentum towards successful network outcomes.” (McInnes et al 2015)*  *“Influential and passionate clinical leaders (who lead or chair a network) were also regarded as necessary to build effective networks. Characteristics of a good clinical leader included being well respected by the clinical community and as having ‘a bit of fire in their belly’ and ‘a fair bit of ambition’. The ability to keep people engaged and to influence a wide range of consumers and clinical stakeholders was identified by all groups as an essential part of developing an effective network: “You need to keep people’s passion; if you lose that you lose sight of everything.”” (McInnes et al 2012)* |
| 5H. When network leadership consistently and regularly support and provide resources to network members (context) this helps network members act to achieve the network’s collective vision (outcome) because the network members feel empowered (mechanism) | *"A third inscription practice was mentoring and supporting use of audit for QI as a form of professional development (English et al., 2017). Nurse-in-Charge 5 commented: “Support is very important … people to guide and giving the feedback … to help improve data … quality of care … mentoring… I ﬁnd it very helpful.” Likewise, Paediatrician 4 noted: “[CIN leaders] will give you an answer that actually opens up a whole road of possibilities … they're really good mentors.” Mentoring and support was also apparent during CIN meetings: “The mentoring and supervision is useful, especially from the network coordinator … you see a lot of engagement… during [the CIN] forum … between the members of the network, the clinicians and their mentors.”" (McGivern et al 2017)*  *"Moreover, senior paediatricians and nurses ‘in-charge’ locally mentored colleagues to adopt the practices CIN advocated. Paediatrician 4 commented: “I mentor a lot of doctors … I love paediatrics … I'm passionate about what I do, including even quality improvement… it's kind of catching.” Consequently, QI practices spread via a constellation of CIN members through the inscription practices we describe" (McGivern et al 2017)*  *“Such feedback reports and participation in the network have prompted greater adoption and use of the standard paediatric admission record form and, consequently, overall improvements in documentation of clinical characteristics.” (Tuti et al 2016)*  *"A senior paediatrician (the CIN clinical coordinator) also followed up the provision of reports with specific emails and phone calls (after 1–2 weeks) to the paediatricians highlighting specific areas of success and areas requiring continued improvement. These calls aimed to provide encouragement and support as part of building a sense of being within the network." (Gachau et al 2017)* |
| 5I. When network leadership consistently and regularly promotes, encourages, or champions practices or influences network members to take up certain practices (context) this helps network members to adopt these practices (outcome) because it allows network members to fulfil a latent desire to practice better (mechanism) | *"championing in CIN involved ‘inﬂuencing’ rather than imposing change. CIN Director 25 noted: “We … promote and encourage rather than say you have got to do it this way … put yourself in the shoes of the person you're trying to support or inﬂuence, so that you can appreciate their realities.” Similarly, Epidemiologist 23 noted: “within CIN, where you have paediatricians who are authorities in their ﬁeld, the type of leadership that they need is one of inﬂuencing, not teaching or directing what they should do … you can achieve signiﬁcant changes in care through inﬂuencing” (McGivern et al 2017)*  *"From data analysis, we noted three pastoral inscription practices in CIN inscribing paediatric guidelines into routine care. The ﬁrst involved CIN's leaders evangelically championing CIN's purpose as beneﬁtting network participants and patients. CIN Director 1 described: “Challenging mind-sets … becoming a champion, going out speaking.” CIN Director 25 was described as “the force behind what we are doing” and “our champion” (CIN Director 1). Thus, by championing CIN's activities, its leaders motivated doctors and nurses involved in the network to use guidelines, clinical audit and data collection for QI purposes." (McGivern et al 2017)*  *“Another network participant from a well-established network, regarded the role modelling by senior clinicians of these new ways of collegial working as important an outcome as achieving improvements in patient outcomes: “Above all else, role modelling, so that younger clinicians can see that all disciplines are conversing, working together and can see that everyone has a place and a voice at the table. This is the most important, most desirable network outcome in my view.” (McInnes et al 2012)* |
| 5J. When network leadership create a welcoming and inclusive environment within the network (context) members feel more able to critically discuss issues and suggest possible solutions (outcome) because network members feel that they are in a psychological safe environment (mechanism) | *"Improving the quality of patient-level practice often requires support for an enabling professional culture. Providers at one of the CCBRT NOC referral hospitals noted that improvements in teamwork in labor and delivery resulted in a decline in their facility’s maternal and perinatal death rates. Further, they described how their team’s motivation was inspired largely by improved hospital management. When the medical officer in-charge and the hospital Matron walked around facilities to check-in with health workers, inquiring about maternity care and identifying how they could assist, it fostered a positive environment with improved teamwork. The clarification of a sense of purpose and confidence in leadership promoted by the NOC formation nurtured these and other better management practices." (D’mello et al 2020)*  *"A senior paediatrician (the CIN clinical coordinator) also followed up the provision of reports with specific emails and phone calls (after 1–2 weeks) to the paediatricians highlighting specific areas of success and areas requiring continued improvement. These calls aimed to provide encouragement and support as part of building a sense of being within the network." (Gachau et al 2017)*  *"CIN leaders attempted to link recognition of senior colleagues and peers to aﬃrming professional values, challenging a traditional, authoritarian medical culture in Kenya, which was seen to perpetuate outdated clinical practice. CIN Director 25 noted that CIN “beneﬁtted from trying to present… a new way of doing business”, which junior health professionals were “receptive to” due to a “dissatisfaction with the sort of old professor stands in the corner and tells you … [CIN] ﬁtted into that generational issue, of the expanded internet … seeing that there is more than just doing what you were told ﬁfteen years ago.” This new approach appealed to younger health professionals." (McGivern et al 2017)* |
| 5K. When networks have processes in place to identify and enable those with the necessary skills, motivations, or attitudes to take on leadership roles (context) this may support network functioning and may lead to changes happening (outcome), because it can draw on wider pool of engaged resources (mechanism) | *"The initiative relied heavily on mobilizing the District Clinical Specialist Teams and program managers to steer and implement collectively agreed-upon strategies for improving MNCH services. Also, key were nursing managers in the districts, hospital and PHC services and the dietitians who formed a core of stable professional cadres, in the face of a high turnover of doctors and political appointees at more senior management levels. The MRU offered new spaces of decision-making, participation and recognition for these players. Dietitians, for example, emerged as the key leaders of the response to malnutrition, authorized through the MRU to engage and lead the team response. Similarly, as related by a Ward Based Outreach Team (WBOT) coordinator: “…through this, now suddenly they recognize WBOTs. They see what they are really used, [for] what they can really be used for."*  *At a formal level, the MRU exemplified a hierarchical mode of governance: Starting at the district level, the driver is the district manager, and then the rest of us follow. At the subdistrict level at the hospital the CEO is the driver, and all other units will follow. And then from PHC level, the PHC manager of that particular sub-district is their driver, and then the navigators will follow. (District Dietitian) However, at an informal level, the “real” drivers were often the middle managers at district and sub-district levels (Figure 2), functioning in a more networked mode of governance." (Schneider et al 2020)*  *“Rotating chairs between clinical disciplines was regarded as a strength by those in the network groups and as an effective strategy for building local support, because it ‘gets away from the doctor-boss type thing’ and signals that the network values the contribution of all disciplines. The need for appropriate representation in working groups and on governing committees was also seen as important for the same reason: “If people saw it as being just the teaching hospitals driving this then our district hospital and our GP services wouldn’t want to be a part of it. So you’ve got to have an appropriate level of representation.”” (McInnes et al 2012)*  *"The CIN focal persons in each hospital are ‘mid-level’ managers who lead departments and teams. In these clinical hybrid roles, they are responsible for improving documentation in medical records that facilitates data collection and ongoing improvement work. The focal persons bring local authority and a distributed form of leadership that promotes CIN legitimacy and credibility. They also understand the local context of hospital systems and power relationships, and play key boundary spanning roles in changing the behaviours of frontline workers that are critical to achieving improvements in care and outcomes. Engaging such personnel in improvement work does demand, however, that they have the capability, that the environment offers them the opportunity and that they remain motivated in what can be challenging low-resource settings." (Irimu et al 2018)*  *"However, even where the CEOs and medical managers were not active players, the MRU was able to meet regularly and make a positive contribution if a stable core of middle managers in both hospital and PHC services saw its value. In the presence of formal authorization of the MRU from above, locally specific, informal coalitions, led in large part by senior nursing managers, were able to anchor and sustain implementation of the MRU." (Schneider et al 2020)*  *"In District 1, there had been three acting District Directors since the inception of the MRU, and at the time of the interviews, the district management team had not met formally for 6 months. Although the MRU was written into job descriptions of hospital CEOs and PHC managers, there was limited active oversight of sub-district MRUs by district managers. However, active support from one senior member within the district management team, and from the DCST and program managers, ensured a sufficient degree of cooperation for the district MRU to meet regularly and be considered effective....In sum, there was more evidence of cooperation with the MRU as a strategy in District 2 than in District 1, even if in the latter the MRU had enthusiastic proponents who ensured its ongoing functioning." (Schneider et al 2020)* |
| 5L. If a network is highly dependent on a few people to implement network activities (context), then it is at risk of being unsustainable (outcome), because of burnout and/or loss in those tasked with implementation (mechanism) | *"despite seven years of QRP implementation and agreements, the management of the QRP is not yet formalized in the job description of the head of the Ob/Gyn Department. This oversight has the potential to create a vacuum once dedicated personalities step away from daily work for QRP. Recognizing this risk, a roster was formed of Ob/Gyn residents who are tasked with management of QRP on a rotating basis." (Vergara et al 2020)*  *“It was remarked that beyond the establishment phase, with a good network manager in place, the network should become less dependent on the network chair. Some participants from both groups interviewed, believed that those networks that were highly dependent on the activities of the network chair were less likely to have impacts because the network became a ‘one-person show’, with ‘limited reach’ as well as burning-out the network chair.” (McInnes et al 2015)* |
| 5M. When network leadership are not able to get the network to develop and agree on a shared vision (context), then it is at risk of not performing well and becoming unsustainable (outcome) because members are unclear as to what the network is for (mechanism) | *“It was remarked that beyond the establishment phase, with a good network manager in place, the network should become less dependent on the network chair. Some participants from both groups interviewed, believed that those networks that were highly dependent on the activities of the network chair were less likely to have impacts because the network became a ‘one-person show’, with ‘limited reach’ as well as burning-out the network chair.” (McInnes et al 2015)* |

### Developing a network identity and culture

| 6A. When network members with a collective identity come together to solve a shared problem (context) this makes them feel fulfilled (outcome) because of a sense of shared purpose (mechanism) | *“The PAR process improved interactional factors between managers of the network “(…) Hospital: “There’s nothing more I can do because primary care is doing such a terrible job”. Primary care centre: “There’s nothing more I can do because the hospital is doing such a bad job” (…) And with that we don’t get anywhere constructive (…) I would say that this in some way....means we basically see ourselves as one and the same. So we’ve really managed to lower our defensive barriers (…) and start to understand that what we really need to do is help each other out” (LSC, Chile). “it’s like a sign that a shared vision of the network is appearing, which was the problem we had in the first place (…) Of course, and people perceive it and everyone can feel it now. If I’ve got a problem I know who to speak to and I know they’re going to help, you know? And the certainty that they’ll help me, that they’ll collaborate with me for the sake of the patient”” (LSC, Chile) (Vargas et al 2020)*  *"Bi-annual network meetings helped to develop paediatrics as a professional community in Kenya. CIN Director 25 described one of the network's aims as: “Truly being able to engage with one's peers … for the profession to begin to take more of a view … a stand on what is happening and begin to own that agenda.” CIN meetings enabled participants to share experiences, learn from colleagues facing similar challenges and develop as part of a multi-professional and multi-organisational paediatric community. Nurse-in-charge 30 noted: “CIN has been great. It has made me meet so many other people from diﬀerent organizations and different work backgrounds but we are all pushing towards the same goal.” Similarly, Paediatrician 10 described how by “coming together” and being able to “share challenges and successes and learn from others” CIN members “move together to improve the quality of care for our children [patients], individually and then collectively.” (McGivern et al 2017)* |
| --- | --- |
| 6B. When network members feel fulfilled from working with likeminded people in a network (context) they want to belong to the network (outcome) because they feel it is worthwhile (mechanism) | *“The implementation history of the PCNs suggests an evolving and nuanced role for cultural adaptation as a strategy for achieving longevity, popularity, and transformation towards a physician-focused interpretation of PHC. Our findings suggest a super structure of cultural transformation has emerged from and been made possible by a base layer of requisite, legally as identified elsewhere, acknowledging and adapting interventions to these closely held cultural values is clearly a key element in making PHC transformation not just possible [68] but seen as legitimate by family physicians [73]. In Alberta’s case that legitimacy, generated out of cultural concordance and a sense of local ownership of self-made solutions has held [61, 74, 75]. Indeed, it has thrived, with over 80% of the province’s physicians making the decision to sign contracts and remain members of their PCNs even as the Era of Accountability with its opposing cultural values arrived. The PCNs have survived and grown in popularity in part because they offer a cultural redoubt where independence and local action are protected and respected.” (Leslie et al 2020)*  *"Face-to-face meetings remain important to create and sustain individuals’ and institution’s identification with the Clinical Information Network (CIN) and consequently help overcome turnover of specific individuals." (Irimu et al 2018)* |
| 6C. When network members identify with other network members and its vision (context) they develop a network identity (outcome) because it gives them a sense of purpose (mechanism) | *"To help develop ‘engaged and motivated’ members of CIN at local levels and build a collective identity and community of practice, we hold twice yearly face-to-face meetings with paediatricians inviting the other CIN focal persons to one annual meeting.9 These meetings allow participants to discuss their audit reports (including offering suggestions for improving data collection) and to identify underlying problems and potential solutions with peers. They also provide a forum for short, specific skills building sessions focused on the ‘soft skills’ needed as a manager (eg, how to run a team) and on building their understanding of research. Crosstalk among the scientific, clinical and policy communities in an arena promoting collegial relationships helps interpret results based on an understanding of practice in the real world. This enables all sides to understand how contexts interact with improvement approaches to bring about observed outcomes26" (Irimu et al 2018)* |
| 6D. When a network creates opportunities for members to connect, share experiences, and learn from each other in an open and safe environment, (context) this creates a positive network culture and a feeling of belonging to the network (outcome) because they feel respected and valued (mechanism) | *"Bi-annual network meetings helped to develop paediatrics as a professional community in Kenya. CIN Director 25 described one of the network's aims as: “Truly being able to engage with one's peers … for the profession to begin to take more of a view … a stand on what is happening and begin to own that agenda.” CIN meetings enabled participants to share experiences, learn from colleagues facing similar challenges and develop as part of a multi-professional and multi-organisational paediatric community. Nurse-in-charge 30 noted: “CIN has been great. It has made me meet so many other people from diﬀerent organizations and different work backgrounds but we are all pushing towards the same goal.” Similarly, Paediatrician 10 described how by “coming together” and being able to “share challenges and successes and learn from others” CIN members “move together to improve the quality of care for our children [patients], individually and then collectively.” (McGivern et al 2017)*  “*Knowing each other in person, according to informants, helped to improve their opinions and understanding of the other care level, increased their willingness to collaborate and find joint care strategies, facilitated direct communication for patient follow-up, and in Chile, moreover, bolstered their feeling of belonging to the network: “… barriers are now being broken down between the specialist [and the general doctor], ‘because I’m the one who knows the most and the general doctor has no idea’. So now the specialist is starting to see the reality of a general doctor’s work with all its limitations (…) That’s when they open up their world view and say, ah right!, well my suggestion would be this, or whatever other path that allows the patient to improve their condition and their quality of life” (Healthcare Manager, Colombia); “I think there’s certainly been a great contribution to coordination. For a start, this situation of acknowledgment…, this situation of acknowledgement of the different parts (...) And I think that this respect that has kind of been generated between one and the other paves the way, it creates the chance to build something together” (Healthcare Manager, Chile). In Chile, this improvement in interactional factors – personal relationships, trust – also occurred among administrators and middle managers that participated in creating the cross-level instruments to implement the interventions (LSC, PP, crosslevel working groups), and spread to other instances of cross-level management already present in the network.” (Vargas et al 2020)*  *"In Kenya, health professionals often work in remote district hospitals, overseeing clinical departments with little support or training. Many find this difficult, particularly without resources to provide high standards of care. Consequently, loss of motivation and burnout are common (Brown, 2016; Mbindyo et al., 2009). CIN leaders attempted to create a supportive network community to addressed this problem, by providing physical and online spaces in which to meet and share as a professional community. CIN encourages network participants to think about new approaches to problem-solving, and then implement, test and measure solutions, and facilitates sharing of learning across participating organizations, thus functioning as an improvement collaborative (English, 2013)." (McGivern et al 2017)*  *"To help develop ‘engaged and motivated’ members of CIN at local levels and build a collective identity and community of practice, we hold twice yearly face-to-face meetings with paediatricians inviting the other CIN focal persons to one annual meeting.9 These meetings allow participants to discuss their audit reports (including offering suggestions for improving data collection) and to identify underlying problems and potential solutions with peers. They also provide a forum for short, specific skills building sessions focused on the ‘soft skills’ needed as a manager (eg, how to run a team) and on building their understanding of research. Crosstalk among the scientific, clinical and policy communities in an arena promoting collegial relationships helps interpret results based on an understanding of practice in the real world. This enables all sides to understand how contexts interact with improvement approaches to bring about observed outcomes26" (Irimu et al 2018)*  *“The CIN has been relatively successful in creating an opportunity for frontline caregivers, health researchers and informatics specialists to learn as a community to improve availability of clinical data and begin to promote their use. The hospitals in the network have begun supplying and promoting the use of more structured medical records. This has been helped, we believe, by slowly changing the hospital culture through sustained engagement and by providing peer support by linking hospitals within the network.937 In this way, new staff quickly become familiar with the clinical forms and are integrated into thinking about data-informed quality improvement efforts at the hospital level, something that is not routine.37 38” (Tuti et al 2016)*  *“At micro- level: absence of an enabling environment at facility level created a culture that gave little incentive for health facility staff to learn QI skills. It was further compounded by a rigid hierarchical system and partial administrative support especially encountered in the urban hub facility. Maintaining motivation in physicians was a challenge. Both the rural and urban facilities lacked dedicated hospital QI personnel, especially at the urban- hub facility; this made healthcare providers visualise QI as extra work, which distracted them from their regular patient care. Non- provision of transportation for hub mentors to their allocated spoke facilities added to poor motivation to undertake mentoring visits.” (Srivastava et al 2020)* |
| 6E. When network leadership and members identify that the network’s culture is not aligned to existing cultures (context) then they may attempt to change the pre-existing cultures and instil a culture (outcome) because they do not identify with it (mechanism) | *"No restructuring of the physical environment in participating hospitals was conducted by the network team, but we did change the medical record systems and furnish information where it did not exist. This information on adherence to guidelines is aimed at creating a professional and social expectation to improve. Linked to a sharing of this information across groups and peers we were also aiming to create new norms and to revise socially constructed professional roles." (English et al 2017)*  *"Between network inception and November 2016 hospitals have received 15 feedback reports. We observed that hospital teams are keen ‘not to be in the red’ but ‘be green’ in their report. Comparison of hospitals’ performance especially during face-to-face meetings helped foster shared learning rather than potentially harmful competition and helped people gain confidence in their ability to achieve change. We attribute this to having a shared vision and referring to poor performance as ‘offering opportunity for learning and improvement’ while engagement with peers provided encouragement to complete quality improvement processes.25 As a result, clinical teams worked with their records department and hospital administration, drawing lessons from across CIN, to ensure hospitals acquired infrastructure, reorganised service delivery and leadership provided effective mentorship and supervision to support delivery of quality care" (Irimu et al 2018)*  *“In addition to creating lines of communication and accountability, the hope is that, through their work, the committees will mitigate the mutual mistrust of the two joint venture partners by bridging their cultural differences. These cultural differences are not just a theoretical challenge, but also a source of significant tension in the applied work of co-planning. As one participant noted, the PCNs find themselves caught up in their members’ cultural expectations and operational norms: Primary care doctors are used to getting the information [from a patient] and making decisions and getting out of the [exam] room in 12 minutes. And they bring that same attitude towards their PCNs and their PCN operations. Now obviously, PCNs can’t make changes that fast. And so, [the doctors] already find PCNs slow. Then, [in the case of] PCNs working with AHS, AHS seems to move, from a primary care doctor’s [perspective at] a glacial speed.” (Leslie et al 2020)*  *"These various changes were associated with a new culture of engagement with clinical guidelines and the intensification of in-service “drills,” providing the software mechanisms which brought the pre-existing hardware strategies to life: It has also sensitized people about using the guidelines. People had guidelines but they were kept in the drawers, they were kept in the offices, locked up there … it has cultivated a culture of reading, reading the guidelines and discussing the guidelines. (DCST member)" (Schneider et al 2020)*  *"A professional culture of compassionate service and pragmatism pervades the ranks of both public and private providers. Before formation of the Network of Care, this culture was not perceived as shared; each sector reportedly mistrusted the practices and intentions of the other." (Vergara et al 2020)* |

### Commitment

| 7A. When networks members identify with the network’s collective vision, identity, and culture (context), they are more likely to be committed to the network (outcome), because they believe in and value these (mechanism) | *“Policies developed by the PCIC were adapted and tailored to meet the needs, resources, and abilities of the individual FHTs. FHTs’ executive directors and lead physicians on the PCIC were supported and encouraged to use a ‘bottom-up’ approach to solve problems in a context- dependent manner, which fostered continued buy-in. Patients and family members who described their experience viewed the RTs as members of FHTs and did not see a division in the provided healthcare services. The shared development of the network led to shared success of the entire health team. Their co-creation of vision and goals led to shared commitment and ultimately shared success.” (Sibbald et al 2020)*  *“Professionals’ interest, enthusiasm and commitment, which increased as they participated in the joint meetings (Supplementary Box S2), emerged strongly in Chile and Colombia, in particular, and also in Mexico with regard to the joint training sessions in maternal and perinatal care. The aspects that mainly contributed to this interest were the opportunity to ‘receive training’ and ‘improve quality of care through coordination’. The latter reason was also cited in Brazil, and ‘CV improvement’ was given as a further motive in Mexico and Chile. This interest led professionals in Colombia and Chile to change shifts, attend out of working hours, administrate meeting spaces and help to spread word of the intervention: .... for example, like in Unit XXX the doctors themselves took control, managed their own spaces, fought over their place to be able participate .... They didn’t give them any time..., but they would walk in there with the clinical case and documents reviewed (LSC, Colombia)” (Vargas et al 2 2020)* |
| --- | --- |
| 7B. When networks members have support from leadership/administration  /stakeholders (context), they are more likely to be committed to the network (outcome), because they feel valued (mechanism) | *“The ‘institutional support’ of network managers emerged in all five countries as the main organizational factor that influenced implementation of the interventions (Supplementary Box S2). In Chile, and in Brazil with the occasional interruption, it was identified as a facilitator that remained stable throughout the process. In Mexico, it was strengthened following political change; in Colombia, it was weakened; and in Uruguay, it remained frail. It was considered key in terms of whether the resources needed (professionals’ time and materials) were available to implement the interventions (and in Brazil and Mexico also to solve operational problems—see Supplementary Box S2): ‘...there was support from the managers to protect these (allocated project) schedules, that normally doesn’t happen with this kind of more low-scale project’ (Professionals’ inter-level working group, Chile); As for the managers, there was no interest. Not little interest, no interest! There should have been more coordination on our part, more involvement, because it depended exclusively on us (Healthcare manager, Colombia).” (Vargas et al 2 2020)*  *“It was felt that local health service manager representation on network committees would ensure that networks deliver projects of relevance to the region covered by the health service and help to increase health service and local clinician buy-in to networks.” (McInnes et al 2012)*  *“change in authorities to ones with new priorities led to institutional support for the intervention, in conjunction with the participatory nature of its design, and the use of a reflexive method was implemented. On the other hand, regarding the joint training meetings on chronic diseases, a low impact was observed due to its limited implementation, which was related to the lower institutional support; thus, neither coordination nor its factors were affected” (Lopez-Vazquez et al 2021)* |
| 7C. If a network has committed members (context) then they are more likely to act on their identified problem and collective vision (outcome) because members are willing to put in the energy and effort (mechanism) | *"In the case of upwards referral of complicated cases and emergencies, communication capacity was improved. Every node in the network and each ambulance was equipped with smartphones connected by a closed user group (CUG). As the original phones have aged out of use, providers shift to using their own handsets and utilize credits issued monthly by their facilities for that purpose. WhatsApp is currently the main channel of communication; there are at least five groups in the CCBRT NOC dedicated to different parts of NOC activity, including blood transfusion availability, maternal and perinatal death review, and ambulances." (D’mello et al 2020)*  *"Communication around referrals is standardized, where the clinical details of both inward and outward referrals are communicated by telephone before the patient arrives to prepare the receiving providers as well as to confirm that there is space available. Details are also documented on three-ply paper referral forms. QRP has a dedicated phone line, which the on-call Ob/ Gyn at QMMC carries, to contact when there is an obstetric emergency. Routine communication is also standardized; a group SMS is used for messages about QRP meetings and other updates, such as infrastructure work at facilities that may cause delays or reduce the number of available beds." (Vergara et al 2020)*  *"Moreover, unlike in the wider Kenyan health system, there appeared to be a sense of ownership of data collection within CIN because: “It's very clear, [CIN] it's actually trying to use data to improve the quality of care … we own it … the fact that I don't have to send data to KEMRI does not stop me from collecting my own data” (Paediatrician 4). HRIO 29 commented: “I'm very passionate about CIN because, as our vision says … we are using this data, which we are generating to improve the quality of services to patients.” Nurses were also described as “owning” CIN data and using it to “assess the nursing work … holding each other accountable” "(Paediatrician 11). (McGivern et al 2017)* |
| 7D. When influential outside stakeholders are actively engaged in network processes (context) then they are more likely to be committed to the network (outcome) because they can be helped to understand the collective vision of the network (mechanism) | *“One of the key aspects to OHW’s approach is collaborative co-investment from the municipalities. For any intervention that involves cost-sharing, birthing center renovations, for example, an MoU will be established between OHW and the municipality. The municipalities have increased their co-investments in birthing center renovations over time, from 21% in 2018 to a planned 47% in 2020.” (Bhatta et al 2020)*  *"EMAS approaches were designed to help districts and facilities operationalize existing Government of Indonesia policies and programs, and EMAS tools and approaches were included in local government policies; therefore, many EMAS approaches could be funded using local budgets. Tools and approaches were crafted to enable facilities to more readily meet established government standards and expectations regarding the provision of emergency maternal and newborn care within facilities and throughout the referral process." (Hyre et al 2019)*  *"Getting hospital management to buy-in to the NOC structure was difficult at first because they feared losing income through outward referrals to lying-in clinics and had doubts about the quality of care possible at those sites. Persistent advocacy by QRP leadership eventually assured hospital administration buy-in by contending that rational distribution of cases would improve quality of services delivered and not result in income losses, demonstrating early signs of this success through presentation of QRP data and less formal anecdotal evidence." (Vergara et al 2020)*  *"In order to engage local parish and municipal government representatives to promote awareness of and adherence to national laws mandating coordinated service provision through a public health-care system, the project organized several meetings where such laws and national priorities were discussed" (Broughton et al 2016)*  *“Upon arrival, the maternal health workers conducted a series of meetings with local authorities, village heads, religious leaders, traditional healers, women’s and other civil society groups, and local health workers and TBAs, to explain the programme. The meetings followed an informal, participatory approach, allowing stakeholders to offer opinions and make recommendations. This process of informing and sensitising the community was envisioned as a necessary and appropriate step to secure support for the project.” (Mullany et al 2008)*  *"A history of successful prior engagement with multiple stakeholders that fosters trust between parties is an important foundation supporting local ownership and leadership while it is important that the network addresses each party’s interests." (Irimu et al 2018)*  *“In the current study, we found that members of the community and staff routinely reported a sense of ownership and active involvement in ensuring its success. Participants also reported that this local participation and ownership in priority setting was pivotal for the community buy-in and continued success at 18 months of the RTT initiative.” (Nahimana et al 2016)*  *“It was seen that the district and state level administrative buy- in/ownership was deficient. This was possibly due to lack of insight on benefits of implementation of QI at scale.18 This became one of the major bottlenecks in the effective implementation of the HSM. The major learning from this implementation exercise was that in absence of effective handholding and support at macrolevel long- term sustenance of any micro- level and mesolevel interlinked process is a formidable challenge.” (Srivastava et al 2020)*  *“Lack of sensitisation about QI methodology among the district and NHM officials led to deficient buy- in/ownership and consequent support. Lack of active participation from the district officials and state NHM officials on account of other competing priorities for district and state health officials.” (Srivastava et al 2020)*  *“Macro- level: lack of ownership and financial provisioning by the state administration for scaling up of this initiative across districts led to non- sustenance of the operational model. Irregular monitoring and evaluation of the QI work progress by the state and district officials led to lack of accountability for the process. The transfer of a senior official from health department led to significant delays in operationalising of the project.” (Srivastava et al 2020)*  *“When we spoke with the authorities, some situations that could hinder the system (the offline virtual consultations) were perceived. One of them was the lack of computers and internet at the Health Centres, for which a census was carried out… and after the census, the Ministry of Health committed to correct this situation… when a government institution is committed, there is 20% of certainty they will comply, while 80% they won’t comply. That happened here or at least primary care physicians still complain about this and they still report that they do not have a convenient internet access and they sometimes do not even have computers.” (Local Steering Committee/Manager). (Lopez-Vazquez et al 2021)* |
| 7E. When influential outside stakeholders are committed to the network and actively engaged in participating (context) then it may be easier for the network to achieve its aims (outcome) because network members feel empowered (mechanism) | *“In an effort to fill human resources gaps, which are particularly acute in rural and remote areas, municipalities directly contract health workers. A health facility will make a request for staff to the municipality, which advertises the post locally and selects applicants. Contracted providers’ salaries are paid directly by the municipality from their local budgets. Given this local-level capacity to fill workforce gaps, OHW does not typically hire additional health workers to serve in the NOC.” (Bhatta et al 2020)*  *“There have been additional investments in infrastructure, human resources, drugs and supplies, and materials for women at the birthing center. The municipality built a maternity waiting home with the health council’s budget and purchased three ambulances that, while they do not meet international standards, serve as dedicated transport for free maternal, neonatal, and child health care. They have recruited a laboratory assistant and, as they are not able to regularly rely on government stock, set aside a budget for medicines and created buffer stocks to support facilities. Additionally, the municipality provides blankets to mothers at the birthing centers and is now considering providing warm clothing for newborns. They have also provided FCHVs with cabinets to hold their job aids and training materials." (Bhatta et al 2020)*  *"The CBMIS built on an existing but largely dormant system that relied on traditional leaders and CBAs to locate and document live births, stillbirths, maternal, and neonatal deaths. It also provided useful information for decision and policymakers to better understand health challenges for mothers and newborns at the community level. The responsibility of community data collection and analysis was transitioned to the LGA M&E officers and the state HMIS officers to ensure full government ownership and continuation of the CBMIS." (Fasawe et al 2020)*  *"To source funding for recurring costs such as fuel, CHAI worked with district and village heads and Ward Development Committees to establish sustainable contributory schemes. By June 2016, 65% of communities had taken full responsibility for maintenance and operation of the MBAs." (Fasawe et al 2020)* |

### Engaged and motivated network members

| 8A. When network leadership provides opportunities for network members to be supported, recognised, and learn (context) this creates engaged and motivated network members (outcome) because they derive direct benefits (mechanism) | *"To help develop ‘engaged and motivated’ members of CIN at local levels and build a collective identity and community of practice, we hold twice yearly face-to-face meetings with paediatricians inviting the other CIN focal persons to one annual meeting.9 These meetings allow participants to discuss their audit reports (including offering suggestions for improving data collection) and to identify underlying problems and potential solutions with peers. They also provide a forum for short, specific skills building sessions focused on the ‘soft skills’ needed as a manager (eg, how to run a team) and on building their understanding of research. Crosstalk among the scientific, clinical and policy communities in an arena promoting collegial relationships helps interpret results based on an understanding of practice in the real world. This enables all sides to understand how contexts interact with improvement approaches to bring about observed outcomes26" (Irimu et al 2018)*  *"LKBK provided mentee facilities with a clear picture of good clinical governance and quality services, helping to generate a sense of urgency to change within those facilities across six provinces. Using mentoring on a large scale increased motivation and performance of health facility and health office staff and broke down barriers between public and private facilities" (Hyre et al 2017)*  *“Initiatives that addressed workforce development and clinical education were cited as important outcomes as they were thought to lead to other desirable outcomes such as job satisfaction, the development of clinical career paths and the retention of a stable clinical workforce that could carry forward network initiatives. Obtaining funding for clinical positions; scholarships and speciality post-graduate studies; clinical fellowship posts; and conference support schemes and the establishment of new postgraduate courses in clinical specialties were all cited as desirable network outcomes because they contributed to retention of the workforce and enabled professional pathways into obtaining specialist qualifications: “We have been able to fund so many nurses to do post-graduate studies in neurology. So it’s made stroke more of a career. In my opinion – it’s been a major retention for neurosurgery.”” (McInnes et al 2012)*  *“In the experiences with joint meetings, with the exception of Brazil, informants agreed that the ‘type of intervention based on personal contact’ and the ‘continuous adaptation of content to professionals’ needs’ fostered their motivation to participate and encouraged them to get to know and trust doctors of the other care level better.” (Vargas et al 2 2020)*  *“The challenges with the branding of CLAHRC resulted in the need to actively sell the benefits and opportunities of getting involved. Those working in boundary spanning roles were particularly important in prompting connections through their interactions and activities with both academics and practitioners: And so establishing and doing these sort of teaching sessions at first, we did quite a lot of study days where staff came from the Trust. And [name of facilitator] was the biggest help in getting over any sort of barriers and boundaries because she was there, she knew the Trust, she could sort of go and…work with the staff at a ward level…and constantly reinforce the message. (Boundary spanner, Oakdown)” (Rycroft-Malone et al 2017)*  *"Further, they described how their team’s motivation was inspired largely by improved hospital management. When the medical officer in-charge and the hospital Matron walked around facilities to check-in with health workers, inquiring about maternity care and identifying how they could assist, it fostered a positive environment with improved teamwork. The clarification of a sense of purpose and confidence in leadership promoted by the NOC formation nurtured these and other better management practices." (D’mello et al 2020)*  *"The Dar es Salaam Regional Health Authorities, in collaboration with CCBRT, organized annual quality assessments to spur interfacility competition; rewards of recognition and prizes, such as baby warmers, vacuum extractors, and portable ultrasounds, are given at regional meetings. Later, management from one hospital independently organized cash rewards from their own budget for high-performing providers, as well as night shift allowances for labor ward staff. While financial incentives for increased motivation and teamwork reportedly made a difference in that setting, the NOC also used other modes of recognition of good performance without financial incentives; these also had positive effects noted through better performance, suggesting alternate ways to signal a caring professional culture can trigger and support better performance." (D’mello et al 2020)*  *"Use of facility data was enhanced by introducing a standardized set of service statistics to be collected by each facility. Data were aggregated monthly and analyzed to track coverage of evidence- based, high- impact clinical interventions. Facility staff charted monthly performance on laminated “data for decision making” posters and reviewed the data during regular internal meetings. Facilities reported the data to district health authorities using existing health information system processes and forms. Facility staff also presented selected data to the district- level MNH working group for joint problem solving. The most important clinical and operational indicators, defined by each ward/unit, were presented visually in wall- mounted data visualization dashboards to motivate teams to take action to address suboptimal performance." (Hyre et al 2019)*  *“The principal motivation for surgeons’ continued participation in pooled effort events is the opportunity to learn from each other (regardless of seniority) about fistula surgery and engage in ongoing training, coaching, and mentoring with colleagues. Besides developing their own skills, they are also able to help each other by reducing backlogs (as well as forming a local network of surgical teams). Pooled effort events also provide visiting surgeons with an opportunity to focus on fistula repairs without having to spend time on other clinical and administrative duties.” (Fistula Care 2010)*  *“Second, the working environment generated by the restructuring of the network, involving redundancies and salary cuts, reduced doctors’ interest in participating: .... we got to do the meetings, and then before that they’d told off the doctors, they’d audited their accounts, they told them they were going to cut their salaries, etc. That creates bad feeling between people (...) that hindered to a certain extent the rollout of the sessions (PP, Colombia)” (Vargas et al 2 2020)*  *“At micro- level: absence of an enabling environment at facility level created a culture that gave little incentive for health facility staff to learn QI skills. It was further compounded by a rigid hierarchical system and partial administrative support especially encountered in the urban hub facility. Maintaining motivation in physicians was a challenge. Both the rural and urban facilities lacked dedicated hospital QI personnel, especially at the urban- hub facility; this made healthcare providers visualise QI as extra work, which distracted them from their regular patient care. Non- provision of transportation for hub mentors to their allocated spoke facilities added to poor motivation to undertake mentoring visits.” (Srivastava et al 2020)*  *“In contexts where there was an absence of a history of established relationships and collaboration, where there has been less activity around the joint setting of priorities either at bid development stage, the need to sell the benefits of CLAHRC to encourage or incentivise engagement of mutual benefit was evident: I think that getting people engaged in it is about them seeing there’s some mutual benefit and where there wasn’t seen as any mutual benefit it doesn’t happen…I know other CLAHRC Directors feel a bit the same, that you feel like you’re a salesperson going round trying to sell things. (Leader, Hazeldean)” (Rycroft-Malone et al 2017)* |
| --- | --- |
| 8B. When a network member gets ‘emotional’ benefits (positive feelings) or feel a sense of purpose from being part of the network (context), they are likely to be highly committed (outcome), because it is fulfilling for them (mechanism) | *“However, a lack of interest in participating also emerged in all five countries, with greater intensity in Uruguay and Brazil in general; and with regard to offline virtual consultations in Mexico, and replica meetings (meetings with other network doctors not in the PP) in Colombia. Contributing factors, according to interviewees, were: the ‘limited adherence to the primary care-based model’ of some SC doctors in Brazil, Chile and Uruguay and ‘not knowing the doctors of the other care level’, ‘mutual mistrust’ and PC doctors’ ‘shyness and fear to express their doubts’ in Chile, Colombia and Mexico. However, these obstacles diminished as they participated: Yes, yes, at the start I found it really difficult to participate (.. .) at first it was like: ‘um...what am I going to do there? They’re all going to be looking at me, I’ve got to give a reply, it’s scary, I don’t want to do this’ (.. .). But it’s all got a lot better, (...) they want to participate, because they’re enjoying it. (Administrative personnel, Chile)” (Vargas et al 2 2020)* |
| 8C. When network members’ personal identity strongly aligns with a network’s identity and culture (context) then this will result in engaged and motivated network members (outcome) because they feel they belong (mechanism) | *“Our findings demonstrate that when beliefs, values and priorities among members of the referral network converge, this fosters cooperative behaviours which are beneficial for individuals and the network as a whole. When values, beliefs and priorities diverge, self-interest prevails to the benefit of individual clinicians but impacting negatively on other clinicians and potentially on patients.” (Pittalis et al 2021)* |
| 8D. When network members actively participate in network change practices that align with the professional values they live by (context) this creates engaged and motivated network members (outcome) because it helps them to fulfil their moral obligation or vocational calling (mechanism) | *"The effect on motivation of the intervention described is mainly perhaps through the reflective pathway with participants engaged in developing plans and evaluating progress against goals that are shared with experts and peers (in common with more specific quality improvement strategies [26]). However, we believe automatic motivation may be triggered by a link to an innate desire to provide good care encompassed in the idea of vocation amongst health workers [27]." (English et al 2017)*  *"Many doctors and nurses we interviewed were motivated by providing good patient care “The most satisfying thing about my job is when I see my children [patients] going home healthy” (Paediatrician, 10) and/or developing younger colleagues (“the satisfying thing has been teaching younger colleagues; to see the transformation from a doctor who had just learnt the theoretical knowledge to actually being able to apply it at the bedside” (Paediatrician 11)" (McGivern et al 2017)*  *“The principle of multidisciplinary collaboration was also believed to foster goodwill amongst clinicians and had fostered an ethos which was ‘collaborative rather than antagonistic’. This was thought to have resulted in two positive effects: i) reinvigoration of clinician interest in participating in projects to improve quality of care and facilitate health system changes, and ii) provision of a forum for developing projects that addressed patient health needs in collaboration with the state-based Ministry of Health and other external partners. This is summarised in a quote from a network chair: Networks make it easier for change and broad planning, it is the composition, structure and organisation of networks that has enabled achievements to take place.” (McInnes et al 2015)*  *“Most participants stated that successful networks were strengthened by formal organisational structures and processes and having ‘solid systems’ in place that facilitated effective planning and communication. These systems included project workgroups focused on project planning and implementation, with broad clinical and consumer representation and meetings with structured agendas and minutes: ‘Not chat-fests that result in nothing’. Those networks where the main objective was ‘just about showing up at meetings and giving a progress report’ were perceived by many as those that made little impact. Open governance and leadership rotations were regarded as important for securing and maintaining engagement and for ongoing reinvigoration of the network: Makes people feel engaged, willing to give solid commitment and feel part of the network’s mission” (McInnes et al 2015)*  *“Across the spectrum of stakeholder groups, the overall impetus for participation in network activities and establishment of networks was to work for the patient cohort and to improve the effectiveness of clinical services. This could be done through network projects that add value to existing services by becoming involved in service planning and improving the delivery of services. This was expressed in comments such as: ’removing the waste in the system’; and ‘breaking down the barriers to the provision of care for patients in NSW’.” (McInnes et al 2012)*  *"Coordinated action was driven by shared goals, recognition of inter-dependence, and greater shared responsibility: We all share the same goal and it’s kind of motivating. (Dietitian) It is like a link, everyone is linking with the other so everyone is playing his or her role, that’s what I can say … We are working like this because the one can’t survive without the other. (Hospital manager)" (Schneider et al 2020)*  *"To help develop ‘engaged and motivated’ members of CIN at local levels and build a collective identity and community of practice, we hold twice yearly face-to-face meetings with paediatricians inviting the other CIN focal persons to one annual meeting.9 These meetings allow participants to discuss their audit reports (including offering suggestions for improving data collection) and to identify underlying problems and potential solutions with peers. They also provide a forum for short, specific skills building sessions focused on the ‘soft skills’ needed as a manager (eg, how to run a team) and on building their understanding of research. Crosstalk among the scientific, clinical and policy communities in an arena promoting collegial relationships helps interpret results based on an understanding of practice in the real world. This enables all sides to understand how contexts interact with improvement approaches to bring about observed outcomes26" (Irimu et al 2018)*  *"Yet good intentions were frustrated by the Kenyan health system. Paediatrician 3 noted: “Frustration is actually top on the list… I wish I had this [equipment/drug], I would be able to save this baby … your potential is being utilized 20–30%, you get bored … your hands are tied.” This undermined motivation to improve care. Paediatrician 21 noted: “Motivation … is gone. You go to work at eight and you leave at four. It's just work now, it's not how can I make [health care] better."" (McGivern et al 2017)*  *"There was, however, only a small improvement in recording blood glucose levels overall in seriously ill children although two hospitals achieved the >60% target (figure 2C). Informal discussions with CIN focal teams suggested this was in many cases linked to difficulties in securing adequate supplies for bedside or laboratory-based glucose testing. Lack of resources then undermined clinicians’ motivation to request the test." (Irimu et al 2018)*  *“Some in the network groups mentioned that not having ‘power or teeth to implement changes’ placed networks at risk of losing the enthusiasm of involved clinicians. The need for networks to be empowered to make changes was also acknowledged by some in the Senior policy-maker group: ‘need to sort out who is responsible for implementation. Feedback from the Senior manager group highlighted that to facilitate implementation, networks needed to garner the support of local health services to implement and ensure that projects and innovations are clinically relevant.” (McInnes et al 2012)* |
| 8E. When a network can show its members that it can affect some change (context), members are more likely to continue their engagement to it (outcome) because of they can see its value (mechanism) | *"Other more subtle techniques were employed to create a culture of change at QMMC in particular. For example, as the doctors were being introduced to the idea of the QRP, the Ob/Gyn ward was repainted with the intention of creating the feeling that change was afoot. The midwives were also invited to come to QMMC to see the changes. The culture of change was projected toward the patients as well, to promote the perception that the launch of QRP represented quality improvement. Even cleaning of the delivery suites at QMMC was used as an opportunity to promote the new culture: the head of the Department cleaned alongside janitors to demonstrate dedication to service, high standards, collaboration, and teamwork. This was observed and cascaded out to the lying-in clinics as a part of “the new QRP culture." (Vergara et al 2020)* |
| 8F. When engaged and motivated network members are provided with the resources and opportunity to act (context) then they will attempt to enact changes in practices (outcome) because they feel empowered to change practice/work towards solving the problem (mechanism) | *"Between network inception and November 2016 hospitals have received 15 feedback reports. We observed that hospital teams are keen ‘not to be in the red’ but ‘be green’ in their report. Comparison of hospitals’ performance especially during face-to-face meetings helped foster shared learning rather than potentially harmful competition and helped people gain confidence in their ability to achieve change. We attribute this to having a shared vision and referring to poor performance as ‘offering opportunity for learning and improvement’ while engagement with peers provided encouragement to complete quality improvement processes.25 As a result, clinical teams worked with their records department and hospital administration, drawing lessons from across CIN, to ensure hospitals acquired infrastructure, reorganised service delivery and leadership provided effective mentorship and supervision to support delivery of quality care" (Irimu et al 2018)*  *“The ability of the network chair to engage the multidisciplinary clinical workforce in network initiatives, collaborate with external stakeholders, and to drive the implementation of network objectives was seen as critical for providing the foundation for the delivery of high-impact projects and creating a momentum towards successful network outcomes.” (McInnes et al 2015)* |

### Creating a psychological safe space

| 9A. When network leadership is available and approachable, invites input and feedback, and models openness, fallibility and non-judgmental behavior (context) then this promotes a network’s psychological safety (outcome) because network members feel empowered and not threatened when they speak up or make a mistake (mechanism) | *“In the case of QRP, the key transformation in trust required to make the Network of Care effective was between the midwives and Ob/Gyn staff. Several midwives described how, when accompanying a patient with an obstetric emergency to a tertiary care facility, they were escorted to a room for interrogation, were asked what they did to cause the emergencies, and were implored to produce their professional credentials, which were then confiscated. The obstetricians in QRP have admitted that they mistrusted the quality of care clients might receive in a private lying-in clinic. The founder of QRP herself admitted to this prejudice, and so made a point to travel to all the private lying-in clinics in the catchment area to see in person what the facilities were like and to meet the people running them. She states that, after making those in-person visits, her reservations about entering into a network agreement with the lying-in clinics dissipated.” (Vergara et al 2020)*  *“The network is led by inspiring, dynamic leaders. Many participants credited the leadership with creating a positive, empowering culture. Several participants described this as the result of equal and non-competing power dynamics, enabling the team to function optimally: “…we don’t have that hierarchical structure, we have more of a flat line I believe, when it comes to the allied health professionals and physicians in our model. There is that flat line respect and a flat line understanding that there’s a fit, you’re doing what is helping me, right. It’s like part of it, you’re part of the team” (Provider FG #2)”. (Sibbald et al 2020)*  *“At micro- level: absence of an enabling environment at facility level created a culture that gave little incentive for health facility staff to learn QI skills. It was further compounded by a rigid hierarchical system and partial administrative support especially encountered in the urban hub facility. Maintaining motivation in physicians was a challenge. Both the rural and urban facilities lacked dedicated hospital QI personnel, especially at the urban- hub facility; this made healthcare providers visualise QI as extra work, which distracted them from their regular patient care. Non- provision of transportation for hub mentors to their allocated spoke facilities added to poor motivation to undertake mentoring visits.” (Srivastava et al 2020)* |
| --- | --- |
| 9B. When network members form trusting and respectful horizontal relationships (leading to flattened hierarchy) (context), then this promotes a network’s psychological safety (outcome) because network members feel equal (mechanism) | *“The network is led by inspiring, dynamic leaders. Many participants credited the leadership with creating a positive, empowering culture. Several participants described this as the result of equal and non-competing power dynamics, enabling the team to function optimally: “…we don’t have that hierarchical structure, we have more of a flat line I believe, when it comes to the allied health professionals and physicians in our model. There is that flat line respect and a flat line understanding that there’s a fit, you’re doing what is helping me, right. It’s like part of it, you’re part of the team” (Provider FG #2).” (Sibbald et al 2020)*  *“It was clear that network members trusted one another and fostered psychological safety; members understood their role and felt safe to take risks (try new things, learn from mistakes). Psychological safety has been shown to drastically improve network performance and outcomes [32, 33]; in this case, it supported the implementation of innovative and emerging practices.” (Sibbald et al 2020)*  *“Regarding the training method, informants considered that the open attitude and absence of hierarchies shown by facilitators of the joint training meetings on maternal health, as well as including practical activities and resolution of clinical cases, enhanced dialogue, interest and involvement in the sessions. Thus, the initial defensiveness of primary care physicians was eliminated, a climate of equality (Table 4d) was fostered, and the resolution of doubts via direct communication between peers was promoted: “Now, I was able to stay in touch with a gynaecologist, and any particular doubt I could not solve by reading (regarding CPG), so I could trust them, I was able to detect a greater close relationship” (Professional Platform/Primary Care).” (Lopez-Vazquez et al 2021)*  *“Interdisciplinary and consumer collaboration was highly valued as a desirable outcome by those in network groups as it was seen as facilitating ‘new ways of working’, and providing ‘a voice for all disciplines’. These new ways of working in turn, promoted the sharing of knowledge and development of collaborations for improving patient outcomes: “Nurses and allied health particularly, have felt, probably to start with, intimidated by the process but now feel equal partners in it. So, that is a sign of success to me, before you even get to patient care.”” (McInnes et al 2012)*  **“***The development of interdisciplinary and consumer collaboration were thought by many participants across all stakeholder groups to have shifted some clinical groups from a stance that was often ‘competitive, defensive and protective’ to a more ‘co-operative and collegial ethos’. This ethos was said to have led to ‘a lot of renewed optimism’ in working in health care, which was also cited as a desirable outcome by one participant in the network participant group.” (McInnes et al 2012)*  *"In Kenya, health professionals often work in remote district hospitals, overseeing clinical departments with little support or training. Many find this difficult, particularly without resources to provide high standards of care. Consequently, loss of motivation and burnout are common (Brown, 2016; Mbindyo et al., 2009). CIN leaders attempted to create a supportive network community to addressed this problem, by providing physical and online spaces in which to meet and share as a professional community. CIN encourages network participants to think about new approaches to problem-solving, and then implement, test and measure solutions, and facilitates sharing of learning across participating organizations, thus functioning as an improvement collaborative (English, 2013)" (McGivern et al 2017)* |
| 9C. When a network has a shared network identity and culture that promotes a psychologically safe space (context) then network members are more likely able to learn, improve, and seek feedback (outcome) because they feel empowered and feel a reduced fear of negative consequences (mechanism) | *"Between network inception and November 2016 hospitals have received 15 feedback reports. We observed that hospital teams are keen ‘not to be in the red’ but ‘be green’ in their report. Comparison of hospitals’ performance especially during face-to-face meetings helped foster shared learning rather than potentially harmful competition and helped people gain confidence in their ability to achieve change. We attribute this to having a shared vision and referring to poor performance as ‘offering opportunity for learning and improvement’ while engagement with peers provided encouragement to complete quality improvement processes.25 As a result, clinical teams worked with their records department and hospital administration, drawing lessons from across CIN, to ensure hospitals acquired infrastructure, reorganised service delivery and leadership provided effective mentorship and supervision to support delivery of quality care" (Irimu et al 2018)*  *“Participants across all data sources discussed feeling empowered in their role in the network and the Lung Health program. The PCIC network members described being empowered through the trust that was developed that allowed them to take risks and try new things. One PCIC participant explained, “once we had some experience and some time working together and a trusting relationship, and that freeness and openness of sharing then it just sort of allowed us to grow, mature, and branch off into other areas.” (Provider FG#1). The RTs were empowered through training, collaboration, and mentorship to work independently to their full scope of practice allowing them to feel confident in their role. Further, the RTs ideas are reported in PCIC network meetings and contribute to network decision making. Other Providers felt empowered in their role collaborating with RTs to provide their patients with high quality respiratory care. Physicians trusted RTs to bring best practices into their clinic and support patient care.” (Sibbald et al 2020)* |
| 9D. When a network is a psychological safe space for network members (context) it enables members to openly raise concerns or problems (outcome) because they know they will be supported and there won’t be negative repercussions (mechanism) | *"Managers and providers were described, and saw themselves, as more accountable than before: The MRU has brought about ownership and accountability on the part of the managers and also the health professionals at facility level. Because even though we are not making it a whip to whip people, we [hold] people accountable. (District program manager) You start thinking now … if I am going to report in front of a group of people, why the baby died, I want to make sure that when I am on duty that baby is not going to die because I don’t want to go in the report. (Pediatric ward manager)" (Schneider et al 2020)*  *"Many CIN participants drew upon the visibility mechanisms of audit and data collection and related pastoral inspection practices to discipline themselves. Paediatrician 27 noted: “It has changed the way I practise … we were audited and we discovered we are actually not doing a hundred percent… previously we were not paying attention to detail but now we are.” Nurse-in-charge 14 commented: “Someone coming to check on what you are doing … When you have a trigger of somewhere somebody watching how do you do things, you become better and more conscious.” Paediatrician 13 noted: “CIN is like someone coming to audit me … an external supervisor … coming to see how things are being done … to make you rectify the wrong things … for me it is a fantastic thing.” "(McGivern et al 2017)*  **“***that the referral network provides or enhances service delivery to the population in two ways. Firstly, it facilitates access to specialist advice or Intensive Care Unit care which are not available at district level, or diagnostic services only offered centrally, such as computerized tomography scans or magnetic resonance imaging. This is in line with the intended scope of the referral network. RH respondents reported that the fact that district surgical teams are able to recognise their skills limits and refer patients is beneficial for the patient and the functionality of the care system.” (Pittalis et al 2021)* |
| 9E. When a network is a psychological safe space for network members (context), it encourages innovative behaviour and innovation (outcome) because they know they will be supported and there won’t be negative repercussions (mechanism) | *“Participants across all data sources discussed feeling empowered in their role in the network and the Lung Health program. The PCIC network members described being empowered through the trust that was developed that allowed them to take risks and try new things. One PCIC participant explained, “once we had some experience and some time working together and a trusting relationship, and that freeness and openness of sharing then it just sort of allowed us to grow, mature, and branch off into other areas.” (Provider FG#1). The RTs were empowered through training, collaboration, and mentorship to work independently to their full scope of practice allowing them to feel confident in their role.” (Sibbald et al 2020)* |
| 9F. When a network creates a psychological safe space for network members (context), it enables them to more easily collaborate across the network’s facilities, levels, and sectors of care (outcome) because they already have a common ground and understanding (mechanism) | *“In regard to informal (relational) coordination, the main finding of our research is that the operating environment within the hospital sector is not always conducive to collaborative work, and is permeated by mistrust between DLHs (afraid of reprimand) and RHs (fearing opportunistic behaviour by district clinicians). Blaming an individual hospital or clinician, and ignoring the systemic picture, will not improve the system. The combination of technical, organisational and human weaknesses in the referral system play a central role in the development of these patterns41 ; and when discretionary decisions occur, as shown in our findings, there are usually underlying factors such as lack of confidence and professional support, pressing workloads and resource constraints. Hence, addressing these challenges will require interventions at multiple levels.” (Pittalis et al 2021)* |
